# How are systematic reviews of prevalence conducted? A methodological study

**DOI:** 10.1186/s12874-020-00975-3

**Published:** 2020-04-26

**Authors:** Celina Borges Migliavaca, Cinara Stein, Verônica Colpani, Timothy Hugh Barker, Zachary Munn, Maicon Falavigna, on behalf of the Prevalence Estimates Reviews – Systematic Review Methodology Group (PERSyst)

**Affiliations:** 1 Programa de Pós-Graduação em Epidemiologia, Universidade Federal do Rio Grande do Sul, Porto Alegre, Rio Grande do Sul Brazil; 2 Hospital Moinhos de Vento, Porto Alegre, Rio Grande do Sul Brazil; 3 JBI, Faculty of Health Sciences, University of Adelaide, Adelaide, Australia; 4 Department of Health Research Methods, Evidence and Impact, McMaster University, Hamilton, ON Canada

**Keywords:** Prevalence, Systematic review, Methodological quality, Meta-epidemiological study

## Abstract

**Background:**

There is a notable lack of methodological and reporting guidance for systematic reviews of prevalence data. This information void has the potential to result in reviews that are inconsistent and inadequate to inform healthcare policy and decision making. The aim of this meta-epidemiological study is to describe the methodology of recently published prevalence systematic reviews.

**Methods:**

We searched MEDLINE (via PubMed) from February 2017 to February 2018 for systematic reviews of prevalence studies. We included systematic reviews assessing the prevalence of any clinical condition using patients as the unit of measurement and we summarized data related to reporting and methodology of the reviews.

**Results:**

A total of 235 systematic reviews of prevalence were analyzed. The median number of authors was 5 (interquartile range [IQR] 4–7), the median number of databases searched was 4 (3–6) and the median number of studies included in each review was 24 (IQR 15–41.5). Search strategies were presented for 68% of reviews. Forty five percent of reviews received external funding, and 24% did not provide funding information. Twenty three percent of included reviews had published or registered the systematic review protocol. Reporting guidelines were used in 72% of reviews. The quality of included studies was assessed in 80% of reviews. Nine reviews assessed the overall quality of evidence (4 using GRADE). Meta-analysis was conducted in 65% of reviews; 1% used Bayesian methods. Random effect meta-analysis was used in 94% of reviews; among them, 75% did not report the variance estimator used. Among the reviews with meta-analysis, 70% did not report how data was transformed; 59% percent conducted subgroup analysis, 38% conducted meta-regression and 2% estimated prediction interval; I^2^ was estimated in 95% of analysis. Publication bias was examined in 48%. The most common software used was STATA (55%).

**Conclusions:**

Our results indicate that there are significant inconsistencies regarding how these reviews are conducted. Many of these differences arose in the assessment of methodological quality and the formal synthesis of comparable data. This variability indicates the need for clearer reporting standards and consensus on methodological guidance for systematic reviews of prevalence data.

## Background

The proportion of a population currently suffering from a disease or particular condition of interest (prevalence) is an important [1] metric that allows researchers to assess disease burden, that is, who among the population is experiencing a certain disease, at a very specific point in time, typically measured using a cross-sectional study design [2]. The subsequent synthesis of this information in the form of a rigorously conducted and transparently reported systematic review has significant potential to better inform social and healthcare professionals, policy makers and consumers to better manage and plan for this disease burden [3].

**Fig. 1:**
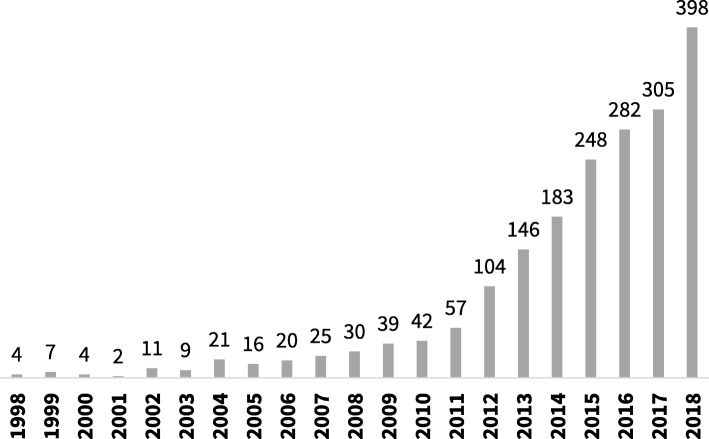
Number of systematic reviews of prevalence indexed in PubMed between 1998 and 2018

The number of systematic reviews of prevalence data has increased steadily over the last decade [4]. As can be seen in Fig. 1, a search of PubMed, conducted in October 2019 using the terms “systematic review” and “prevalence” in the title, identified a more than ten-fold increase in the number of reviews published from 2007 to 2017.

Despite this uptake and increased interest in the scientific community in the conduct of a systematic review of prevalence data, there remains little discourse on how these reviews should be conducted and reported. Although methodological guidance from the Joanna Briggs Institute (JBI) exists for these review types, [5] there currently appears to be little other discussion on how these reviews should be prepared, performed (including when and how results can be pooled using meta-analytical methods) and reported. This is an important consideration, as standards of reporting and conduct for traditional systematic reviews (i.e. systematic reviews of interventions) [6] are considered commonplace, and even endorsed by journal editors. Even though such standards and guidelines could be adapted for systematic reviews of prevalence, they do not comprehend specific issues for this type of study, and have not been as readily adopted. This is despite the fact that there is now general acceptance that there needs to be different types of approaches for systematic reviews looking at different types of evidence [6–8].

This lack of accepted, readily adopted and easy to implement methodological and reporting guidance for systematic reviews of prevalence data has the potential to result in inconsistent, varied, inadequate and potentially biased research conduct. Due to the particular importance of this kind of data in enabling health researchers and policy makers to quantify disease amongst populations, such research conducted without appropriate guidance is likely to have far-reaching and complicated consequences for the wider public community.

As such, the objective of this research project was to conduct a meta-epidemiological review of a sample of systematic reviews that have been published in peer-reviewed journals and evaluated a question regarding the prevalence of a certain disease, symptom or condition. This project allowed us to investigate how these reviews are conducted and to provide an overview of all methods utilized by systematic reviews authors asking a question of prevalence. The results of this project can potentially inform the development of future guidance for this review type.

## Methods

### Search strategy

To retrieve potentially relevant reviews, we searched MEDLINE (via PubMed) using the terms ‘systematic review’ and ‘prevalence’ in the title (*prevalence [TI] AND “systematic review”[TI] AND (“2017/02/01”[PDAT]: “2018/02/01”[PDAT])*). The search was limited to studies published between February 1st 2017 and February 1st 2018.

### Study selection and eligibility criteria

The selection of studies was conducted in two phases by two independent reviewers. First, we screened titles and abstracts. Then, we retrieved the full-text of potentially relevant studies to identify studies meeting our inclusion criteria.

We included systematic reviews of prevalence of any clinical condition, including diseases or symptoms. We excluded primary studies, letters, narrative reviews, systematic reviews of interventions or diagnostic accuracy, systematic reviews assessing the association between variables and systematic reviews of prevalence that did not use patients as the unit of measurement, as well as studies not published in English.

### Data abstraction

Using a standard and piloted form, one author extracted relevant data from each review and another author independently checked all data. Discrepancies were discussed and solved by consensus or by a third reviewer.

We abstracted the following data from individual studies: general information about the paper (number of authors, journal and year of publication), reporting of funding, reporting of search strategy, number and description of databases consulted, number of reviewers involved in each step of the review (study screening, selection, inclusion and data extraction), number of studies included in the review, methods for risk of bias appraisal and quality of evidence assessment, synthesis of results, assessment of publication bias and details of meta-analytic processes if meta-analysis was conducted (including variance estimator, transformation of data, heterogeneity assessment, and software used).

### Data analysis

Results are presented using descriptive statistics. Quantitative variables are presented as means and standard deviations or median and interquartile range, as appropriate; and qualitative variables are presented in absolute and relative frequencies.

## Results

**Fig. 2:**
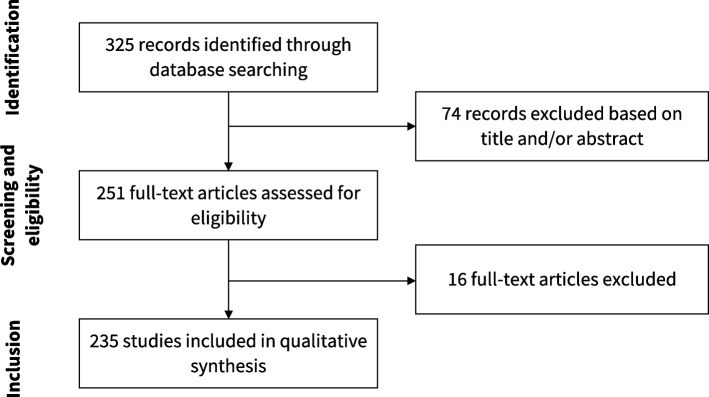
Flowchart of study selection

Our search resulted in 325 articles. We assessed the full text of 251 and included 235 of them in our analysis. Figure 2 presents the flowchart of study selection. The main characteristics of the included studies are described in Table 1. The complete list of included studies is presented in Additional file 1 and the list of full text excluded with reasons is presented in Additional file 2. The complete data extraction table is presented in Additional file 3.

**Table 1:** Main characteristics of included systematic reviews (*n* = 235)

Characteristic	Description
**Number of naming authors**	Median (IQR): 5 (4–7)
	Range: 1–18
**Protocol registry or publication**^**a**^	PROSPERO register: 51 (21.7%)
	Protocol published in peer-reviewed journal: 5 (2.1%)
	Not reported: 182 (77.4%)
**Use of a reporting guideline**^**a**^	PRISMA: 161 (68.5%)
	MOOSE: 27 (11.5%)
	GATHER: 2 (0.8%)
	Not reported: 65 (27.7%)
**External funding source**	Yes: 106 (45.1%)
	No: 73 (31.1%)
	Not reported: 56 (23.8%)
**Number of databases searched**	Median (IQR): 4 (3–6)
	Range: 1–14
**Databases searched**^**a**^	MEDLINE: 231 (98.3%)
	Embase: 146 (62.1%)
	Web of Science: 93 (39.6%)
	CENTRAL: 70 (29.8%)
	Scopus: 72 (30.6%)
	CINAHL: 61 (26%)
**Search strategy presented**	Full search strategy presented for at least one database: 159 (67.6%)
	Only presented terms used in the search (incomplete search strategy): 69 (29.4%)
	Nor reported: 7 (3.0%)
**Number of studies included in the review**	Median (IQR): 24 (15–41.5)
	Range: 2–1147
**Quality assessment of individual studies**^**a**^	JBI: 21 (8.9%)
	JBI (adapted): 5 (2.1%)
	Hoy, 2012: 10 (4.3%)
	Hoy, 2012 (adapted): 7 (3.0%)
	Loney, 1998: 6 (2.6%)
	Loney, 1998 (adapted): 2 (0.9%)
	Newcastle-Ottawa Scale: 10 (4.3%)
	Newcastle-Ottawa Scale (adapted): 13 (5.5%)
	Downs and Black (adapted): 2 (0.9%)
	STROBE: 15 (6.4%)
	STROBE (adapted): 7 (3.0%)
	New tool (not adaptation) specific for the review: 24 (10.2%)
	Non-validated tool, used by a similar review previously: 24 (10.2%)
	Others: 92 (39.1%)
	Not conducted: 47 (20%)
**Quality of the body of evidence**	GRADE: 4 (1.7%)
	Oxford: 1 (0.4%)
	Mean STROBE score: 1 (0.4%)
	JBI grades of recommendation: 1 (0.4%)
	AHCPR consistency of evidence: 1 (0.4%)
	Not conducted: 227 (96.6%)
**Data-synthesis**	Qualitative only: 83 (35.3%)
	Meta-analysis of prevalence data: 152 (64.7%)

^**a**^Adds to more than 100% because some reviews were counted in more than one option

Of 235 studies included, 83 (35.3%) presented narrative synthesis, without quantitative synthesis, while 152 (64.7%) performed meta-analysis. These 235 articles were published in 85 different journals. The leading publication journals (with 5 or more published reviews) were PLoS One, BMJ Open, Lancet Global Health and Medicine. The complete list of journals where included reviews were published is presented in Additional file 4.

Fifty-three studies (22.6%) published or registered a protocol, and 170 (72.3%) used a reporting guideline, including PRISMA (*n* = 161, 68.5%), MOOSE (*n* = 27, 11.5%) and GATHER (*n* = 2, 0.9%). In 56 studies (45.1%) there was no reporting of funding.

The median number of databases searched in each review was 4 (IQR 3–6); 231 studies (98.3%) used PubMed, 146 (62.1%) used Embase, 93 (39.6%) used Web of Science, and 70 (29.8%) used Cochrane CENTRAL, even though this is a database focused on interventional studies and systematic reviews. Most reviews (*n* = 228, 97.0%) reported the search strategy used, although 69 (29.4) reported it incompletely (presented the terms used, but not how the search strategy was designed). The median number of included original studies in each systematic review was 24 (IQR 15–41.5).

One hundred and eighty-eight reviews (80.0%) assessed the methodological quality of included studies. One hundred and five (44.7%) reported that this assessment was performed by at least two reviewers. There was a great variability regarding the instruments used to critically appraise included studies. Twenty-four studies (10.2%) developed new tools, and 24 (10.2%) used non-validated tools from previous similar systematic reviews. Among validated and specific tools to assess prevalence studies, the JBI prevalence critical appraisal tool was the most used (*n* = 21, 8.9%). Fifteen reviews (6.4%) used STROBE, a reporting guideline, to assess the methodological quality of included studies. Thirty-two reviews (17.0%) used assessment of methodological quality as an inclusion criterion for the review.

Nine studies (3.8%) assessed the quality of the body of evidence, with GRADE being the highest cited methodology (*n* = 4, 1.7%). Not all methods used to assess the quality of evidence were validated and appropriate. For instance, one study summarized the quality of evidence as the mean STROBE score of included studies.

### Statistical methods for meta-analysis

One hundred and fifty-two studies (64.7%) conducted meta-analysis to summarize prevalence estimates. The methods used by these reviews are summarized in Table 2.

**Table 2:** Methods used for meta-analysis (*n* = 152)

Characteristic	Description
**Methods approach**	Classic: 151 (99.3%)
	Bayesian: 1 (0.7%)
**Model**^**a**^	Random-effects: 141 (93.4%)
	Fixed-effects: 7 (4.6%)
	Other: 2 (1.3%)
	Not reported: 7 (4.6%)
**Variance estimator (for random-effect metanalysis,*n*** **= 141)**	DerSimonian and Laird: 30 (21.3%)
	Hartung-Knapp-Sidik-Jonkman: 4 (2.8%)
	Restricted maximum-likelihood: 1 (0.7%)
	Not reported: 106 (75.2%)
**Transformation**	Freeman-Tukey double arcsine: 32 (21.1%)
	Logit: 5 (3.3%)
	Log: 4 (2.6%)
	Raw: 2 (1.3%)
	Arcsine: 1 (0.7%)
	Arcsine square roots: 1 (0.7%)
	Not reported: 107 (70.4%)
**Heterogeneity assessment**^**a**^	Subgroup analysis: 89 (58.6%)
	Meta-regression: 57 (37.5%)
	I^2^: 144 (94.7%)
	Galbraith plot: 4 (2.6%)
	Other (eg. influence analysis, outliers): 54 (35.5%)
**Publication bias**	Begg’s test: 26 (17.1%)
	Egger test: 54 (35.5%)
	Funnel plot: 56 (36.8%)
	Doi plot: 4 (2.6%)
	Trim and fill: 7 (4.6%)
	LFK index: 4 (2.6%)
	Not reported: 79 (52.0%)
**Prediction interval**	Yes: 3 (2.0%)
	Not reported: 149 (98.0%)
**Software**^**a**^	STATA: 83 (54.6%)
	R: 29 (19.1%)
	Comprehensive Meta-analysis: 14 (9.2%)
	MetaXL: 11 (7.2%)
	MedCalc: 5 (3.3%)
	Review Manager: 3 (2.0%)
	Open Metanalyst: 3 (2.0%)
	StatsDirect: 3 (2.0%)
	MedScale: 1 (0.7%)
	Not reported: 5 (3.3%)

^**a**^Adds to more than 100% because some reviews were counted in more than one option

The vast majority of studies (n = 151, 99.3%) used classic methods instead of Bayesian approaches to pool prevalence estimates. The majority of studies pooled estimates using a random effects model (*n* = 141, 93.4%), and 7 studies (4.6%) utilized the fixed effect model. Two studies (1.3%) used the ‘quality model’, where the weight of each study was calculated based on a quality assessment. However, both reviews used non-validated methods to critically appraise included studies.

In relation to variance estimation in the reviews that conducted random effects meta-analysis, the DerSimonian and Laird method was used in 30 reviews (21.3%). However, 106 studies (75.2%) did not report the variance estimator used. Forty-five studies (29.6%) reported how they transformed the prevalence estimates, and the most used methods were Freeman-Tukey double arcsine (*n* = 32, 21.1%), logit (*n* = 5, 3.3%) and log (*n* = 4, 2.6%). Heterogeneity among studies was assessed with the I^2^ statistics in 114 studies (94.7%), meta-regression in 57 studies (37.5%) and subgroup analysis was performed in 89 studies (58.6%). Most analyses (*n* = 105, 76.1%) had an I^2^ estimate of 90% or more. Publication bias was assessed with funnel plots (*n* = 56, 36.8%) and Egger’s test (*n* = 54, 35.5%). Prediction interval was estimated in 3 reviews (2.0%).

## Discussion

This meta-epidemiological study identified 235 systematic reviews addressing a question related to the prevalence of any clinical condition. Our investigations have found that across the included systematic reviews of prevalence, there are significant and important discrepancies in how these reviews consider searching, risk of bias and data synthesis. In line with our results, a recently published study assessed a random sample of 215 systematic reviews of prevalence and cumulative incidence, and also found great heterogeneity among the methods used to conduct these reviews [7]. In our view, the study conducted by Hoffmann et al. and our study are complimentary. For instance, in the first one, the authors included reviews published in any year and compared the characteristics of reviews published before or after 2015 and with or without metanalysis; in our study, we assessed other methodological characteristics of the included reviews, specially related to the conduction of meta-analysis.

One area for potential guidance to inform future systematic reviews of prevalence data is in the risk of bias or critical appraisal stage. As can be seen from the results of our review, there are a number of checklists and tools that have been used. Some of the tools utilized were not appropriate for this assessment, such as the Newcastle Ottawa Scale (designed for cohort and case-control studies) [8] or STROBE (a reporting standard) [9]. Interestingly, some of the tools identified were designed specifically for studies reporting prevalence information, whilst other tools were adapted for this purpose, with the most adaptions to any one tool (*n* = 13, 5.5%) being made to the Newcastle Ottawa Scale. This is particularly concerning when reviews have used results of quality assessment to determine inclusion in the review, as was the case in 17% of the included studies. The combination of (a) using inadequate or inappropriate critical appraisal tools and (b) using the results of these tools to decide upon inclusion in a systematic review could lead to the inappropriate exclusion of relevant studies, which can alter the final results and produce misleading estimates. As such, there is an urgent need for the development and validation of a tool for assessing prevalence, along with endorsement and acceptance by the community to assist with standardization in this field. In the meantime, we urge reviewers to refer to the JBI critical appraisal tool, [4] which has been formally evaluated and is increasingly used across these types of reviews.

Encouragingly, 72.3% of the included reviews adhered to or cited a reporting guideline in their review. The main reporting guideline reported was the PRISMA statement [6]. However, this reporting guideline was designed particularly for reviews of interventions of therapies. As such, there have been multiple extensions to the original PRISMA statement for various review types [10, 11], yet no extension has yet been considered for systematic reviews of prevalence data. To ensure there is a reporting standard for use in prevalence systematic reviews, an extension or a broader version of the PRISMA statement including items important for this review type is recommended.

It was encouraging to see that multiple databases were often searched during the systematic review process, which is a recommendation for all review types. However, it is also important in systematic reviews that all the evidence is identified, and in the case of prevalence information, it may be particularly useful to search for data in unpublished sources, such as clinical registries, government reports, census data, and national administrative datasets, for example [12, 13]. However, there are no clearly established procedures on how to deal with this kind of information. Further guidance on searching for evidence in prevalence reviews is required.

In our review, we found 64.7% of reviewers conducted meta-analysis. There has been debate regarding the appropriateness of meta-analysis within systematic reviews of prevalence, [14, 15] [16–18] largely surrounding whether synthesizing across different populations is appropriate, as we reasonably expect prevalence rates to vary across different contexts and where different diagnostic criteria may be employed. However, meta-analysis, when done appropriately and using correct methodology, can provide important information regarding the burden of disease, including identifying differences amongst populations and regions, changes over time, and can provide a summarized estimate that can be used when calculating baseline risk, such as in GRADE summary of findings tables.

The vast majority of meta-analyses used classic methods and a random effects model, which is appropriate in these types of reviews [17] [18]. Although the common use of random effects across the reviews is encouraging, this is where the consistency ends, as we once again see considerable variability in the choice of methods to transform prevalence estimates for proportional meta-analysis. The most widely used approach was the Freeman-Tukey double arscine transformation. This has been recommended as the preferred methods for transformation [15, 18], although more recently it has come under question [19]. As such, further guidance and investigation into meta-analytical techniques is urgently required.

In reviews including meta-analysis, heterogeneity was assessed with the I^2^ in 94.7% of the included studies. Although I^2^ provides a useful indication of statistical heterogeneity amongst studies included in a meta-analysis, it can be misleading in cases where studies are providing large datasets with precise confidence intervals (such as in prevalence reviews) [20]. Other assessments of heterogeneity, such as T^2^ and prediction intervals, may be more appropriate in these types of reviews [20, 21]. Prediction intervals include the expected range of true effects in similar studies [22]. This is a more conservative way to incorporate uncertainty in the analysis when true heterogeneity is expected; however, it is still underused in meta-analysis of prevalence. Further guidance on assessing heterogeneity in these types of reviews is required.

Of the 235 systematic reviews analyzed, only 9 (3.8%) included a formal quality assessment or process to establish certainty of the entire body of evidence. Separate to critical appraisal, quality assessment of the entire body of evidence considers other factors in addition to methodological assessment that may impact on the subsequent recommendations drawn from such evidence [23]. Of these 9 reviews, only 4 (1.7%) followed the GRADE approach [24] which is now the commonplace methodology to reliably and sensibly rate the quality of the body of evidence. The considerably small number of identified reviews that included formal quality assessment of the entire body of evidence might be linked to the lack of formal guidance from the GRADE working group. The GRADE approach was developed to assess issues related to interventions (using evidence either from interventional or observational studies) and for diagnostic tests, however there are several extensions for the application of GRADE methodology. While there is no formal guidance for GRADE in systematic reviews of prevalence, there is some guidance into the use of GRADE for baseline risk or overall prognosis [25] which may be useful for these types of reviews. While no formal guidance exists, using the guidance cited above can serve as an interim method and is recommended by the authors for all future systematic reviews of prevalence. Whilst the number of identified studies that utilized GRADE in particular is small, it is encouraging to see the adoption of these methods in systematic reviews of prevalence data, as these examples will help contribute to the design of formal guidance for this data type in future.

It is reasonable to expect some differences in how these types of reviews are conducted, as different authors groups will rationally disagree regarding key issues, such as whether Bayesian approaches are best or which is the ideal method for transforming data. However, the wide inconsistency and variability noted in our review are far beyond the range of what could be considered reasonable and is of considerable concern for a number of reasons: this lack of standardization may encourage unnecessary duplication of reviews as reviewers approach similar questions with their own preferred methods; (2) novice reviewers searching for exemplar reviews may follow inadequate methods as they conduct these reviews; (3) the general confusion in end users, peer reviewers and readers of these reviews as they are required to become accustomed to various ways of conducting these studies; (4) review authors themselves following inadequate approaches, missing key steps or information sources, and importantly increasing the potential for review authors to report inaccurate or misleading summarized estimates; (5) a lack of standardization across reviews limits the ability to streamline, automate or use artificial intelligence to assist systematic review production, which is a burgeoning field of inquiry and research; and (6) these poorly conducted and reported reviews may have limited or even detrimental impacts on the planning and provision of healthcare. As such, we urgently call for the following to occur to rectify these issues, (1); further methods development in this field and for updated guidance on the conduct of these types of reviews, (2); we urgently require a reporting standard for these reviews (such as an extension to PRISMA), (4); the development (or endorsement) of a tailored risk of bias tool for studies reporting prevalence estimates, (5); the further development and promotion of software [26] and training materials [27] for these review types to support authors conducting these reviews.

### Limitations of our study

In this study, we have collated and interrogated the largest dataset of systematic reviews of prevalence currently available. Although only a sample, it is likely to be representative of all published prevalence systematic reviews, although it is important that we acknowledge we only searched MEDLINE over a period of 1 year using a search strategy that only retrieved studies with the terms “prevalence” and “systematic review” in the title. There is potential that systematic reviews of prevalence published in journals not indexed (or not published at all) are meaningfully different from those characterized in our results. However, given that reviews indexed on MEDLINE are (hypothetically) likely to be of higher quality than those not indexed, and given that we still identified substantial inconsistency, variability and potentially inappropriate practices in this sample, we doubt that a broader search will have altered our main conclusions significantly. Similarly, we believe that reviews that do not use the terms “prevalence” and “systematic review” in the title terms would have, overall, even more inappropriate methods. Regarding the timeframe limitation, we decided to include only reviews recently published because older reviews may not reflect the current practice.

## Conclusions

This meta-epidemiological review found that among this sample of published systematic reviews of prevalence, there are considerable discrepancies in terms of conduct, reporting, risk of bias assessment and data synthesis. This variability is understandable given the limited amount of guidance in this field, the lack of a reporting standard and a widely accepted risk of bias or critical appraisal tool. Our findings are a call to action to the evidence synthesis community to develop guidance and reporting standards urgently for these types of systematic reviews.

## Supplementary information

**Additional file 1.** Reference list of included articles.

**Additional file 2.** List of excluded articles, with reasons for exclusion.

**Additional file 3.** Complete data extraction table.

**Additional file 4.** Journals of publication of included systematic reviews.

## Data Availability

All data generated or analysed during this study are included in this published article and its supplementary information files.

## Abbreviations

AHCPR: Agency for Health Research and Quality
GATHER: Guidelines for Accurate and Transparent Health Estimates Reporting
GRADE: Grading of Recommendations Assessment, Development and Evaluation
IQR: Interquartile range
JBI: Joanna Briggs Institute
MOOSE: Meta-Analysis of Observational Studies in Epidemiology
PRISMA: Preferred Reporting Items for Systematic Reviews and Meta-Analyses
PROSPERO: International Prospective Register of Systematic Reviews
