# Supplementary material for: How are systematic reviews of prevalence conducted? A methodological study
Source: BMC Med Res Methodol. 2020 Apr 26;20:96. doi: 10.1186/s12874-020-00975-3 (PMC7184711; doi:10.1186/s12874-020-00975-3)
Supplement: Supplementary file 1 — Additional file 1. Reference list of included articles. [file 12874_2020_975_MOESM1_ESM.pdf]

Additional file 1: Complete references of included studies

1. Abd ElHafeez S, Bolignano D, D'Arrigo G, Dounousi E, Tripepi G, Zoccali C. Prevalence and burden of chronic kidney disease among the general population and high-risk groups in Africa: a systematic review. BMJ Open 2018;8:e015069. doi:10.1136/bmjopen-2016-015069.

2. Abuabara K, Yu AM, Okhovat J-P, Allen IE, Langan SM. The prevalence of atopic dermatitis beyond childhood: A systematic review and meta-analysis of longitudinal studies. Allergy 2017;73:696–704. doi:10.1111/all.13320.

3. Adeloye D, Ige JO, Aderemi AV, Adeleye N, Amoo EO, Auta A, et al. Estimating the prevalence, hospitalisation and mortality from type 2 diabetes mellitus in Nigeria: a systematic review and meta-analysis. BMJ Open 2017;7:e015424. doi:10.1136/bmjopen-2016-015424.

4. Akbari M, Moosazadeh M, Ghahramani S, Tabrizi R, Kolahdooz F, Asemi Z, et al. High prevalence of hypertension among Iranian children and adolescents. Journal of Hypertension 2017;35:1155–63. doi:10.1097/hjh.0000000000001261.

5. Akioyamen LE, Genest J, Shan SD, Reel RL, Albaum JM, Chu A, et al. Estimating the prevalence of heterozygous familial hypercholesterolaemia: a systematic review and meta-analysis. BMJ Open 2017;7:e016461. doi:10.1136/bmjopen-2017-016461.

6. Al-Moraissi EA, Perez D, Ellis E III. Do patients with malocclusion have a higher prevalence of temporomandibular disorders than controls both before and after orthognathic surgery? A systematic review and meta-analysis. Journal of Cranio-Maxillofacial Surgery 2017;45:1716–23. doi:10.1016/j.jcms.2017.07.015.

7. Alabdali A, Fisher JD, Trivedy C, Lilford RJ. A systematic review of the prevalence and types of adverse events in interfacility critical care transfers by paramedics. Air Medical Journal 2017;36:116–21. doi:10.1016/j.amj.2017.01.011.

8. Aleva FE, Voets LWLM, Simons SO, de Mast Q, van der Ven AJAM, Heijdra YF. Prevalence and localization of pulmonary embolism in unexplained acute exacerbations of COPD. Chest 2017;151:544–54. doi:10.1016/j.chest.2016.07.034.

9. Aliyu S, Smaldone A, Larson E. Prevalence of multidrug-resistant gram-negative bacteria among nursing home residents: A systematic review and meta-analysis. American Journal of Infection Control 2017;45:512–8. doi:10.1016/j.ajic.2017.01.022.

10. Alleblas CCJ, de Man AM, van den Haak L, Vierhout ME, Jansen FW, Nieboer TE. Prevalence of musculoskeletal disorders among surgeons performing minimally invasive surgery. Annals of Surgery 2017;266:905–20. doi:10.1097/sla.0000000000002223.

11. Almousa S, Bandin van Loon A. The prevalence of urinary incontinence in nulliparous adolescent and middle-aged women and the associated risk factors: A systematic review. Maturitas 2018;107:78–83. doi:10.1016/j.maturitas.2017.10.003.

12. Aluisio AR, Rege S, Stewart BT, Kinuthia J, Levine AC, Mello MJ, et al. Prevalence of HIV-seropositivity and associated impact on mortality among injured patients from low-and middle-income countries: A systematic review and meta-analysis. Current HIV Research 2017;15. doi:10.2174/1570162x15666170920112743.

13. Anderson FM, Hatch SL, Comacchio C, Howard LM. Prevalence and risk of mental disorders in the perinatal period among migrant women: a systematic review and meta-analysis. Archives of Women’s Mental Health 2017;20:449–62. doi:10.1007/s00737-017-0723-z.

14. Andrews P, Steultjens M, Riskowski J. Chronic widespread pain prevalence in the general population: A systematic review. European Journal of Pain 2017;22:5–18. doi:10.1002/ejp.1090.

15. Arabsalmani M, Behzadifar M, Baradaranmd HR, et al. Is Herpes Simplex virus (HSV) a sign of encephalitis in Iranian newborns? Prevalence of HSV infection in pregnant women in Iran: A systematic review and meta-analysis. Iranian Journal of Child Neurology. 2017;11(2):1-7.

16. Azizpour Y, Delpisheh A, Montazeri Z, Sayehmiri K. Prevalence of low back pain in Iranian nurses: a systematic review and meta-analysis. BMC Nursing 2017;16. doi:10.1186/s12912-017-0243-1.

17. Badawi A, Ryoo SG, Vasileva D, Yaghoubi S. Prevalence of chronic comorbidities in chikungunya: A systematic review and meta-analysis. International Journal of Infectious Diseases 2018;67:107–13. doi:10.1016/j.ijid.2017.12.018.

18. Beckwée D, Leysen L, Meuwis K, Adriaenssens N. Prevalence of aromatase inhibitor-induced arthralgia in breast cancer: a systematic review and meta-analysis. Supportive Care in Cancer 2017;25:1673–86. doi:10.1007/s00520-017-3613-z.

19. Bernard C, Dabis F, de Rekeneire N. Prevalence and factors associated with depression in people living with HIV in sub-Saharan Africa: A systematic review and meta-analysis. PLOS ONE 2017;12:e0181960. doi:10.1371/journal.pone.0181960.

20. Bi-Mohammed Z, Wright NM, Hearty P, King N, Gavin H. Prescription opioid abuse in prison settings: A systematic review of prevalence, practice and treatment responses. Drug and Alcohol Dependence 2017;171:122–31. doi:10.1016/j.drugalcdep.2016.11.032.

21. Bigna JJ, Kenne AM, Asangbeh SL, Sibetcheu AT. Prevalence of chronic obstructive pulmonary disease in the global population with HIV: a systematic review and meta-analysis. The Lancet Global Health 2018;6:e193–202. doi:10.1016/s2214-109x(17)30451-5.

22. Bigna JJ, Nansseu JR, Katte J-C, Noubiap JJ. Prevalence of prediabetes and diabetes mellitus among adults residing in Cameroon: A systematic review and meta-analysis. Diabetes Research and Clinical Practice 2018;137:109–18. doi:10.1016/j.diabres.2017.12.005.

23. Bigna JJ, Noubiap JJ, Nansseu JR, Aminde LN. Prevalence and etiologies of pulmonary hypertension in Africa: a systematic review and meta-analysis. BMC Pulmonary Medicine 2017;17. doi:10.1186/s12890-017-0549-5.

24. Bijlard E, Uiterwaal L, Kouwenberg CA, Mureau MA, Hovius SE, Huygen FJ. A Systematic Review on the Prevalence, Etiology, and Pathophysiology of Intrinsic Pain in Dermal Scar Tissue. Pain Physician 2017;20(2):1-13.

25. Bitta MA, Kariuki SM, Mwita C, Gwer S, Mwai L, Newton CRJC. Antimalarial drugs and the prevalence of mental and neurological manifestations: A systematic review and meta-analysis. Wellcome Open Research 2017;2:13. doi:10.12688/wellcomeopenres.10658.2.

26. Bourne RRA, Flaxman SR, Braithwaite T, Cicinelli MV, Das A, Jonas JB, et al. Magnitude, temporal trends, and projections of the global prevalence of blindness and distance and near vision impairment: a systematic review and meta-analysis. The Lancet Global Health 2017;5:e888–97. doi:10.1016/s2214-109x(17)30293-0.

27. Brinjikji W, Iyer VN, Wood CP, Lanzino G. Prevalence and characteristics of brain arteriovenous malformations in hereditary hemorrhagic telangiectasia: a systematic review and meta-analysis. Journal of Neurosurgery 2017;127:302–10. doi:10.3171/2016.7.jns16847.

28. Cagnazzo F, Gambacciani C, Morganti R, Perrini P. Intracranial aneurysms in patients with autosomal dominant polycystic kidney disease: prevalence, risk of rupture, and management. A systematic review. Acta Neurochirurgica 2017;159:811–21. doi:10.1007/s00701-017-3142-z.

29. Calado F, Alexandre J, Griffiths MD. Prevalence of adolescent problem gambling: A systematic review of recent research. Journal of Gambling Studies 2016;33:397–424. doi:10.1007/s10899-016-9627-5.

30. Casavechia MTG, de Melo GAN, Fernandes ACBS, de Castro KR, Pedroso RB, Santos TS, et al. Systematic review and meta-analysis on Schistosoma mansoni infection prevalence, and associated risk factors in Brazil. Parasitology 2018;145:1000–14. doi:10.1017/s0031182017002268.

31. Chaponda M, Aldhouse N, Kroes M, Wild L, Robinson C, Smith A. Systematic review of the prevalence of psychiatric illness and sleep disturbance as co-morbidities of HIV infection in the UK. International Journal of STD & AIDS 2018;29:704–13. doi:10.1177/0956462417750708.

32. Chen S-J, Shi L, Bao Y-P, Sun Y-K, Lin X, Que J-Y, et al. Prevalence of restless legs syndrome during pregnancy: A systematic review and meta-analysis. Sleep Medicine Reviews 2018;40:43–54. doi:10.1016/j.smrv.2017.10.003.

33. Chernet A, Utzinger J, Sydow V, Probst-Hensch N, Paris DH, Labhardt ND, et al. Prevalence rates of six selected infectious diseases among African migrants and refugees: a systematic review and meta-analysis. European Journal of Clinical Microbiology & Infectious Diseases 2017;37:605–19. doi:10.1007/s10096-017-3126-1.

34. Cheungpasitporn W, Thongprayoon C, Wijarnpreecha K, Mitema DG, Mao MA, Nissaisorakarn P, et al. Decline in prevalence and risk of helicobacter pylori in kidney transplant recipients: A systematic review and meta-analysis. Journal of Evidence-Based Medicine 2017;10:171–6. doi:10.1111/jebm.12252.

35. Coelho A, Crovella S. Microcephaly prevalence in infants born to Zika virus-infected women: A systematic review and meta-analysis. International Journal of Molecular Sciences 2017;18:1714. doi:10.3390/ijms18081714.

36. Dahlgren CL, Wisting L, Rø Ø. Feeding and eating disorders in the DSM-5 era: a systematic review of prevalence rates in non-clinical male and female samples. Journal of Eating Disorders 2017;5. doi:10.1186/s40337-017-0186-7.

37. Dalvand S, Niksima SH, Meshkani R, et al. Prevalence of metabolic syndrome among Iranian population: A systematic review and meta-analysis. Iranian Journal of Public Health 2017;46(4):456-467.

38. Degenhardt L, Peacock A, Colledge S, Leung J, Grebely J, Vickerman P, et al. Global prevalence of injecting drug use and sociodemographic characteristics and prevalence of HIV, HBV, and HCV in people who inject drugs: a multistage systematic review. The Lancet Global Health 2017;5:e1192–207. doi:10.1016/s2214-109x(17)30375-3.

39. Dekel S, Stuebe C, Dishy G. Childbirth induced posttraumatic stress syndrome: A systematic review of prevalence and risk factors. Frontiers in Psychology 2017;8. doi:10.3389/fpsyg.2017.00560.

40. Denfeld QE, Winters-Stone K, Mudd JO, Gelow JM, Kurdi S, Lee CS. The prevalence of frailty in heart failure: A systematic review and meta-analysis. International Journal of Cardiology 2017;236:283–9. doi:10.1016/j.ijcard.2017.01.153.

41. Deng N, Zhang X, Zhao F, Wang Y, He H. Prevalence of lipohypertrophy in insulin-treated diabetes patients: A systematic review and meta-analysis. Journal of Diabetes Investigation 2017;9:536–43. doi:10.1111/jdi.12742.

42. Dennis C-L, Falah-Hassani K, Shiri R. Prevalence of antenatal and postnatal anxiety: Systematic review and meta-analysis. British Journal of Psychiatry 2017;210:315–23. doi:10.1192/bjp.bp.116.187179.

43. Desikan P, Khan Z. Prevalence of hepatitis B and hepatitis C virus co-infection in India: A systematic review and meta-analysis. Indian Journal of Medical Microbiology 2017;35:332. doi:10.4103/ijmm.ijmm_17_257.

44. Dillon MP, Quigley M, Fatone S. A systematic review describing incidence rate and prevalence of dysvascular partial foot amputation; how both have changed over time and compare to transtibial amputation. Systematic Reviews 2017;6. doi:10.1186/s13643-017-0626-0.

45. Ding T, Hardiman PJ, Petersen I, Wang F-F, Qu F, Baio G. The prevalence of polycystic ovary syndrome in reproductive-aged women of different ethnicity: a systematic review and meta-analysis. Oncotarget 2017;8(56):96351-96358. doi:10.18632/oncotarget.19180.

46. Downs J, Blackmore AM, Epstein A, Skoss R, Langdon K, Jacoby P, et al. The prevalence of mental health disorders and symptoms in children and adolescents with cerebral palsy: a systematic review and meta-analysis. Developmental Medicine & Child Neurology 2017;60:30–8. doi:10.1111/dmcn.13555.

47. Dyer SM, Gomersall JS, Smithers LG, Davy C, Coleman DT, Street JM. Prevalence and characteristics of overweight and obesity in indigenous Australian children: A systematic review. Critical Reviews in Food Science and Nutrition 2015;57:1365–76. doi:10.1080/10408398.2014.991816.

48. Edwards J, Hayden J, Asbridge M, Gregoire B, Magee K. Prevalence of low back pain in emergency settings: a systematic review and meta-analysis. BMC Musculoskeletal Disorders 2017;18. doi:10.1186/s12891-017-1511-7.

49. Ejike CECC. Prevalence of hypertension in Nigerian children and adolescents: A systematic review and trend analysis of data from the past four decades. Journal of Tropical Pediatrics 2017:fmw087. doi:10.1093/tropej/fmw087.

50. Elbarazi I, Loney T, Yousef S, Elias A. Prevalence of and factors associated with burnout among health care professionals in Arab countries: a systematic review. BMC Health Services Research 2017;17. doi:10.1186/s12913-017-2319-8.

51. Elshahidi M, Elhadidi M, Sharaqi A, Mostafa A, Elzhery M. Prevalence of dementia in Egypt: a systematic review. Neuropsychiatric Disease and Treatment 2017;Volume 13:715–20. doi:10.2147/ndt.s127605.

52. Emaneini M, Beigverdi R, van Leeuwen WB, Rahdar H, Karami-Zarandi M, Hosseinkhani F, et al. Prevalence of methicillin-resistant Staphylococcus aureus isolated from burn patients in Iran: A systematic review and meta-analysis. Journal of Global Antimicrobial Resistance 2018;12:202–6. doi:10.1016/j.jgar.2017.10.015.

53. Emaneini M, Jabalameli F, van Leeuwen WB, Beigverdi R. Prevalence of group B Streptococcus in pregnant women in Iran. The Pediatric Infectious Disease Journal 2018;37:186–90. doi:10.1097/inf.0000000000001713.

54. Epstein S, Sparer EH, Tran BN, Ruan QZ, Dennerlein JT, Singhal D, et al. Prevalence of work-related musculoskeletal disorders among surgeons and interventionalists. JAMA Surgery 2018;153:e174947. doi:10.1001/jamasurg.2017.4947.

55. Escobar-Morreale HF, Santacruz E, Luque-Ramírez M, Botella Carretero JI. Prevalence of "obesity-associated gonadal dysfunction" in severely obese men and women and its resolution after bariatric surgery: a systematic review and meta-analysis. Human Reproduction Update 2017;23:390–408. doi:10.1093/humupd/dmx012.

56. Fan D, Li S, Wu S, Wang W, Ye S, Xia Q, et al. Prevalence of abnormally invasive placenta among deliveries in mainland China. Medicine 2017;96:e6636. doi:10.1097/md.0000000000006636.

57. Fang Q, Liu Z, Zhang Z, Zeng Y, Zhang T. Prevalence of Kaposi's sarcoma-associated herpesvirus among intravenous drug users: a systematic review and meta-analysis. Virologica Sinica 2017;32:415–22. doi:10.1007/s12250-017-4051-2.

58. Farahani M, Mulinder H, Farahani A, Marlink R. Prevalence and distribution of non-AIDS causes of death among HIV-infected individuals receiving antiretroviral therapy: a systematic review and meta-analysis. International Journal of STD & AIDS 2016;28:636–50. doi:10.1177/0956462416632428.

59. Farrag NS, Cheskin LJ, Farag MK. A systematic review of childhood obesity in the Middle East and North Africa (MENA) region: Prevalence and risk factors meta-analysis. Advances in Pediatric Research 2017;4. doi:10.12715/apr.2017.4.8.

60. Fishbain DA, Pulikal A, Lewis JE, Gao J. chronic pain types differ in their reported prevalence of post -traumatic stress disorder (PTSD) and there is consistent evidence that chronic pain is associated with PTSD: an evidence-based structured systematic review. Pain Medicine 2016:pnw065. doi:10.1093/pm/pnw065.

61. Fleming P, Bai JW, Pratt M, Sibbald C, Lynde C, Gulliver WP. The prevalence of anxiety in patients with psoriasis: a systematic review of observational studies and clinical trials. Journal of the European Academy of Dermatology and Venereology 2016;31:798–807. doi:10.1111/jdv.13891.

62. Foroutan M, Dalvand S, Khademvatan S, Majidiani H, Khalkhali H, Masoumifard S, et al. A systematic review and meta-analysis of the prevalence of Leishmania infection in blood donors. Transfusion and Apheresis Science 2017;56:544–51. doi:10.1016/j.transci.2017.07.001.

63. Frayman KB, Kazmerski TM, Sawyer SM. A systematic review of the prevalence and impact of urinary incontinence in cystic fibrosis. Respirology 2017;23:46–54. doi:10.1111/resp.13125.

64. French HP, Smart KM, Doyle F. Prevalence of neuropathic pain in knee or hip osteoarthritis: A systematic review and meta-analysis. Seminars in Arthritis and Rheumatism 2017;47:1–8. doi:10.1016/j.semarthrit.2017.02.008.

65. Fu X, Li Z-J, Yang C-J, Feng L, Sun L, Yao Y, et al. The prevalence of depression in rheumatoid arthritis in China: A systematic review. Oncotarget 2017;8. doi:10.18632/oncotarget.17323.

66. Fuglkjær S, Dissing KB, Hestbæk L. Prevalence and incidence of musculoskeletal extremity complaints in children and adolescents. A systematic review. BMC Musculoskeletal Disorders 2017;18. doi:10.1186/s12891-017-1771-2.

67. Gane EM, Michaleff ZA, Cottrell MA, McPhail SM, Hatton AL, Panizza BJ, et al. Prevalence, incidence, and risk factors for shoulder and neck dysfunction after neck dissection: A systematic review. European Journal of Surgical Oncology (EJSO) 2017;43:1199–218. doi:10.1016/j.ejso.2016.10.026.

68. Gerridzen IJ, Moerman-van den Brink WG, Depla MF, Verschuur EML, Veenhuizen RB, van der Wouden JC, et al. Prevalence and severity of behavioural symptoms in patients with Korsakoff syndrome and other alcohol-related cognitive disorders: a systematic review. International Journal of Geriatric Psychiatry 2016;32:256–73. doi:10.1002/gps.4636.

69. Ghaemmohamadi MS, Behzadifar M, Ghashghaee A, Mousavinejad N, Ebadi F, Saeedi Shahri SS, et al. Prevalence of depression in cardiovascular patients in Iran: A systematic review and meta-analysis from 2000 to 2017. Journal of Affective Disorders 2018;227:149–55. doi:10.1016/j.jad.2017.10.026.

70. Ghanei Gheshlagh R, Farajzadeh M, Zarei M, Baghi V, Dalvand S, Sayehmiri K, et al. The prevalence of restless legs syndrome in patients undergoing hemodialysis: A systematic review and meta-analysis study. Basic and Clinical Neuroscience Journal 2017;8:105–12. doi:10.18869/nirp.bcn.8.2.105.

71. Ghorbani NR, Djalalinia S, Modirian M, Abdar Z, Mansourian M, et al. Prevalence of hepatitis C infection in Iranian hemodialysis patients: An updated systematic review and meta-analysis. Journal of Research in Medical Sciences 2017;22:123. doi:10.4103/jrms.jrms_223_17.

72. Gilheaney Ó, Zgaga L, Harpur I, Sheaf G, Kiefer L, Béchet S, et al. The prevalence of oropharyngeal dysphagia in adults presenting with temporomandibular disorders associated with rheumatoid arthritis: A systematic review and meta-analysis. Dysphagia 2017;32:587–600. doi:10.1007/s00455-017-9808-0.

73. Gilheaney Ó, Béchet S, Kerr P, Kenny C, Smith S, Kouider R, et al. The prevalence of oral stage dysphagia in adults presenting with temporomandibular disorders: a systematic review and meta-analysis. Acta Odontologica Scandinavica 2018;76:448–58. doi:10.1080/00016357.2018.1424936.

74. Hackett KL, Gotts ZM, Ellis J, Deary V, Rapley T, Ng W-F, et al. An investigation into the prevalence of sleep disturbances in primary Sjögren's syndrome: a systematic review of the literature. Rheumatology 2016:kew443. doi:10.1093/rheumatology/kew443.

75. Harirchian MH, Fatehi F, Sarraf P, Honarvar NM, Bitarafan S. Worldwide prevalence of familial multiple sclerosis: A systematic review and meta-analysis. Multiple Sclerosis and Related Disorders 2018;20:43–7. doi:10.1016/j.msard.2017.12.015.

76. Harris R, Harman DJ, Card TR, Aithal GP, Guha IN. Prevalence of clinically significant liver disease within the general population, as defined by non-invasive markers of liver fibrosis: a systematic review. The Lancet Gastroenterology & Hepatology 2017;2:288–97. doi:10.1016/s2468-1253(16)30205-9.

77. Harrisson SA, Stynes S, Dunn KM, Foster NE, Konstantinou K. Neuropathic pain in low back-related leg pain patients: what is the evidence of prevalence, characteristics, and prognosis in primary care? A systematic review of the literature. The Journal of Pain 2017;18:1295–312. doi:10.1016/j.jpain.2017.04.012.

78. Hart HF, Stefanik JJ, Wyndow N, Machotka Z, Crossley KM. The prevalence of radiographic and MRI-defined patellofemoral osteoarthritis and structural pathology: a systematic review and meta-analysis. British Journal of Sports Medicine 2017;51:1195–208. doi:10.1136/bjsports-2017-097515.

79. Harvey L, Ludwig T, Hou AQ, Hock QS, Tan ML, Osatakul S, et al. Prevalence, cause and diagnosis of lactose intolerance in children aged 1-5 years: a systematic review of 1995-2015 literature. Asia Pacific Journal of Clinical Nutrition 2018;27(1):29-46 doi:10.6133/apjcn.022017.05

80. He S, Cao Y, Qin W, Chen W, Yin L, Chai H, et al. Prevalence of primary cardiac tumor malignancies in retrospective studies over six decades: a systematic review and meta-analysis. Oncotarget 2017;8. doi:10.18632/oncotarget.17378.

81. Heidari F, Afshari M, Moosazadeh M. Prevalence of fibromyalgia in general population and patients, a systematic review and meta-analysis. Rheumatology International 2017;37:1527–39. doi:10.1007/s00296-017-3725-2.

82. Heriseanu AI, Hay P, Corbit L, Touyz S. Grazing in adults with obesity and eating disorders: A systematic review of associated clinical features and meta-analysis of prevalence. Clinical Psychology Review 2017;58:16–32. doi:10.1016/j.cpr.2017.09.004.

83. Hofstraat SHI, Falla AM, Duffell EF, Hahné SJM, Amato-Gauci AJ, Veldhuijzen IK, et al. Current prevalence of chronic hepatitis B and C virus infection in the general population, blood donors and pregnant women in the EU/EEA: a systematic review. Epidemiology and Infection 2017;145:2873–85. doi:10.1017/s0950268817001947.

84. Hooi JKY, Lai WY, Ng WK, Suen MMY, Underwood FE, Tanyingoh D, et al. Global Prevalence of Helicobacter pylori Infection: Systematic Review and Meta-Analysis. Gastroenterology 2017;153:420–9. doi:10.1053/j.gastro.2017.04.022.

85. Hu C, Yu D, Sun X, Zhang M, Wang L, Qin H. The prevalence and progression of mild cognitive impairment among clinic and community populations: a systematic review and meta-analysis. International Psychogeriatrics 2017;29:1595–608. doi:10.1017/s1041610217000473.

86. Ilhan E, Chee E, Hush J, Moloney N. The prevalence of neuropathic pain is high after treatment for breast cancer. Pain 2017;158:2082–91. doi:10.1097/j.pain.0000000000001004.

87. Islam RM, Bell RJ, Davis SR. Prevalence of sexual symptoms in relation to menopause in women in Asia. Menopause 2017:1. doi:10.1097/gme.0000000000000967.

88. Ismail S, Karsenty G, Chartier-Kastler E, Cussenot O, Compérat E, Rouprêt M, et al. Prevalence, management, and prognosis of bladder cancer in patients with neurogenic bladder: A systematic review. Neurourology and Urodynamics 2017;37:1386–95. doi:10.1002/nau.23457.

89. Jawad M, Charide R, Waziry R, Darzi A, Ballout RA, Akl EA. The prevalence and trends of waterpipe tobacco smoking: A systematic review. Plos One 2018;13:e0192191. doi:10.1371/journal.pone.0192191.

90. Jimma W, Ghazisaeedi M, Shahmoradi L, Abdurahman AA, Kalhori SRN, Nasehi M, et al. Prevalence of and risk factors for multidrug-resistant tuberculosis in Iran and its neighboring countries: systematic review and meta-analysis. Revista da Sociedade Brasileira de Medicina Tropical 2017;50:287–95. doi:10.1590/0037-8682-0002-2017.

91. Jones KM, Balalla S, Theadom A, Jackman G, Feigin VL. A systematic review of the worldwide prevalence of survivors of poliomyelitis reported in 31 studies. BMJ Open 2017;7:e015470. doi:10.1136/bmjopen-2016-015470.

92. Jordan AE, Perlman DC, Neurer J, Smith DJ, Des Jarlais DC, Hagan H. Prevalence of hepatitis C virus infection among HIV+ men who have sex with men: a systematic review and meta-analysis. International Journal of STD & AIDS 2016;28:145–59. doi:10.1177/0956462416630910.

93. Jordan AE, Blackburn NA, Des Jarlais DC, Hagan H. Past-year prevalence of prescription opioid misuse among those 11 to 30 years of age in the United States: A systematic review and meta-analysis. Journal of Substance Abuse Treatment 2017;77:31–7. doi:10.1016/j.jsat.2017.03.007.

94. Kadir A, Mossey PA, Orth M, Blencowe H, Sowmiya M, Lawn JE, et al. Systematic Review and Meta-Analysis of the Birth Prevalence of Orofacial Clefts in Low- and Middle-Income Countries. The Cleft Palate-Craniofacial Journal 2017;54:571–81. doi:10.1597/15-221.

95. Kamalakannan S, Gudlavalleti AV, Gudlavalleti VM, Goenka S, Kuper H. Incidence and prevalence of stroke in India: A systematic review. Indian Journal of Medical Research 2017;146:175. doi:10.4103/ijmr.ijmr_516_15.

96. Karreman MC, Luime JJ, Hazes JMW, Weel AEAM. The prevalence and incidence of axial and peripheral spondyloarthritis in inflammatory bowel disease: A systematic review and meta-analysis. Journal of Crohn's and Colitis 2016:jjw199. doi:10.1093/ecco-jcc/jjw199.

97. Kassa GM, Muche AA, Berhe AK, Fekadu GA. Prevalence and determinants of anemia among pregnant women in Ethiopia; a systematic review and meta-analysis. BMC Hematology 2017;17. doi:10.1186/s12878-017-0090-z.

98. Kaze AD, Schutte AE, Erqou S, Kengne AP, Echouffo-Tcheugui JB. Prevalence of hypertension in older people in Africa. Journal of Hypertension 2017;35:1345–52. doi:10.1097/hjh.0000000000001345.

99. Keshavarz K, Angha P, Sayehmiri F, Sayemiri K, Yasemi M. The prevalence of visual disorders in Iranian students: A meta-analysis study and systematic review. Electronic Physician 2017;9:5516–24. doi:10.19082/5516.

100. Khalkhali HR, Foroutan M, Khademvatan S, Majidiani H, Aryamand S, Khezri P, et al. Prevalence of cystic echinococcosis in Iran: a systematic review and meta-analysis. Journal of Helminthology 2017;92:260–8. doi:10.1017/s0022149x17000463.

101. Kincaid DL, Doris M, Shannon C, Mulholland C. What is the prevalence of autism spectrum disorder and ASD traits in psychosis? A systematic review. Psychiatry Research 2017;250:99–105. doi:10.1016/j.psychres.2017.01.017.

102. King E, Steenson C, Shannon C, Mulholland C. Prevalence rates of childhood trauma in medical students: a systematic review. BMC Medical Education 2017;17. doi:10.1186/s12909-017-0992-2.

103. Kingston REF, Marel C, Mills KL. A systematic review of the prevalence of comorbid mental health disorders in people presenting for substance use treatment in Australia. Drug and Alcohol Review 2016;36:527–39. doi:10.1111/dar.12448.

104. Klem F, Wadhwa A, Prokop LJ, Sundt WJ, Farrugia G, Camilleri M, et al. Prevalence, risk factors, and outcomes of irritable bowel syndrome after infectious enteritis: A systematic review and meta-analysis. Gastroenterology 2017;152:1042–1054.e1. doi:10.1053/j.gastro.2016.12.039.

105. Knapik DM, Patel SH, Wetzel RJ, Voos JE. Prevalence and management of coracoid fracture sustained during sporting activities and time to return to sport: A systematic review. The American Journal of Sports Medicine 2017;46:753–8. doi:10.1177/0363546517718513.

106. Kojima G. Prevalence of frailty in end-stage renal disease: a systematic review and meta-analysis. International Urology and Nephrology 2017;49:1989–97. doi:10.1007/s11255-017-1547-5.

107. Kojima G, Iliffe S, Taniguchi Y, Shimada H, Rakugi H, Walters K. Prevalence of frailty in Japan: A systematic review and meta-analysis. Journal of Epidemiology 2017;27:347–53. doi:10.1016/j.je.2016.09.008.

108. Kolahdooz F, Sadeghirad B, Corriveau A, Sharma S. Prevalence of overweight and obesity among indigenous populations in Canada: A systematic review and meta-analysis. Critical Reviews in Food Science and Nutrition 2015;57:1316–27. doi:10.1080/10408398.2014.913003.

109. Kong L, Ma Q, Meng F, Cao J, Yu K, Shen Y. The prevalence of heterotopic ossification among patients after cervical artificial disc replacement. Medicine 2017;96:e7163. doi:10.1097/md.0000000000007163.

110. Kouidrat Y, Pizzol D, Cosco T, Thompson T, Carnaghi M, Bertoldo A, et al. High prevalence of erectile dysfunction in diabetes: a systematic review and meta-analysis of 145 studies. Diabetic Medicine 2017;34:1185–92. doi:10.1111/dme.13403.

111. Kouis P, Yiallouros PK, Middleton N, Evans JS, Kyriacou K, Papatheodorou SI. Prevalence of primary ciliary dyskinesia in consecutive referrals of suspect cases and the transmission electron microscopy detection rate: a systematic review and meta-analysis. Pediatric Research 2016;81:398–405. doi:10.1038/pr.2016.263.

112. Kunzmann AT, Graham S, McShane CM, Doyle J, Tommasino M, Johnston B, et al. The prevalence of viral agents in esophageal adenocarcinoma and Barrett's esophagus. European Journal of Gastroenterology & Hepatology 2017;29:817–25. doi:10.1097/meg.0000000000000868.

113. Laatikainen O, Miettunen J, Sneck S, Lehtiniemi H, Tenhunen O, Turpeinen M. The prevalence of medication-related adverse events in inpatients—a systematic review and meta-analysis. European Journal of Clinical Pharmacology 2017;73:1539–49. doi:10.1007/s00228-017-2330-3.

114. Lameijer CM, ten Duis HJ, Dusseldorp I van, Dijkstra PU, van der Sluis CK. Prevalence of posttraumatic arthritis and the association with outcome measures following distal radius fractures in non-osteoporotic patients: a systematic review. Archives of Orthopaedic and Trauma Surgery 2017;137:1499–513. doi:10.1007/s00402-017-2765-0.

115. Lan C-W, Scott-Sheldon LAJ, Carey KB, Johnson BT, Carey MP. Prevalence of alcohol use, sexual risk behavior, and HIV among Russians in high-risk settings: A systematic review and meta-analysis. International Journal of Behavioral Medicine 2016;24:180–90. doi:10.1007/s12529-016-9596-1.

116. Lange S, Probst C, Gmel G, Rehm J, Burd L, Popova S. Global prevalence of fetal alcohol spectrum disorder among children and youth. JAMA Pediatrics 2017;171:948. doi:10.1001/jamapediatrics.2017.1919.

117. Lange S, Probst C, Rehm J, Popova S. Prevalence of binge drinking during pregnancy by country and World Health Organization region: Systematic review and meta-analysis. Reproductive Toxicology 2017;73:214–21. doi:10.1016/j.reprotox.2017.08.004.

118. Lange S, Rehm J, Anagnostou E, Popova S. Prevalence of externalizing disorders and autism spectrum disorders among children with fetal alcohol spectrum disorder: Systematic review and meta-analysis. Biochemistry and Cell Biology 2018;96:241–51. doi:10.1139/bcb-2017-0014.

119. Łaszewska A, Österle A, Wancata J, Simon J. Prevalence of mental diseases in Austria. Wiener Klinische Wochenschrift 2018;130:141–50. doi:10.1007/s00508-018-1316-1.

120. Lee B, Lee SW, Kim DI, Kim JH. HPV prevalence in the foreskins of asymptomatic healthy infants and children: Systematic review and meta-analysis. Scientific Reports 2017;7. doi:10.1038/s41598-017-07506-z.

121. Leverment S, Clarke E, Wadeley A, Sengupta R. Prevalence and factors associated with disturbed sleep in patients with ankylosing spondylitis and non-radiographic axial spondyloarthritis: A systematic review. Rheumatology International 2016;37:257–71. doi:10.1007/s00296-016-3589-x.

122. Li D, Chen Q, Liu Y, Liu T, Tang W, Li S. The prevalence of acne in Mainland China: a systematic review and meta-analysis. BMJ Open 2017;7:e015354. doi:10.1136/bmjopen-2016-015354.

123. Lim YM, Song S, Song WO. Prevalence and determinants of overweight and obesity in children and adolescents from migrant and seasonal farmworker families in the United States—a systematic review and qualitative assessment. Nutrients 2017;9:188-205. doi:10.3390/nu9030188

124. Lin H, Zhang L, Zheng R, Zheng Y. The prevalence, metabolic risk and effects of lifestyle intervention for metabolically healthy obesity. Medicine 2017;96:e8838. doi:10.1097/md.0000000000008838.

125. Lorentzen TD, Subhi Y, Sørensen TL. Prevalence of polypoidal choroidal vasculopathy in white patients with exudative age-related macular degeneration. Retina 2017:1. doi:10.1097/iae.0000000000001872.

126. Lundorff M, Holmgren H, Zachariae R, Farver-Vestergaard I, O'Connor M. Prevalence of prolonged grief disorder in adult bereavement: A systematic review and meta-analysis. Journal of Affective Disorders 2017;212:138–49. doi:10.1016/j.jad.2017.01.030.

127. Alzahrani OH, Badahdah YS, Bamakrid MS, Alfayez AS, Alsaeedi MS, Mansouri AM, et al. The Diabetic Foot Research in Arabs' Countries. Open Journal of Endocrine and Metabolic Diseases 2013;3:157–65. doi:10.4236/ojemd.2013.33023.

128. Maroufizadeh S, Almasi-Hashiani A, Hosseini M, Sepidarkish M, Omani Samani R. Prevalence of diabetic retinopathy in Iran: A systematic review and meta-analysis. International Journal of Ophthalmology 2017;10(5):782-789. doi:10.18240/ijo.2017.05.21.

129. Maroufizadeh S, Almasi-Hashiani A, Omani Samani R, Sepidarkish M. Prevalence of retinopathy of prematurity in Iran: a systematic review and Meta-analysis. International Journal of Ophthalmology. 2017;10(8):1273-1279. doi:10.18240/ijo.2017.08.15.

130. McWilliams L, Farrell C, Grande G, Keady J, Swarbrick C, Yorke J. A systematic review of the prevalence of comorbid cancer and dementia and its implications for cancer-related care. Aging & Mental Health 2017:1–18. doi:10.1080/13607863.2017.1348476.

131. Medisauskaite A, Kamau C. Prevalence of oncologists in distress: Systematic review and meta-analysis. Psycho-Oncology 2017;26:1732–40. doi:10.1002/pon.4382.

132. Melese A, Demelash H. The prevalence of tuberculosis among prisoners in Ethiopia: a systematic review and meta-analysis of published studies. Archives of Public Health 2017;75. doi:10.1186/s13690-017-0204-x.

133. Minervini A, Campi R, Sessa F, Derweesh I, Kaouk JH, Mari A, et al. Positive surgical margins and local recurrence after simple enucleation and standard partial nephrectomy for malignant renal tumors: systematic review of the literature and meta-analysis of prevalence. Minerva Urologica e Nefrologica 2017;69(6):523-538. doi:10.23736/S0393-2249.17.02864-8.

134. Moghoofei M, Monavari SH, Mostafaei S, Hadifar S, Ghasemi A, Babaei F, et al. Prevalence of influenza A infection in the Middle-East: A systematic review and meta-analysis. The Clinical Respiratory Journal 2018;12:1787–801. doi:10.1111/crj.12758.

135. Moosazadeh M, Esmaeili R, Mehdi Nasehi M, et al. Prevalence of familial multiple sclerosis in Iran: A systematic review and meta-analysis. Iranian Journal of Neurology 2017;16(2):90-95.

136. Ashrafi E, Mohammadi S-F, Saeedi-Anari G, Mohammadi S-M, Farzadfar F, Lashay A, et al. Prevalence and major causes of visual impairment in Iranian adults: A systematic review. Middle East African Journal of Ophthalmology 2017;24:148. doi:10.4103/meajo.meajo_168_16.

137. Kazemi T, Mohseni J, Maleki M, Beydokhti H. A systematic review on the prevalence of acute myocardial infarction in Iran. Heart Views 2017;18:125. doi:10.4103/heartviews.heartviews_71_17.

138. Mokhayeri Y, Riahi SM, Rahimzadeh S, Pourhoseingholi MA, Hashemi-Nazari SS. Metabolic syndrome prevalence in the Iranian adult's general population and its trend: A systematic review and meta-analysis of observational studies. Diabetes & Metabolic Syndrome: Clinical Research & Reviews 2018;12:441–53. doi:10.1016/j.dsx.2017.12.023.

139. Molendijk ML, Montagne H, Bouachmir O, Alper Z, Bervoets J-P, Blom JD. Prevalence rates of the incubus phenomenon: A systematic review and meta-analysis. Frontiers in Psychiatry 2017;8. doi:10.3389/fpsyt.2017.00253.

140. Moosazadeh M, Abedi G, Afshari M, Mahdavi SA, Farshidi F, Kheradmand E. Prevalence of Enterobius vermicularis among children in Iran: A systematic review and meta-analysis. Osong Public Health and Research Perspectives 2017;8:108–15. doi:10.24171/j.phrp.2017.8.2.02.

141. Morais S, Costa AR, Ferro A, Lunet N, Peleteiro B. Contemporary migration patterns in the prevalence of Helicobacter pylori infection: A systematic review. Helicobacter 2017;22:e12372. doi:10.1111/hel.12372.

142. Moynan CR, McMillan TM. Prevalence of head injury and associated disability in prison populations. Journal of Head Trauma Rehabilitation 2017:1. doi:10.1097/htr.0000000000000354.

143. Mozhgani S-H, Zarei Ghobadi M, Moeini S, Pakzad R, Kananizadeh P, Behzadian F. Prevalence of human influenza virus in Iran: Evidence from a systematic review and meta-analysis. Microbial Pathogenesis 2018;115:168–74. doi:10.1016/j.micpath.2017.12.064.

144. Munabi NCO, Swanson J, Auslander A, Sanchez-Lara PA, Davidson Ward SL, Magee WP III. The prevalence of congenital heart disease in nonsyndromic cleft lip and/or palate. Annals of Plastic Surgery 2017;79:214–20. doi:10.1097/sap.0000000000001069.

145. Musa BM, Adamu AL, Galadanci NA, Zubayr B, Odoh CN, Aliyu MH. Trends in prevalence of multi drug resistant tuberculosis in sub-Saharan Africa: A systematic review and meta-analysis. Plos One 2017;12:e0185105. doi:10.1371/journal.pone.0185105.

146. Nascimento C, Di Lorenzo Alho AT, Bazan Conceição Amaral C, Leite REP, Nitrini R, Jacob-Filho W, et al. Prevalence of transactive response DNA-binding protein 43 (TDP-43) proteinopathy in cognitively normal older adults: systematic review and meta-analysis. Neuropathology and Applied Neurobiology 2017;44:286–97. doi:10.1111/nan.12430.

147. Ng SC, Shi HY, Hamidi N, Underwood FE, Tang W, Benchimol EI, et al. Worldwide incidence and prevalence of inflammatory bowel disease in the 21st century: a systematic review of population-based studies. The Lancet 2017;390:2769–78. doi:10.1016/s0140-6736(17)32448-0.

148. Nguyen GT, Phan K, Teng I, Pu J, Watanabe T. A systematic review and meta-analysis of the prevalence of norovirus in cases of gastroenteritis in developing countries. Medicine 2017;96:e8139. doi:10.1097/md.0000000000008139.

149. Nilsson C, Hessman E, Sjöblom H, Dencker A, Jangsten E, Mollberg M, et al. Definitions, measurements and prevalence of fear of childbirth: a systematic review. BMC Pregnancy and Childbirth 2018;18. doi:10.1186/s12884-018-1659-7.

150. Noubiap JJ, Essouma M, Bigna JJ, Jingi AM, Aminde LN, Nansseu JR. Prevalence of elevated blood pressure in children and adolescents in Africa: a systematic review and meta-analysis. The Lancet Public Health 2017;2:e375–86. doi:10.1016/s2468-2667(17)30123-8.

151. O'Connell MA, Leahy-Warren P, Khashan AS, Kenny LC, O'Neill SM. Worldwide prevalence of tocophobia in pregnant women: systematic review and meta-analysis. Acta Obstetricia et Gynecologica Scandinavica 2017;96:907–20. doi:10.1111/aogs.13138.

152. Onyedum CC, Alobu I, Ukwaja KN. Prevalence of drug-resistant tuberculosis in Nigeria: A systematic review and meta-analysis. Plos One 2017;12:e0180996. doi:10.1371/journal.pone.0180996.

153. Oosterwijk AM, Mouton LJ, Schouten H, Disseldorp LM, van der Schans CP, Nieuwenhuis MK. Prevalence of scar contractures after burn: A systematic review. Burns 2017;43:41–9. doi:10.1016/j.burns.2016.08.002.

154. Oqab Z, Ganshorn H, Sheldon R. Prevalence of pulmonary embolism in patients presenting with syncope. A systematic review and meta-analysis. The American Journal of Emergency Medicine 2018;36:551–5. doi:10.1016/j.ajem.2017.09.015.

155. Osinubi O, Grainge MJ, Hong L, Ahmed A, Batchelor JM, Grindlay D, et al. The prevalence of psychological comorbidity in people with vitiligo: a systematic review and meta-analysis. British Journal of Dermatology 2018;178:863–78. doi:10.1111/bjd.16049.

156. Ouattara AK, Yameogo P, Traore L, Diarra B, Assih M, Compaore TR, et al. Prevalence, genetic variants and clinical implications of G-6-PD deficiency in Burkina Faso: a systematic review. BMC Medical Genetics 2017;18. doi:10.1186/s12881-017-0496-2.

157. Oudshoorn BY, Driscoll H, Kilner K, Dunn M, James D. Prevalence of laceration injuries in professional and amateur rugby union: a systematic review and meta-analysis. BMJ Open Sport & Exercise Medicine 2017;3:e000239. doi:10.1136/bmjsem-2017-000239.

158. Özcan NK, Boyacıoğlu NE, Dinç H. Postpartum depression prevalence and risk factors in Turkey: A systematic review and meta-analysis. Archives of Psychiatric Nursing 2017;31:420–8. doi:10.1016/j.apnu.2017.04.006.

159. Pamoukdjian F, Bouillet T, Lévy V, Soussan M, Zelek L, Paillaud E. Prevalence and predictive value of pre-therapeutic sarcopenia in cancer patients: A systematic review. Clinical Nutrition 2018;37:1101–13. doi:10.1016/j.clnu.2017.07.010.

160. Parola V, Coelho A, Cardoso D, Sandgren A, Apóstolo J. Prevalence of burnout in health professionals working in palliative care. JBI Database of Systematic Reviews and Implementation Reports 2017;15:1905–33. doi:10.11124/jbisrir-2016-003309.

161. Pasha YZ, Vahedi A, Zamani M, Alizadeh-Navaei R, Pasha EZ. Prevalence of birth defects in iran: A systematic review and meta-analysis. Archives of Iranian Medicine 2017 1;20(6):376-385.

162. Paz SPC, Branco L, Pereira MA de C, Spessotto C, Fragoso YD. Systematic review of the published data on the worldwide prevalence of John Cunningham virus in patients with multiple sclerosis and neuromyelitis optica. Epidemiology and Health 2018;40:e2018001. doi:10.4178/epih.e2018001.

163. Pereira L, Monyror J, Almeida FT, Almeida FR, Guerra E, Flores-Mir C, et al. Prevalence of adenoid hypertrophy: A systematic review and meta-analysis. Sleep Medicine Reviews 2018;38:101–12. doi:10.1016/j.smrv.2017.06.001.

164. Perrin NE, Davies MJ, Robertson N, Snoek FJ, Khunti K. The prevalence of diabetes-specific emotional distress in people with Type 2 diabetes: a systematic review and meta-analysis. Diabetic Medicine 2017;34:1508–20. doi:10.1111/dme.13448.

165. Pestana PM, Vaz-Freitas S, Manso MC. Prevalence of voice disorders in singers: systematic review and meta-analysis. Journal of Voice 2017;31:722–7. doi:10.1016/j.jvoice.2017.02.010.

166. Popova S, Lange S, Probst C, Gmel G, Rehm J. Estimation of national, regional, and global prevalence of alcohol use during pregnancy and fetal alcohol syndrome: a systematic review and meta-analysis. The Lancet Global Health 2017;5:e290–9. doi:10.1016/s2214-109x(17)30021-9.

167. Porter LH, Lawrence MG, Ilic D, Clouston D, Bolton DM, Frydenberg M, et al. Systematic review links the prevalence of intraductal carcinoma of the prostate to prostate cancer risk categories. European Urology 2017;72:492–5. doi:10.1016/j.eururo.2017.03.013.

168. Porto De Toledo I, Stefani FM, Porporatti AL, Mezzomo LA, Peres MA, Flores-Mir C, et al. Prevalence of otologic signs and symptoms in adult patients with temporomandibular disorders: a systematic review and meta-analysis. Clinical Oral Investigations 2016;21:597–605. doi:10.1007/s00784-016-1926-9.

169. Qin J-B, Sheng X-Q, Wu D, Gao S-Y, You Y-P, Yang T-B, et al. Worldwide prevalence of adverse pregnancy outcomes among singleton pregnancies after in vitro fertilization/intracytoplasmic sperm injection: a systematic review and meta-analysis. Archives of Gynecology and Obstetrics 2016;295:285–301. doi:10.1007/s00404-016-4250-3.

170. Qin J-B, Sheng X-Q, Wang H, Chen G-C, Yang J, Yu H, et al. Worldwide prevalence of adverse pregnancy outcomes associated with in vitro fertilization/intracytoplasmic sperm injection among multiple births: a systematic review and meta-analysis based on cohort studies. Archives of Gynecology and Obstetrics 2017;295:577–97. doi:10.1007/s00404-017-4291-2.

171. Rabbitts JA, Fisher E, Rosenbloom BN, Palermo TM. Prevalence and predictors of chronic postsurgical pain in children: A systematic review and meta-analysis. The Journal of Pain 2017;18:605–14. doi:10.1016/j.jpain.2017.03.007.

172. Rakesh PS. Prevalence of anaemia in Kerala State, Southern India - A systematic review. Journal of Clinical And Diagnostic Research 2017. doi:10.7860/jcdr/2017/24681.9951.

173. Ramírez J, Nieto-González JC, Curbelo Rodríguez R, Castañeda S, Carmona L. Prevalence and risk factors for osteoporosis and fractures in axial spondyloarthritis: A systematic review and meta-analysis. Seminars in Arthritis and Rheumatism 2018;48:44–52. doi:10.1016/j.semarthrit.2017.12.001.

174. Ranjbaran M, Omani Samani R, Almasi-Hashiani A, Matourypour P, Moini A. Prevalence of premenstrual syndrome in Iran: A systematic review and meta-analysis. International Journal of Reproductive Biomedicine 2017;15(11):679-686.

175. Ravaghi H, Behzadifar M, Behzadifar M, Mirghaed MT, Aryankhesal A, Salemi M, et al. Prevalence of depression in hemodialysis patients in Iran: A systematic review and meta-analysis. Iranian Journal of Kidney Diseases 2017;11(2):90-98.

176. Rees F, Doherty M, Grainge MJ, Lanyon P, Zhang W. The worldwide incidence and prevalence of systemic lupus erythematosus: a systematic review of epidemiological studies. Rheumatology 2017;56:1945–61. doi:10.1093/rheumatology/kex260.

177. Rezaei S, Hajizadeh M, Zandian H, Fathi A, Nouri B. Period prevalence and reporting rate of needlestick injuries to nurses in Iran: A systematic review and meta-analysis. Research in Nursing & Health 2017;40:311–22. doi:10.1002/nur.21801.

178. Rezaei S, Karami Matin B, Hajizadeh M, Soroush A, Nouri B. Prevalence of burnout among nurses in Iran: a systematic review and meta-analysis. International Nursing Review 2018;65:361–9. doi:10.1111/inr.12426.

179. Ribeiro RVE. Prevalence of body dysmorphic disorder in plastic surgery and dermatology patients: A systematic review with meta-analysis. Aesthetic Plastic Surgery 2017;41:964–70. doi:10.1007/s00266-017-0869-0.

180. Rich NE, Oji S, Mufti AR, Browning JD, Parikh ND, Odewole M, et al. Racial and ethnic disparities in nonalcoholic fatty liver disease prevalence, severity, and outcomes in the United States: A systematic review and meta-analysis. Clinical Gastroenterology and Hepatology 2018;16:198–210.e2. doi:10.1016/j.cgh.2017.09.041.

181. Robertson J, Chadwick D, Baines S, Emerson E, Hatton C. Prevalence of dysphagia in people with intellectual disability: A systematic review. Intellectual and Developmental Disabilities 2017;55:377–91. doi:10.1352/1934-9556-55.6.377.

182. Rouhi A, Hazlewood G, Shaheen AA, Swain MG, Barber CEH. Prevalence and risk factors for liver fibrosis detected by transient elastography or shear wave elastography in inflammatory arthritis: A systematic review. Clinical and experimental rheumatology 2017;35(6):1029-36.

183. Salmanian M, Asadian-koohestani F, Mohammadi MR. A systematic review on the prevalence of conduct disorder in the Middle East. Social Psychiatry and Psychiatric Epidemiology 2017;52:1337–43. doi:10.1007/s00127-017-1414-9.

184. Schnippel K, Firnhaber C, Berhanu R, Page-Shipp L, Sinanovic E. Adverse drug reactions during drug-resistant TB treatment in high HIV prevalence settings: a systematic review and meta-analysis. Journal of Antimicrobial Chemotherapy 2017;72:1871–9. doi:10.1093/jac/dkx107.

185. Scholz-Kreisel P, Spix C, Blettner M, Eckerle S, Faber J, Wild P, et al. Prevalence of cardiovascular late sequelae in long-term survivors of childhood cancer: A systematic review and meta-analysis. Pediatric Blood & Cancer 2017;64:e26428. doi:10.1002/pbc.26428.

186. Scotti L, Franchi M, Marchesoni A, Corrao G. Prevalence and incidence of psoriatic arthritis: A systematic review and meta-analysis. Seminars in Arthritis and Rheumatism 2018;48:28–34. doi:10.1016/j.semarthrit.2018.01.003.

187. Senaratna CV, Perret JL, Lodge CJ, Lowe AJ, Campbell BE, Matheson MC, et al. Prevalence of obstructive sleep apnea in the general population: A systematic review. Sleep Medicine Reviews 2017;34:70–81. doi:10.1016/j.smrv.2016.07.002.

188. Shafiee G, Keshtkar A, Soltani A, Ahadi Z, Larijani B, Heshmat R. Prevalence of sarcopenia in the world: a systematic review and meta- analysis of general population studies. Journal of Diabetes & Metabolic Disorders 2017;16. doi:10.1186/s40200-017-0302-x.

189. Shah A, Shanahan E, Macdonald G, Fletcher L, Ghasemi P, Morrison M, et al. Systematic review and meta-analysis: Prevalence of small intestinal bacterial overgrowth in chronic liver disease. Seminars in Liver Disease 2017;37:388–400. doi:10.1055/s-0037-1608832.

190. ShamsHosseini N, Vahdati T, Mohammadzadeh Z, Yeganeh A, Davoodi S. Prevalence of musculoskeletal disorders among dentists in Iran: A systematic review. Materia Socio Medica 2017;29:257. doi:10.5455/msm.2017.29.257-262.

191. Sharifi N, Khazayian S, Pakzad R, Fathnezhad Kazemi A, Chehreh H. Investigating the prevalence of preterm delivery in Iranian population: A systematic review and meta-analysis. Journal of Caring Sciences 2017;6:371–80. doi:10.15171/jcs.2017.035.

192. Shaw L, Morozova M, Abu-Arafeh I. Chronic post-traumatic headache in children and adolescents: systematic review of prevalence and headache features. Pain Management 2018;8:57–64. doi:10.2217/pmt-2017-0019.

193. Shawon MSR, Perret JL, Senaratna CV, Lodge C, Hamilton GS, Dharmage SC. Current evidence on prevalence and clinical outcomes of co-morbid obstructive sleep apnea and

chronic obstructive pulmonary disease: A systematic review. Sleep Medicine Reviews 2017;32:58–68. doi:10.1016/j.smrv.2016.02.007.

194. Silva MF, Leite FRM, Ferreira LB, Pola NM, Scannapieco FA, Demarco FF, et al. Estimated prevalence of halitosis: a systematic review and meta-regression analysis. Clinical Oral Investigations 2017;22:47–55. doi:10.1007/s00784-017-2164-5.

195. Smith BE, Selfe J, Thacker D, Hendrick P, Bateman M, Moffatt F, et al. Incidence and prevalence of patellofemoral pain: A systematic review and meta-analysis. Plos One 2018;13:e0190892. doi:10.1371/journal.pone.0190892.

196. Smythe T, Kuper H, Macleod D, Foster A, Lavy C. Birth prevalence of congenital talipes equinovarus in low- and middle-income countries: a systematic review and meta-analysis. Tropical Medicine & International Health 2017;22:269–85. doi:10.1111/tmi.12833.

197. So YK, Kim M-J, Kim S, Son Y-I. Lateral lymph node metastasis in papillary thyroid carcinoma: A systematic review and meta-analysis for prevalence, risk factors, and location. International Journal of Surgery 2018;50:94–103. doi:10.1016/j.ijsu.2017.12.029.

198. Song P, Chang X, Wang M, An L. Variations of pterygium prevalence by age, gender and geographic characteristics in China: A systematic review and meta-analysis. Plos One 2017;12:e0174587. doi:10.1371/journal.pone.0174587.

199. Song P, Liu Y, Yu X, Wu J, Poon AN, Demaio A, et al. Prevalence of epilepsy in China between 1990 and 2015: A systematic review and meta–analysis. Journal of Global Health 2017;7. doi:10.7189/jogh.07.020706.

200. Song P, Wang J, Wei W, Chang X, Wang M, An L. The prevalence of vitamin A deficiency in Chinese children: A systematic review and bayesian meta-analysis. Nutrients 2017;9:1285. doi:10.3390/nu9121285.

201. Spottswood M, Davydow DS, Huang H. The prevalence of posttraumatic stress disorder in primary care. Harvard Review of Psychiatry 2017:1. doi:10.1097/hrp.0000000000000136.

202. Stockdale AJ, Chaponda M, Beloukas A, Phillips RO, Matthews PC, Papadimitropoulos A, et al. Prevalence of hepatitis D virus infection in sub-Saharan Africa: a systematic review and meta-analysis. The Lancet Global Health 2017;5:e992–1003. doi:10.1016/s2214-109x(17)30298-x.

203. Strasser L, Downes M, Kung J, Cross JH, De Haan M. Prevalence and risk factors for autism spectrum disorder in epilepsy: a systematic review and meta-analysis. Developmental Medicine & Child Neurology 2017;60:19–29. doi:10.1111/dmcn.13598.

204. Surda P, Walker A, Putala M, Siarnik P. Prevalence of rhinitis in athletes: Systematic review. International Journal of Otolaryngology 2017;2017:1–5. doi:10.1155/2017/8098426.

205. Sy E, Sklar MC, Lequier L, Fan E, Kanji HD. Anticoagulation practices and the prevalence of major bleeding, thromboembolic events, and mortality in venoarterial extracorporeal

membrane oxygenation: A systematic review and meta-analysis. Journal of Critical Care 2017;39:87–96. doi:10.1016/j.jcrc.2017.02.014.

206. Tabrizi R, Moosazadeh M, Razzaghi A, et al. Prevalence of sleep quality disorder among Iranian drivers: a systematic review and meta-analysis. Journal of Injury and Violence Research 2018;10(1):53-59. doi:10.5249/jivr.v10i1.993.

207. Tavares AM, Fronteira I, Couto I, Machado D, Viveiros M, Abecasis AB, et al. HIV and tuberculosis co-infection among migrants in Europe: A systematic review on the prevalence, incidence and mortality. Plos One 2017;12:e0185526. doi:10.1371/journal.pone.0185526.

208. Tengan FM, Abdala E, Nascimento M, Bernardo WM, Barone AA. Prevalence of hepatitis B in people living with HIV/AIDS in Latin America and the Caribbean: a systematic review and meta-analysis. BMC Infectious Diseases 2017;17. doi:10.1186/s12879-017-2695-z.

209. Théroux J, Stomski N, Hodgetts CJ, Ballard A, Khadra C, Le May S, et al. Prevalence of low back pain in adolescents with idiopathic scoliosis: a systematic review. Chiropractic & Manual Therapies 2017;25. doi:10.1186/s12998-017-0143-1.

210. Trompeter K, Fett D, Platen P. Prevalence of back pain in sports: A systematic review of the literature. Sports Medicine 2016;47:1183–207. doi:10.1007/s40279-016-0645-3.

211. Tubaishat A, Papanikolaou P, Anthony D, Habiballah L. Pressure ulcers prevalence in the acute care setting: A systematic review, 2000-2015. Clinical Nursing Research 2017;27:643–59. doi:10.1177/1054773817705541.

212. Umer W, Antwi-Afari MF, Li H, Szeto GPY, Wong AYL. The prevalence of musculoskeletal symptoms in the construction industry: a systematic review and meta-analysis. International Archives of Occupational and Environmental Health 2017;91:125–44. doi:10.1007/s00420-017-1273-4.

213. VanderKruik R, Barreix M, Chou D, Allen T, Say L, et al. The global prevalence of postpartum psychosis: a systematic review. BMC Psychiatry 2017;17. doi:10.1186/s12888-017-1427-7.

214. Vikse J, Sanna B, Henry BM, Taterra D, Sanna S, Pękala PA, et al. The prevalence and morphometry of an accessory spleen: A meta-analysis and systematic review of 22,487 patients. International Journal of Surgery 2017;45:18–28. doi:10.1016/j.ijsu.2017.07.045.

215. Vitalis A, Lip GYH, Kay M, Vohra RK, Shantsila A. Ethnic differences in the prevalence of peripheral arterial disease: A systematic review and meta-analysis. Expert Review of Cardiovascular Therapy 2017;15:327–38. doi:10.1080/14779072.2017.1305890.

216. Wang Z-D, Liu Q, Liu H-H, Li S, Zhang L, Zhao Y-K, et al. Prevalence of Cryptosporidium, microsporidia and Isospora infection in HIV-infected people: a global systematic review and meta-analysis. Parasites & Vectors 2018;11. doi:10.1186/s13071-017-2558-x.

217. Wang C, Nishiyama T, Kikuchi S, Inoue M, Sawada N, Tsugane S, et al. Changing trends in the prevalence of H. pylori infection in Japan (1908–2003): A systematic review and meta-

regression analysis of 170,752 individuals. Scientific Reports 2017;7. doi:10.1038/s41598-017-15490-7.

218. Wang M, Yuan Y, Wang Z, Liu D, Wang Z, Sun F, et al. Prevalence of orofacial clefts among live births in China: A systematic review and meta-analysis. Birth Defects Research 2017;109:1011–9. doi:10.1002/bdr2.1043.

219. Wang J, Wu X, Lai W, Long E, Zhang X, Li W, et al. Prevalence of depression and depressive symptoms among outpatients: A systematic review and meta-analysis. BMJ Open 2017;7:e017173. doi:10.1136/bmjopen-2017-017173.

220. Wang Z-D, Wang S-C, Liu H-H, Ma H-Y, Li Z-Y, Wei F, et al. Prevalence and burden of Toxoplasma gondii infection in HIV-infected people: A systematic review and meta-analysis. The Lancet HIV 2017;4:e177–88. doi:10.1016/s2352-3018(17)30005-x.

221. Williamson V, Stevelink SAM, Greenberg K, Greenberg N. Prevalence of mental health disorders in elderly U.S. military veterans: A meta-analysis and systematic review. The American Journal of Geriatric Psychiatry 2018;26:534–45. doi:10.1016/j.jagp.2017.11.001.

222. Wojtera M, Paradis J, Husein M, Nichols AC, Barrett JW, Salvadori MI, et al. The prevalence of human papillomavirus in pediatric tonsils: A systematic review of the literature. Journal of Otolaryngology - Head & Neck Surgery 2018;47. doi:10.1186/s40463-018-0255-1.

223. Wolke D, Bilgin A, Samara M. Systematic review and meta-analysis: fussing and crying durations and prevalence of colic in infants. The Journal of Pediatrics 2017;185:55–61.e4. doi:10.1016/j.jpeds.2017.02.020.

224. Wolpe RE, Zomkowski K, Silva FP, Queiroz APA, Sperandio FF. Prevalence of female sexual dysfunction in Brazil: A systematic review. European Journal of Obstetrics & Gynecology and Reproductive Biology 2017;211:26–32. doi:10.1016/j.ejogrb.2017.01.018.

225. Woody CA, Ferrari AJ, Siskind DJ, Whiteford HA, Harris MG. A systematic review and meta-regression of the prevalence and incidence of perinatal depression. Journal of Affective Disorders 2017;219:86–92. doi:10.1016/j.jad.2017.05.003.

226. Workneh MH, Bjune GA, Yimer SA. Prevalence and associated factors of tuberculosis and diabetes mellitus comorbidity: A systematic review. Plos One 2017;12:e0175925. doi:10.1371/journal.pone.0175925.

227. Wu H, Meng X, Wild SH, Gasevic D, Jackson CA. Socioeconomic status and prevalence of type 2 diabetes in mainland China, Hong Kong and Taiwan: a systematic review. Journal of Global Health 2017;7. doi:10.7189/jogh.07.011103.

228. Xiao W, Chen X, Yan W, Zhu Z, He M. Prevalence and risk factors of epiretinal membranes: A systematic review and meta-analysis of population-based studies. BMJ Open 2017;7:e014644. doi:10.1136/bmjopen-2016-014644.

229. Xie Y, Szeto G, Dai J. Prevalence and risk factors associated with musculoskeletal complaints among users of mobile handheld devices: A systematic review. Applied Ergonomics 2017;59:132–42. doi:10.1016/j.apergo.2016.08.020.

230. Xu Y, Chen X, Wang K. Global prevalence of hypertension among people living with HIV: a systematic review and meta-analysis. Journal of the American Society of Hypertension 2017;11:530–40. doi:10.1016/j.jash.2017.06.004.

231. Yang M, Zhao Y, Yin X, Chen Z, Yang C, Li L, et al. Prevalence, risk factors, and characteristics of the “adding-on” phenomenon in idiopathic scoliosis after correction surgery. Spine 2018;43:780–90. doi:10.1097/brs.0000000000002423.

232. Yao Z, Wang C, Zhang Q, Ma S, Gui B, Duan C. Prevalence of abdominal artery calcification in dialysis patients with end-stage renal disease: a systematic review and meta-analysis. International Urology and Nephrology 2017;49:2061–9. doi:10.1007/s11255-017-1685-9.

233. Zhang L, Fu T, Yin R, Zhang Q, Shen B. Prevalence of depression and anxiety in systemic lupus erythematosus: A systematic review and meta-analysis. BMC Psychiatry 2017;17. doi:10.1186/s12888-017-1234-1.

234. Zheng Y, Wu X, Lin X, Lin H. The prevalence of depression and depressive symptoms among eye disease patients: A systematic review and meta-analysis. Scientific Reports 2017;7. doi:10.1038/srep46453.

235. Zhou Y, Ke S-J, Qiu X-P, Liu L-B. Prevalence, risk factors, and prognosis of orthostatic hypotension in diabetic patients. Medicine 2017;96:e8004. doi:10.1097/md.0000000000008004.
