# Supplementary material for: How are systematic reviews of prevalence conducted? A methodological study
Source: BMC Med Res Methodol. 2020 Apr 26;20:96. doi: 10.1186/s12874-020-00975-3 (PMC7184711; doi:10.1186/s12874-020-00975-3)
Supplement: Supplementary file 2 — Additional file 2. List of excluded articles, with reasons for exclusion. [file 12874_2020_975_MOESM2_ESM.pdf]

Additional file 2: List of full text excluded with reasons

Author, year	Reason for exclusion
**Chapman, 2017 (1)**	Study assessed the association between variables, not prevalence
**Cooney, 2017 (2)**	Study assessed the association between variables, not prevalence
**Eusebi, 2018 (3)**	Study assessed the association between variables, not prevalence
**Fuccio, 2017 (4)**	Unit of measurement was not patients
**Goodarzi, 2017 (5)**	Study assessed the association between variables, not prevalence
**Goyal, 2017 (6)**	Unit of measurement was not patients
**Haeggblom, 2017 (7)**	Study assessed the association between variables, not prevalence
**Kurin, 2018 (8)**	Study assessed the association between variables, not prevalence
**Nokhodian, 2017 (9)**	Unit of measurement was not patients
**Pereira-Miranda, 2017 (10)**	Study assessed the association between variables, not prevalence
**Prioreschi, 2017 (11)**	Study assessed the association between variables, not prevalence
**Ross, 2017 (12)**	Study assessed the association between variables, not prevalence
**Verlaan, 2017 (13)**	Study assessed the association between variables, not prevalence
**Wang, 2017 (14)**	Full text not found
**Wang, 2017 (15)**	Study assessed the association between variables, not prevalence
**Ziukaite, 2017 (16)**	Study assessed the association between variables, not prevalence

References:

1. Chapman, C., Slade, T., Swift, W., Keyes, K., Tonks, Z. and Teesson, M. (2017). Evidence for Sex Convergence in Prevalence of Cannabis Use: A Systematic Review and Meta-Regression. Journal of Studies on Alcohol and Drugs, 78(3), pp.344-352.
2. Cooney, L., Lee, I., Sammel, M. and Dokras, A. (2017). High prevalence of moderate and severe depressive and anxiety symptoms in polycystic ovary syndrome: a systematic review and meta-analysis. Human Reproduction, 32(5), pp.1075-1091.
3. Eusebi, L., Ratnakumaran, R., Bazzoli, F. and Ford, A. (2018). Prevalence of Dyspepsia in Individuals With Gastroesophageal Reflux–Type Symptoms in the Community: A Systematic Review and Meta-analysis. Clinical Gastroenterology and Hepatology, 16(1), pp.39-48.e1.
4. Fuccio, L., Repici, A., Hassan, C., Ponchon, T., Bhandari, P., Jover, R., Triantafyllou, K., Mandolesi, D., Frazzoni, L., Bellisario, C., Bazzoli, F., Sharma, P., Rösch, T. and Rex, D. (2017). Why attempt en bloc resection of non-pedunculated colorectal adenomas? A systematic review of the prevalence of superficial submucosal invasive cancer after endoscopic submucosal dissection. Gut, 67(8), pp.1464-1474.
5. Mahvi, A., Goodarzi, F., Hosseini, M., Nodehi, R., Kharazifard, M. and Parvizishad, M. (2017). Prevalence of dental caries and fluoride concentration of drinking water: A systematic review. Dental Research Journal, 14(3), p.163.
6. Goyal, V., Kadam, V., Narang, P. and Singh, V. (2017). Prevalence of drug-resistant pulmonary tuberculosis in India: systematic review and meta-analysis. BMC Public Health, 17(1).
7. Haeggblom, L., Ramqvist, T., Tommasino, M., Dalianis, T. and Näsman, A. (2017). Time to change perspectives on HPV in oropharyngeal cancer. A systematic review of HPV prevalence per oropharyngeal sub-site the last 3 years. Papillomavirus Research, 4, pp.1-11.
8. Kurin, M., Bielefeldt, K. and Levinthal, D. (2018). Prevalence of Nausea and Vomiting in Adults Using Ropinirole: A Systematic Review and Meta-Analysis. Digestive Diseases and Sciences, 63(3), pp.687-693.
9. Ataei, B., Nokhodian, Z., Feizi, A., Hoseini, S. and Mostafavi, E. (2017). Epidemiology of Q fever in Iran: A systematic review and meta-analysis for estimating serological and molecular prevalence. Journal of Research in Medical Sciences, 22(1), p.121.
10. Pereira-Miranda, E., Costa, P., Queiroz, V., Pereira-Santos, M. and Santana, M. (2017). Overweight and Obesity Associated with Higher Depression Prevalence in Adults: A Systematic Review and Meta-Analysis. Journal of the American College of Nutrition, 36(3), pp.223-233.
11. Prioreschi, A., Munthali, R., Soepnel, L., Goldstein, J., Micklesfield, L., Aronoff, D. and Norris, S. (2017). Incidence and prevalence of type 2 diabetes mellitus with HIV infection in Africa: a systematic review and meta-analysis. BMJ Open, 7(3), p.e013953.
12. Ross, L., Salway, T., Tarasoff, L., MacKay, J., Hawkins, B. and Fehr, C. (2017). Prevalence of Depression and Anxiety Among Bisexual People Compared to Gay, Lesbian, and Heterosexual Individuals:A Systematic Review and Meta-Analysis. The Journal of Sex Research, 55(4-5), pp.435-456.

13. Verlaan, S., Ligthart-Melis, G., Wijers, S., Cederholm, T., Maier, A. and de van der Schueren, M. (2017). High Prevalence of Physical Frailty Among Community-Dwelling Malnourished Older Adults–A Systematic Review and Meta-Analysis. Journal of the American Medical Directors Association, 18(5), pp.374-382.
14. Goodarzi, F., Hossein Mahvi, A., Hosseini, M., Nabizadeh Nodehi, R., Javad Kharazifard, M. and Parvizishad, M. (2019). Prevalence of Bicuspid Aortic Valve in Chinese Patients with Aortic Valve Disease: A Systematic Review. 14(3), pp.163-8.
15. Wang, S., Chen, Y., Xu, X., Hu, W., Shen, H. and Chen, J. (2017). Prevalence of hepatitis B virus and hepatitis C virus infection in patients with systemic lupus erythematosus: a systematic review and meta-analysis. Oncotarget, 8(60).
16. Ziukaite, L., Slot, D. and Van der Weijden, F. (2018). Prevalence of diabetes mellitus in people clinically diagnosed with periodontitis: A systematic review and meta-analysis of epidemiologic studies. Journal of Clinical Periodontology, 45(6), pp.650-662.
