# Supplementary material for: How are systematic reviews of prevalence conducted? A methodological study
Source: BMC Med Res Methodol. 2020 Apr 26;20:96. doi: 10.1186/s12874-020-00975-3 (PMC7184711; doi:10.1186/s12874-020-00975-3)
Supplement: Supplementary file 3 — Additional file 3. Complete data extraction table. [file 12874_2020_975_MOESM3_ESM.pdf]

First author (last name)	Title	Number of authors	Funding	Number of studies included in the review	Search strategy avaliable (for at least one database)?	Databases (number)	Databases (description)	Reviewers	Protocol (registered or published)	Reporting guideline	Risk of bias of included studies	Reviewers	Instrument	Used it as a inclusion criteria?	Overall quality assessment	Methods
**Abd ElHafeez**	Prevalence and burden of chronic kidney disease among the general population and high-risk groups in Africa: a systematic review	6	Yes	152	Yes	2	Ovid MEDLINE<br>PubMed	Search: two reviewers<br>Title and abstract screening: two independent reviewers<br>Full text elegibility: two independent reviewers<br>Data extraction: one reviewer extracted, a second reviewer independently verified extraction	No	PRISMA	Yes	Two independent reviewers	New tool - judgement based on criteria described in Shamliyan, 2010; Juni, 2001; Whiting, 2003	No	No	NA
**Abuabara**	The prevalence of atopic dermatitis beyond childhood: a systematic review and meta-analysis of longitudinal studies	5	Yes	7	Yes	2	Embase<br>PubMed	Title and abstract screening: two independent reviewers<br>Full text elegibility: two independent reviewers<br>Data extraction: two independent reviewers	PROSPERO<br>CRD42016033553	PRISMA<br>MOOSE	Yes	Two independent reviewers	Newcastle-Ottawa Scale (Cohort studies)	No	No	NA
**Adeloye**	Estimating the prevalence, hospitalisation and mortality from type 2 diabetes mellitus in Nigeria: a systematic review and meta-analysis	7	No	42	Yes	4	Africa Journals Online (AJOL)<br>Embase<br>Global Health<br>Google Scholar<br>MEDLINE	Search: two independent reviewers<br>Study selection: two independent reviewers	No	MOOSE	Yes	NR	New tool- judgement based on criteria described in Stanifer, 2014; Pai, 2004; Guyatt, 2002; and Juni, 2001	Yes	No	NA
**Akbari**	High prevalence of hypertension among Iranian children and adolescents: a systematic review and meta-analysis	7	Yes	17	Incomplete	9	Google Scholar<br>Irandoc<br>Iranmedex<br>Magiran<br>PubMed<br>ScienceDirect<br>Scientific Information Database (SID)<br>Scopus<br>Web of Science	Search: two independent reviewers	No	No	Yes	NR	Tool from a previous systematic review of prevalence (Moosazadeh, 2013)	Yes	No	NA
**Akioyamen**	Estimating the prevalence of heterozygous familial hypercholesterolaemia: a systematic review and meta-analysis	7	Yes	21	Yes	6	Cochrane Library<br>Embase<br>Global Health<br>MEDLINE<br>PsycINFO<br>PubMed	Title and abstract screening: two independent reviewers<br>Full text elegibility: two independent reviewers<br>Data extraction: one reviewer extracted, a second reviewer independently verified extraction	PROSPERO<br>CRD42016042208	MOOSE	Yes	Two independent reviewers	Effective Public Health Practice Project (EPHPP) Quality Assessment Tool for Quantitative Studies	No	No	NA
**Al-Moraissi**	Do patients with malocclusion have a higher prevalence of temporomandibular disorders than controls both before and after orthognathic surgery? A systematic review and meta-analysis	3	No	6	Yes	3	Cochrane Central<br>Embase<br>PubMed	Full text elegibility: two reviewers<br>Data extraction: two independent reviewers	No	PRISMA	Yes	NR	New tool - criteria combined from MOOSE, STROBE and PRISMA	No	No	NA
**Alabdali**	A systematic review of the prevalence and types of adverse events in interfacility critical care transfers by paramedics	4	Yes	7	Yes	4	CINAHL<br>Embase<br>MEDLINE Ovid<br>Web of Science	Full text elegibility: two independent reviewers<br>Data extraction: two independent reviewers	No	PRISMA	Yes	NR	Unclear	No	No	NA
**Aleva**	Prevalence and localization of pulmonary embolism in unexplained acute exacerbations of COPD: a systematic review and meta-analysis	6	No	7	Incomplete	2	Embase<br>MEDLINE	Title screening: one reviewer<br>Abstract screening: two independent reviewers<br>Full text elegibility: two independent reviewers<br>Data extraction: two independent reviewers	No	PRISMA	Yes	Two independent reviewers	STROBE	No	No	NA
**Aliyu**	Prevalence of multidrug-resistant gram-negative bacteria among nursing home residents: a systematic review and meta-analysis	3	Yes	12	Incomplete	5	Cochrane Central Register of Clinical Trials<br>CINAHL<br>EBSCO<br>Ovid Medline<br>Web of Science	Title and abstract screening: one reviewer selected, a second reviewer independently verified selection<br>Full text elegibility: one reviewer selected, a second reviewer independently verified selection<br>Data extraction: two reviewers	No	MOOSE	Yes	Two independent reviewers	Downs and Black (modified)	No	No	NA
**Alleblas**	Prevalence of musculoskeletal disorders among surgeons performing minimally invasive surgery: a systematic review	6	NR	35	Yes	6	CINAHL<br>Cochrane<br>Embase<br>PSychINFO<br>PubMed<br>Web of Science	Title and abstract screening: two independent reviewers<br>Full text elegibility: two independent reviewers	No	PRISMA	Yes	NR	STROBE	No	Yes	Mean STROBE score of all studies
**Almousa**	The prevalence of urinary incontinence in nulliparous adolescent and middle-aged women and the associated risk factors: a systematic review	2	No	18	Incomplete	4	CINAHL (EBSCO)<br>Cochrane<br>Embase (Ovid)<br>Google Scholar<br>PubMed	Title and abstract screening: two independent reviewers<br>Full text elegibility: two independent reviewers<br>Data extraction: two independent reviewers	No	PRISMA	Yes	NR	Loney, 1998	No	No	NA
**Aluisio**	Prevalence of HIV-seropositivity and associated impact on mortality among injured patients from low-and middle-income countries: a systematic review and meta-analysis	7	No	16	Unclear	6	CINAHL<br>Cochrane<br>Embase<br>Global Health<br>POPLINE<br>Pubmed	Title and abstract screening: two independent reviewers<br>Data extraction: two reviewers	No	PRISMA	Yes	NR	GRADE (modified)	No	No	NA
**Anderson**	Prevalence and risk of mental disorders in the perinatal period among migrant women: a systematic review and meta-analysis	4	Yes	53	Yes	6	CINAHL<br>Cochrane Central<br>EMBASE<br>Maternal and Infant Care<br>MEDLINE<br>PsycINFO	Title and abstract screening: two independent reviewers<br>Full text elegibility: two independent reviewers<br>Data extraction: one reviewer	PROSPERO<br>CRD42015016262	PRISMA	Yes	Two independent reviewers	New tool adapted from Downs and Black, 1998; MacLehose, 2000; Trevillion, 2012; Wells, 2009; and CASP Checklist.	No	No	NA
**Andrews**	Chronic widespread pain prevalence in the general population: a systematic review	3	No	30	Yes	7	AMED<br>Cochrane<br>CINAHL<br>MEDLINE<br>OVID<br>PSYCinfo<br>PubMed	Title screening: one reviewer selected, a second reviewer verified selection<br>Abstract screening: two independent reviewers	No	No	Yes	Two independent reviewers	STROBE (modified)	No	No	NA
**Arabsalmani**	Is Herpes Simplex virus (HSV) a sign of encephalitis in Iranian newborns? Prevalence of HSV infection in pregnant women in Iran: a systematic review and meta-analysis	7	NR	5	Yes	7	Iranmedex<br>MagIran<br>Ovid<br>PubMed<br>Science Direct<br>Science Information Database (SID)<br>Scopus	Title and abstract screening: two independent reviewers	No	No	Yes	NR	STROBE	No	No	NA
**Azizpour**	Prevalence of low back pain in Iranian nurses: a systematic review and meta-analysis	4	Yes	22	Incomplete	11	CIVILICA<br>GoogleScholar<br>IMEMR: Index Medicus for Eastern Mediterranean Region<br>Irandoc: Iranian Research Institute for Information Science and Technology<br>Iran Medex: an Iranian Biomedical Journal<br>Magiran: an Iranian Journal Database<br>PubMed<br>Science Direct<br>Scopus<br>SID: Scientific Information Database<br>Web of Science	Data extraction: two independent reviewers	No	PRISMA	Yes	Two independent reviewers	Tool from a previous systematic review of prevalence (Walker, 2000)	Yes	No	NA
**Badawi**	Prevalence of chronic comorbidities in chikungunya: a systematic review and meta-analysis	4	NR	11	Incomplete	4	Embase<br>Embase Classic<br>MEDLINE Ovid<br>PubMed	Title and abstract screening: two independent reviewers	No	No	No	NA	NA	NA	No	NA
**Beckwee**	Prevalence of aromatase inhibitor-induced arthralgia in breast cancer: a systematic review and meta-analysis	4	No	21	Yes	3	Cochrane<br>PubMed<br>Web of Science	Title and abstract screening: two independent reviewers<br>Full text elegibility: one reviewer<br>Data extraction: one reviewer	No	PRISMA	Yes	NR	STROBE	No	No	NA
**Bernard**	Prevalence and factors associated with depression in people living with HIV in sub-Saharan Africa: a systematic review and meta-analysis	3	Yes	66	Yes	7	African Index Medicus<br>African journal online<br>Medline<br>PsycArticles<br>PsycInfo<br>Psychology & Behavioral Science Collection<br>Scopus	NR	No	PRISMA	No	NA	NA	NA	Yes	GRADE
**Bi-Mohammed**	Prescription opioid abuse in prison settings: a systematic review of prevalence, practice and treatment responses	5	No	10	Yes	8	ASSIA<br>CINAHL<br>Cochrane Library<br>Embase<br>Google Scholar<br>MEDLINE<br>PsychINFO<br>Science Direct	Title and abstract screening: two independent reviewers<br>Full text elegibility: one reviewer	No	No	Yes	NR	New tool based on the National Institute for Health and Care Excellence (NICE) guidelines manual and the Cochrane Handbook for Systematic Reviews	No	No	NA
**Bigna**	Prevalence of prediabetes and diabetes mellitus among adults residing in Cameroon: a systematic review and meta-analysis	4	No	12	Yes	4	African Index Medicus<br>African Journals Online<br>Excerpta Medica Database<br>PubMed	Title and abstract screening: two independent reviewers<br>Full text elegibility: two independent reviewers<br>Data extraction: one reviewer selected, a second reviewer independently verified selection	PROSPERO<br>CRD42017070431	PRISMA	Yes	Two independent reviewers	Hoy, 2012	No	No	NA
**Bigna (A)**	Prevalence of chronic obstructive pulmonary disease in the global population with HIV: a systematic review and meta-analysis	4	No	30	Yes	4	Embase<br>Global Index Medicus<br>PubMed<br>Web of Science	Title and abstract screening: two independent reviewers<br>Full text elegibility: two independent reviewers<br>Data extraction: two independent reviewers	PROSPERO<br>CRD42016052639<br>Published protocol	No	Yes	Two independent reviewers	Hoy, 2012	No	No	NA
**Bigna (B)**	Prevalence and etiologies of pulmonary hypertension in Africa: a systematic review and meta-analysis	4	No	25	Yes	4	African Index Medicus<br>African Journals Online<br>Excerpta Medica Database<br>PubMed	Title and abstract screening: two independent reviewers<br>Full text elegibility: two independent reviewers<br>Data extraction: two independent reviewers	PROSPERO<br>CRD42016049351<br>Published protocol	MOOSE	Yes	Two independent reviewers	Newcastle-Ottawa Scale	No	No	NA
**Bijlard**	A systematic review on the prevalence, etiology, and pathophysiology of intrinsic pain in dermal scar tissue	6	No	52	Yes	6	CINAHL<br>Cochrane central<br>Embase<br>Medline<br>PubMed<br>Web of nScience	Title and abstract screening: two independent reviewers<br>Full text elegibility: two reviewers<br>Data extraction: two independent reviewers	No	No	No	NA	NA	NA	No	NA
**Bitta**	Antimalarial drugs and the prevalence of mental and neurological manifestations: a systematic review and meta-analysis (version 2)	6	Yes	51	Yes	10	Canadian Agency for Drugs and Technologies in Health<br>Central<br>CINAHL<br>Directory of Open-Access Repository<br>EMBASE<br>MEDLINE<br>Open Grey Database<br>PsycINFO<br>Web of Science<br>World Cat database	Elegibility assessment: two independent reviewers	No	PRISMA	Yes	NR	JBI Prevalence Critical Appraisal Tool	Yes	No	NA

First author (last name)	Metanalysis	Model	Variance estimator	Transformation	Subgroup analysis	Meta-regression	Prediction / predictive interval	Heterogeneity	Publication bias	Software
**Abd ElHafeez**	No	NA	NA	NA	NA	NA	NA	NA	NA	NA
**Abuabara**	No	NA	NA	NA	NA	NA	NA	NA	NA	NA
**Adeloye**	Yes	Random-effects	DerSimonian and Laird	NR	Yes	Yes	No	I^2	Funnel plot	STATA
**Akbari**	Yes	Random-effects	NR	NR	Yes	Yes	No	Cochran's Q<br>I^2<br>Sensitivity analysis	No	STATA
**Akioyamen**	Yes	Random-effects	DerSimonian and Laird	Double arcsine	Yes	Yes	No	I^2<br>Sensitivity analysis	Begg's test<br>Doi plot<br>Egger's test<br>Funnel plot<br>Trim and fill	MetaXL<br>STATA
**Al-Moraissi**	Yes	Random-effects	NR	NR	No	No	No	Cochran's Q<br>I2	No	Comprehensive Meta-Analysis
**Alabdali**	No	NA	NA	NA	NA	NA	NA	NA	NA	NA
**Aleva**	Yes	Random-effects	NR	Double arcsine	No	Yes	No	Cochran's Q<br>I^2	No	R
**Aliyu**	Yes	Random-effects	NR	NR	Yes	No	No	Cochran's Q<br>I2<br>Sensitivity analysis	Rosenthal's fail-safe N test	Comprehensive Meta-Analysis
**Alleblas**	Yes	Random-effects	Hartung-Knapp-Sidik-Jonkman	Arcsine	No	No	Yes	I^2	No	R
**Almousa**	No	NA	NA	NA	NA	NA	NA	NA	NA	NA
**Aluisio**	Yes	Random-effects	NR	NR	No	No	No	Chi-square	No	STATA
**Anderson**	Yes	Random-effects	NR	NR	No	No	No	I^2<br>Sensitivity analysis	No	STATA
**Andrews**	Yes	Random-effects	NR	NR	Yes	Yes	No	I^2	No	R
**Arabsalmani**	Yes	Random-effects	NR	NR	Yes	No	No	Cochran's Q<br>I^2	No	Review Manager
**Azizpour**	Yes	Random-effects	NR	NR	Yes	Yes	No	Cochran's Q<br>I^2	Begg's test<br>Funnel plot	STATA
**Badawi**	Yes	Random-effects	NR	Raw	No	No	No	Cochran's Q<br>I^2	Egger's test<br>Funnel plot	Comprehensive Meta-Analysis
**Beckwee**	Yes	Random-effects	NR	NR	Yes	No	No	I^2<br>Sensitivity analysis	No	Open MetaAnalyst
**Bernard**	Yes	NR	NR	NR	No	No	No	I^2	No	MetaXL
**Bi-Mohammed**	No	NA	NA	NA	NA	NA	NA	NA	NA	NA
**Bigna**	Yes	Random-effects	NR	Freeman–Tukey double arcsine	Yes	Yes	No	Cochran's Q<br>I^2	Egger's test<br>Funnel plot<br>Trim and fill	STATA
**Bigna (A)**	Yes	Random-effects	NR	Freeman–Tukey double arcsine	Yes	Yes	No	I^2<br>Sensitivity analysis	Egger's test<br>Funnel plot	R
**Bigna (B)**	Yes	Random-effects	NR	Freeman–Tukey double arcsine	No	No	No	Cochran's Q<br>I^2	Egger's test	STATA
**Bijlard**	No	NA	NA	NA	NA	NA	NA	NA	NA	NA
**Bitta**	Yes	Random-effects	NR	NR	No	Yes	No	Cochran's Q<br>I^2	Funnel plot	STATA

**Bourne**	Magnitude, temporal trends, and projections of the global prevalence of blindness and distance and near vision impairment: a systematic review and meta-analysis	18	Yes	61	Yes	3	Embase<br>Medline<br>Wholis	NR	No	PRISMA	No	NA	NA	NA	No	NA	Yes	NR	NR	NR	No	No	No	NR	No	NR
**Brinjikji**	Prevalence and characteristics of brain arteriovenous malformations in hereditary hemorrhagic telangiectasia: a systematic review and meta-analysis	4	NR	39	Yes	4	Embase<br>Medline<br>Scopus<br>Web of Science	Data extraction: one reviewer	No	PRISMA	Yes	NR	Newcastle-Ottawa Scale (modified)	No	No	NA	Yes	Random-effects	NR	NR	No	No	No	Cochran's Q<br>I^2	No	Open MetaAnalyst
**Cagnazzo**	Intracranial aneurysms in patients with autosomal dominant polycystic kidney disease: prevalence, risk of rupture, and management. A systematic review	4	No	16	Yes	3	Embase Ovid<br>MEDLINE Ovid<br>PubMed	Elegibility assessment: two independent reviewers	No	PRISMA	Yes	Two independent reviewers	Newcastle-Ottawa Scale (modified)	No	No	NA	No	NA	NA	NA	NA	NA	NA	NA	NA	NA
**Calado**	Prevalence of Adolescent Problem Gambling: A Systematic Review of Recent Research	3	NR	44	Incomplete	7	Google Scholar<br>PsycARTICLES<br>PsycINFO<br>PubMED<br>Science Direct<br>Scopus<br>Wiley Online Library	NR	No	No	No	NA	NA	NA	No	NA	No	NA	NA	NA	NA	NA	NA	NA	NA	NA
**Casavechia**	Systematic review and meta-analysis on Schistosoma mansoni infection prevalence, and associated risk factors in Brazil	7	No	27	Yes	3	LILACS<br>PubMed<br>Web of Science	Data extraction: two reviewers	No	PRISMA	Yes	NR	STROBE	No	No	NA	Yes	Random-effects	DerSimonian and Laird	NR	Yes	No	No	Cochran's Q<br>I^2	Begg's test<br>Egger's test<br>Funnel plot	STATA
**Chaponda**	Systematic review of the prevalence of psychiatric illness and sleep disturbance as co-morbidities of HIV infection in the UK	6	Yes	16	Yes	4	Cochrane<br>Embase Ovid<br>MEDLINE In process & Other Non-Indexed Citations (Ovid)<br>MEDLINE (Ovid)	Title and abstract screening: one reviewer<br>Full text elegibility: two reviewers<br>Data extraction: one reviewer extracted, a second reviewer verified extraction	No	PRISMA	No	NA	NA	NA	No	NA	No	NA	NA	NA	NA	NA	NA	NA	NA	NA
**Chen**	Prevalence of restless legs syndrome during pregnancy: a systematic review and meta-analysis	10	Yes	27	Yes	3	Embase<br>PubMed<br>Web of Science	Elegibility assessment: two independent reviewers<br>Data extraction: two independent reviewers	No	PRISMA	No	NA	NA	NA	No	NA	Yes	Random-effects	NR	NR	Yes	No	No	I^2<br>Sensitivity analysis	Begg's test<br>Funnel plot	STATA
**Chernet**	Prevalence rates of six selected infectious diseases among African migrants and refugees: a systematic review and meta-analysis	7	No	113	Incomplete	3	Embase<br>PubMed<br>Web of Science	Elegibility assessment: two independent reviewers	No	PRISMA	Yes	NR	GRADE (modified)	No	No	NA	Yes	Random-effects	NR	NR	Yes	No	No	I^2	No	STATA
**Cheungpasitporn**	Decline in prevalence and risk of helicobacter pylori in kidney transplant recipients: a systematic review and meta-analysis	10	NR	11	Yes	3	Cochrane<br>Embase<br>Medline	Title and abstract screening: two independent reviewers	PROSPERO CRD42016051408	PRISMA	Yes	Two independent reviewers	Tool from a previous systematic review of prevalence (Herzog, 2013)	No	No	NA	Yes	Random-effects	DerSimonian and Laird	NR	Yes	No	No	Cochran's Q<br>I^2	No	MetaXL
**Coelho**	Microcephaly Prevalence in Infants Born to Zika Virus-Infected Women: A Systematic Review and Meta-Analysis	2	Yes	8	Incomplete	2	Cochrane<br>PubMed	Data extraction: two independent reviewers	No	No	Yes	Two independent reviewers	CASP (cohort study)	No	No	NA	Yes	Random-effects	NR	Log	No	No	No	Cochran's Q<br>I^2	No	R
**Dahlgren**	Feeding and eating disorders in the DSM-5 era: a systematic review of prevalence rates in non-clinical male and female samples	3	Yes	19	Yes	1	PubMed	Titles screening: two reviewers<br>Abstract screening: two reviewers<br>Full text elegibility: two reviewers	No	PRISMA	No	NA	NA	NA	No	NA	No	NA	NA	NA	NA	NA	NA	NA	NA	NA
**Dalvand**	Prevalence of metabolic syndrome among Iranian population: a systematic review and meta-analysis	9	NR	34	Incomplete	10	Google scholar<br>IranDoc<br>Iran Medex<br>Magiran<br>Medlib<br>PubMed<br>Science Direct<br>SID (Scientific Information Database)<br>Scopus<br>Web of Science	Elegibility assessment: two independent reviewers	No	PRISMA	No	NA	NA	NA	No	NA	Yes	Random-effects	DerSimonian and Laird	NR	Yes	Yes	No	Cochran's Q<br>I^2	Egger's test<br>Funnel plot	STATA
**Degenhardt**	Global prevalence of injecting drug use and sociodemographic characteristics and prevalence of HIV, HBV, and HCV in people who inject drugs: a multistage systematic review	15	Yes	1147	Yes	3	Embase<br>MEDLINE<br>PsycINFO	Title and abstract screening: one reviewer screened, a second reviewer verified a sample<br>Full text elegibility: two independent reviewers<br>Data extractiuon: one reviewer extracted, a second reviewer verified extraction	PROSPERO CRD42016052858 and CRD42016052853	PRISMA<br>GATHER	Yes	NR	NR	No	No	NA	Yes	Random-effects	NR	NR	No	No	No	NR	No	STATA
**Dekel**	Childbirth induced posttraumatic stress syndrome: a systematic review of prevalence and risk factors	3	Yes	36	Incomplete	2	PsychInfo<br>PubMed	NR	No	MOOSE	Yes	Two reviewers	Tool from a previous systematic review of prevalence (Andersen, 2012) (modified)	No	No	NA	No	NA	NA	NA	NA	NA	NA	NA	NA	NA
**Denfeld**	The prevalence of frailty in heart failure: a systematic review and meta-analysis	6	Yes	26	Incomplete	2	CINAHL<br>Pubmed	Elegibility assessment: two independent reviewers<br>Data extraction: one reviewer extracted, a second reviewer verified extraction	No	PRISMA<br>MOOSE	No	NA	NA	NA	No	NA	Yes	Random-effects	NR	Freeman–Tukey double arcsine	Yes	Yes	No	Cochran's Q<br>I^2	Egger's test<br>Funnel plot	Comprehensive Meta-Analysis<br>STATA
**Deng**	Prevalence of lipohypertrophy in insulin-treated diabetes patients: a systematic review and meta-analysis	5	NR	26	Yes	4	Cochrane<br>Embase<br>PubMed<br>Scopus	Search: two independent reviewers<br>Title and abstract screening: two independent reviewers<br>Data extraction: two independent reviewers	PROSPERO CRD42017065540	PRISMA	Yes	NR	Newcastle-Ottawa Scale (modified)	No	No	NA	Yes	Random-effects	NR	NR	Yes	No	No	I^2<br>Sensitivity analysis	Egger's test<br>Funnel plot	STATA
**Dennis**	Prevalence of antenatal and postnatal anxiety: systematic review and meta-analysis	3	Yes	102	Yes	8	CINAHL<br>Embase<br>Google Scholar<br>MEDLINE<br>PsycINFO<br>ResearchGate<br>Scopus<br>Web of Science	NR	No	PRISMA	Yes	Two independent reviewers	Effective Public Health Practice Project (EPHPP) Quality Assessment Tool for Observational Studies (modified)	No	No	NA	Yes	Random-effects	NR	NR	Yes	Yes	No	I^2	No	STATA
**Desikan**	Prevalence of hepatitis B and hepatitis C virus co-infection in India: a systematic review and meta-analysis	2	No	30	Incomplete	5	Cochrane Library<br>Google Scholar<br>Indian and International journals online<br>Medline<br>PubMed	Data extraction: one reviewer extracted, a second reviewer verified extraction	No	PRISMA	Yes	NR	Downs and Black (modified)	No	No	NA	Yes	Random-effects	DerSimonian and Laird	NR	Yes	No	No	Cochran's Q<br>I^2	Egger's test<br>Funnel plot<br>Harbord statistics	StatsDirect
**Dillon**	A systematic review describing incidence rate and prevalence of dysvascular partial foot amputation; how both have changed over time and compare to transtibial amputation	3	Yes	25	Yes	5	AMED<br>CINAHL<br>Embase<br>MEDLINE<br>ProQuest Nursing and Allied Health	Title and abstract screening: one reviewer<br>Full text elegibility: two independent reviewers<br>Data extraction: one reviewer extracted, a second reviewer verified extraction	PROSPERO CRD4201502918	PRISMA	Yes	Two independent reviewers	Critical Review Form – Quantitative Studies (Law)	No	No	NA	No	NA	NA	NA	NA	NA	NA	NA	NA	NA
**Ding**	The prevalence of polycystic ovary syndrome in reproductive-aged women of different ethnicity: a systematic review and meta-analysis	6	No	42	Yes	4	CINAHL<br>Cochrane<br>Embase<br>PubMed	Study screening and selection: three independent reviewers<br>Data extraction: two independent reviewers	No	No	Yes	Two independent reviewers	JBI Prevalence Critical Appraisal Tool and Newcastle-Ottawwa (modified)	No	No	NA	Yes	Random-effects	NR	NR	No	No	No	NR	No	JAGS<br>R
**Downs**	The prevalence of mental health disorders and symptoms in children and adolescents with cerebral palsy: a systematic review and meta-analysis	10	Yes	8	Yes	2	Medline<br>PsycINFO	Title and abstract screening: two independent reviewers<br>Data extraction: four independent reviewers	No	PRISMA	Yes	NR	JBI Prevalence Critical Appraisal Tool	No	No	NA	Yes	Random-effects	NR	NR	No	No	No	I^2	No	STATA
**Dyer**	Prevalence and characteristics of overweight and obesity in indigenous Australian children: a systematic review	6	Yes	22	Yes	7	Cochrane<br>Database of Abstracts of Reviews of Effects<br>EMBASE<br>Johanna Briggs Institute library<br>MEDLINE<br>Scopus<br>NHS Economic Evaluation Database	Title and abstract screening: one reviewer (second reviewer screened only a sample)<br>Full text elegibility: two independent reviewers<br>Data extraction: one reviewer extracted, a second reviewer verified extraction	PROSPERO CRD42014007626	No	Yes	Two independent reviewers	JBI Prevalence Critical Appraisal Tool (modified)	No	No	NA	No	NA	NA	NA	NA	NA	NA	NA	NA	NA
**Edwards**	Prevalence of low back pain in emergency settings: a systematic review and meta-analysis	5	Yes	19	Yes	2	Embase<br>Pubmed	Title and abstract screening: two independent reviewers<br>Full text elegibility: two independent reviewers<br>Data extraction: two independent reviewers	No	No	Yes	Two independent reviewers	Hoy, 2012 (modified)	No	Yes	GRADE (adaptation)<br>Domains: study limitations (overall risk of bias of the evidence identified), imprecision (study sample sizes), indirectness (generalizability of included studies) and inconsistency (unexplained heterogeneity)	Yes	Random-effects	Hartung-Knapp-Sidik-Jonkman	Double arcsine	Yes	Yes	No	Cochran's Q<br>I^2<br>Sensitivity analysis	No	STATA
**Ejike**	Prevalence of hypertension in Nigerian children and adolescents: a systematic review and trend analysis of data from the past four decades	1	NR	21	Incomplete	4	AJOL<br>Google Scholar<br>Medline/PubMed<br>Scopus	NR	No	No	No	NA	NA	NA	No	NA	Yes	NR	NR	NR	Yes	No	No	NR	No	NR
**Elbarazi**	Prevalence of and factors associated with burnout among health care professionals in Arab countries: a systematic review	4	No	19	Yes	6	Informit<br>MEDLINE<br>ProQuest<br>Psych INFO<br>PubMed<br>Science Direct	Title and abstract screening: two independent reviewers	No	PRISMA	Yes	Three independent reviewers	STROBE (modified)	No	No	NA	No	NA	NA	NA	NA	NA	NA	NA	NA	NA
**Elshahidi**	Prevalence of dementia in Egypt: a systematic review	5	NR	6	Yes	6	EBSCOhost<br>Google Scholar<br>Medline (Ovid)<br>PubMed<br>Scopus<br>Web of Science	Title and abstract screening: two independent reviewers<br>Full text elegibility: two independent reviewers<br>Data extraction: two independent reviewers	PROSPERO CRD42016039695	PRISMA	Yes	Two independent reviewers	Tool from a previous systematic review of prevalence (Mata, 2015)	No	No	NA	No	NA	NA	NA	NA	NA	NA	NA	NA	NA
**Emaneini (A)**	Prevalence of Methicillin-Resistant Staphylococcus aureus isolated from burn patients: A systematic review and meta-analysis	7	Yes	13	Incomplete	4	Embase<br>IranianDatabase<br>PubMed (Medline)<br>Web of Science	Title and abstract screening: two independent reviewers<br>Data extraction: two independent reviewers extracted, a third reviewer verified extraction	No	PRISMA	Yes	Two independent reviewers	JBI Prevalence Critical Appraisal Tool	Yes	No	NA	Yes	Random-effects	NR	NR	No	No	No	Cochran's Q<br>I^2	Begg's test<br>Egger's test<br>Funnel plot	Comprehensive Meta-Analysis
**Emaneini (B)**	Prevalence of Group B Streptococcus in Pregnant Women in Iran: A Systematic Review and Meta-analysis	4	No	25	Incomplete	4	Embase<br>Iranian database<br>Pubmed<br>Web of Science	Title and abstract screening: two independent reviewers<br>Data extraction: two independent reviewers extracted, a third reviewer verified extraction	No	PRISMA	Yes	Two independent reviewers	JBI Prevalence Critical Appraisal Tool	No	No	NA	Yes	Random-effects	NR	NR	Yes	No	No	Cochran's Q<br>I2<br>t^2 statistic	Begg's test<br>Egger's test<br>Funnel plot	Comprehensive Meta-Analysis
**Epstein**	Prevalence of work-related musculoskeletal disorders among surgeons and interventionalists: a systematic review and meta-analysis	7	Yes	126	Yes	4	Embase<br>MEDLINE(Ovid)<br>Web of Science<br>PubMed	Study selection: two independent reviewers<br>Data extraction: two independent reviewers	No	MOOSE	Yes	Two independent reviewers	JBI Prevalence Critical Appraisal Tool	Yes	No	NA	Yes	Random-effects	NR	NR	Yes	No	No	I^2<br>Sensitivity analysis	No	R
**Escobar-Morreale**	Prevalence of 'obesity-associated gonadal dysfunction' in severely obese men and women and its resolution after bariatric surgery: a systematic review and meta-analysis	4	Yes	29	Yes	2	Embase<br>PubMed	Study selection: three independent reviewers	No	PRISMA<br>MOOSE	Yes	NR	Q-index	No	No	NA	Yes	Quality effects	NR	Double arcsine	No	No	No	Cochran's Q<br>I^2<br>Sensitivity analysis	Doi plot<br>Funnel plot<br>LFK index	MetaXL
**Fan**	Prevalence of abnormally invasive placenta among deliveries in mainland China: A PRISMA-compliant systematic review and meta-analysis	11	NR	23	Incomplete	8	Chinese Biological Medical Literature database (CBM)<br>Chinese National Knowledge Infrastructure database (CNKI)<br>Chinese VIP database<br>Chinese WanFang Database<br>Cochrane Library<br>Elsevier<br>PubMed<br>Science Direct	Data extraction: two independent reviewers	PROSPERO CRD42016035833	PRISMA<br>MOOSE	Yes	Two independent reviewers	STROBE (modified)	No	No	NA	Yes	Random-effects	DerSimonian and Laird	Freeman–Tukey double arcsine	Yes	Yes	No	Cochran's Q<br>I^2	Begg's test<br>Egger's test<br>Funnel plot	STATA

**Fang**	Prevalence of Kaposi's sarcoma-associated herpesvirus among intravenous drug users: a systematic review and meta-analysis	5	Yes	22	Yes	6	Chinese Biomedical Literature Database (CBM)<br>China National Knowledge Infrastructure Database (CNKI)<br>Embase<br>Medline (PubMed)<br>Wanfang Database<br>Web of Science	Title screening: two independent reviewers<br>Abstracts and full text: at least three reviewers<br>Data extraction: two independent reviewers
**Farahani**	Prevalence and distribution of non-AIDS causes of death among HIV-infected individuals receiving antiretroviral therapy: a systematic review and meta-analysis	4	No	19	Incomplete	3	Embase<br>GoogleScholar<br>PubMed	Study screening: two revieweres
**Farrag**	A systematic review of childhood obesity in the Middle East and North Africa (MENA) region: Prevalence and risk factors meta-analysis	3	Yes	56	Incomplete	1	PubMed	Titles and abstract screening: two independent reviewers
**Fishbain**	Chronic pain types differ in ther reported prevalence of post-traumatic stress disorder (PTSD) and there is consistent evidance that chronic pain is associated with PTSD: an evidence-based structured systematic review	4	No	40	Incomplete	6	Amed<br>Embase<br>Medline<br>National Library of Medicine Physician Data Query<br>Psychological Abstracts<br>Science Citation Index	Titles and abstract screening: two independent reviewers<br>Full-text elegibility: one reviewer screened, a second reviewer verified<br>Data extraction: one reviewer extracted, a second reviewer verified extraction
**Fleming**	The prevalence of anxiety in patients with psoriasis: a systematic review of observational studies and clinical trials	6	No	15	Incomplete	3	Cochrane<br>Embase<br>PubMed	Title and abstract screening: two reviewers<br>Full text elegibility: two independent reviewers<br>Data extraction: one reviewer extracted, two reviewers verified extraction
**Foroutan**	A systematic review and meta-analysis of the prevalence of Leishmania infection in blood donors	7	Yes	20	No	6	CINAHL<br>Embase<br>PubMed<br>Science Direct<br>Scopus<br>Web of Sciences	Study selection: two reviewers
**Frayman**	A systematic review of the prevalence and impact of urinary incontinence in cystic fibrosis	3	Yes	12	Incomplete	3	Embase<br>Medline<br>PubMed	Data extraction: two independent reviewers
**French**	Prevalence of neuropathic pain in knee or hip osteoarthritis: a systematic review and meta-analysis	3	NR	9	Yes	6	CINAHL<br>Embase<br>GoogleScholar<br>PsychInfo<br>PubMed<br>Web of Science	Titles and abstract screening: two independent reviewers<br>Full-text elegibility: two independent reviewers<br>Data extraction: two independent reviewers
**Fu**	The prevalence of depression in rheumatoid arthritis in China: a systematic review	7	Yes	21	Incomplete	7	Chinese biomedical database (CBM)<br>Chinese journal full-text database (CNKI)<br>Chinese science and technology periodical database<br>EMBASE<br>GoogleScholar<br>Medline<br>Wanfang database	Data extraction: two independent reviewers
**Fuglkjaer**	Prevalence and incidence of musculoskeletal extremity complaints in children and adolescents: a systematic review	3	No	22	Yes	2	Embase<br>Medline	Titles and abstract screening: two reviewers<br>Full-text elegibility: two reviewers<br>Data extraction: two independent reviewers
**Gane**	Prevalence, incidence, and risk factors for shoulder and neck dysfunction after neck dissection: a systematic review	7	No	75	Yes	4	CINAHL<br>Cochrane<br>Embase<br>PubMed	Titles and abstract screening: two independent reviewers<br>Full-text elegibility: two independent reviewers<br>Data extraction: two independent reviewers
**Gerridzen**	Prevalence and severity of behavioural symptoms in patients with Korsakoff syndrome and other alcohol-related cognitive disorders: a systematic review	8	Yes	15	Yes	4	CINAHL<br>Embase<br>PsycInfo<br>PubMed	Titles and abstract screening: two independent reviewers<br>Full-text elegibility: two independent reviewers<br>Data extraction: two independent reviewers
**Ghaemmohamadi**	Prevalence of depression in cardiovascular patients in Iran: a systematic review and meta-analysis from 2000 to 2017	9	Yes	12	Yes	9	CINAHL<br>Embase<br>ISI Web of Science<br>Irandoc<br>MagIran<br>PsycINFO<br>PubMed/Medline<br>Scopus<br>SID	Search: two independent reviewers<br>Data extraction: two independent reviewers
**Gheshlagh**	The prevalence of restless syndrome in patients undergoing hemodialysis: a systematic review and meta-analysis study	6	Yes	26	Incomplete	8	Google Scholar<br>IranMedex<br>MagIran<br>PreQuest<br>PubMed<br>ScienceDirect<br>Scopus<br>SID	Titles and abstracts screening: two independent reviewers
**Ghorbani**	Prevalence of hepatitis C infection in Iranian hemodialysis patients: an updated systematic review and meta-analysis	13	Yes	54	No	7	Institute of Scientific Information<br>Iran-doc<br>Iran-Medex<br>NLM Gateway (for MEDLINE)<br>PubMed<br>Scientific Information Database<br>SCOPUS	Search: two independent reviewers<br>Data extraction: two independent reviewers
**Gilheaney**	The prevalence of oropharyngeal dysphagia in adults presenting with temporomandibular disorders associated with rheumatoid arthritis: a systematic review and meta-analysis	7	NR	19	Yes	9	Cochrane<br>CINAHL<br>EBSCO AMED<br>Embase<br>GoogleScholar<br>PubMed<br>Science Direct<br>Scopus<br>Web of Science	Titles and abstract screening: two independent reviewers (three reviewers, each one screened 2/3 of references)<br>Data extraction: three reviewers
**Gilheaney**	The prevalence of oral stage dysphagia in adults presenting with temporomandibular disorders: a systematic review and meta-analysis	8	NR	20	Yes	9	AMED<br>CINAHL<br>The Cochrane Database of Systematic Review<br>EMBASE<br>GoogleScholar<br>Science Direct<br>Scopus<br>PubMed<br>Web of Science	Titles and abstract screening: two independent reviewers<br>Full text eligibility: two independent reviewers<br>Data extraction: six independent reviewers
**Hackett**	An investigation into the prevalence of sleep disturbances in primary Sjogren's syndrome: a systematic review of the literature	8	Yes	8	Yes	5	Embase (Ovid)<br>Medline (Ovid)<br>PsychINFO (Ovid)<br>PubMed<br>Web of Science	Titles and abstract screening: two independent reviewers<br>Full text eligibility: two independent reviewers<br>Data extraction: one reviewer extracted, a second reviewer verified extraction
**Harirchian**	Worldwide prevalence of familial multiple sclerosis: a systematic review and meta-analysis	5	Yes	17	Incomplete	4	GoogleScholar<br>PubMed<br>Scopus<br>Web of Science	Search: two independent reviewers
**Harris**	Prevalence of clinically significant liver disease within the general population, as defined by non-invasive markers of liver fibrosis: a systematic review	5	Yes	19	Unclear	3	Embase<br>Medline<br>Web of Science	Search: two independent reviewers<br>Data extraction: two independent reviewers
**Harrisson**	Neuropathic pain in low back-related leg pain patients: what is the evidence of prevalence, characteristicsm and prognosis in primary care? A systematic review of the literature	5	Yes	12	Yes	7	AMED<br>CINAHL<br>Embase<br>GoogleScholar<br>MEDLINE<br>TRIP<br>Web of Science Core Collection	Title screening: one reviewer<br>Abstract screening: two independent reviewers<br>Data extraction: two independent reviewers
**Hart**	The prevalence of radiographic and MRI-defined patellofemoral osteoarthritis and structural pathology: a systematic review and meta-analysis	5	NR	85	Yes	6	CINAHL<br>EMBASE (OVID)<br>MEDLINE (OVID)<br>Scopus<br>SPORTDiscus<br>Web of Science	Title screening: two reviewers
**Harvey**	Prevalence, cause and diagnosis of lactose intolerance in children aged 1-5 years: a systematic review of 1995-2015 literature	8	NR	20	Yes	3	CAB Abstract<br>Embase<br>Medline	Title and abstract screening: EPPI Reviewer (1st stage) and three independent reviewers (2nd stage)<br>Full text elegibility: two reviewers<br>Data extraction: two independent reviewers
**He**	Prevalence of primary cardiac tumor malignancies in retrospective studies over six decades: a systematic review and meta-analysis	10	NR	38	Incomplete	3	Embase<br>PubMed<br>Web of Science	Study selection: two independent reviewers<br>Data extraction: two independent reviewers
**Heidari**	Prevalence of fibromyalgia in general population and patients, a systematic review and meta-analysis	3	Yes	65	Yes	7	Cochrane library databanks<br>Ebsco<br>Google scholar<br>Ovid<br>PubMed<br>Scopus<br>Web of Sciences	Search: two independent reviewers<br>Titles and abstract screening: two independent reviewers<br>Full text elegibility: two independent reviewers
**Heriseanu**	Grazing in adults with obesity and eating disorders: a systematic review of associated clinical features and meta-analysis of prevalence	4	No	38	Yes	6	CINAHL<br>Embase<br>GoogleScholar<br>Medline<br>PsycINFO<br>Web of Science	Title and abstract screening: one reviwer, a second reviewer screened a sample<br>Full text elegibility: one reviwer, a second reviewer screened a sample
**Hofstraat**	Current prevalence of chronic hepatitis B and C virus infection in the general population, blood donors and pregnant women in the EU/EEA: a systematic review	7	Yes	80	Yes	3	Cochrane Library<br>Embase<br>PubMed	Titles and abstract screening: two independent reviewers<br>Full text elegibility: more than one reviewer

**Fang**	No	PRISMA	No	NA	NA	NA	No	NA
**Farahani**	No	PRISMA	No	NA	NA	NA	No	NA
**Farrag**	No	No	No	NA	NA	NA	No	NA
**Fishbain**	No	No	Yes	Two independent reviewers	Tool from a previous systematic review of prevalence (De Vet, 1997; Hoogendoorn, 2000) (modified) and AHCRP type of evidence guideline	Yes	Yes	AHCPR consistency of evidence guidelines
**Fleming**	No	PRISMA	Yes	NR	Two tools from previous systematic reviews (Micha, 2010 and Herzog, 2013) and Cochrane Risk of Bias Tool	Yes	No	NA
**Foroutan**	No	PRISMA	No	NA	NA	NA	No	NA
**Frayman**	No	No	Yes	NR	No tool	No	No	NA
**French**	PROSPERO<br>CRD42015026921	PRISMA	Yes	Two independent reviewers	JBI Prevalence Critical Appraisal Tool	No	No	NA
**Fu**	No	No	Yes	NR	JBI Prevalence Critical Appraisal Tool	No	No	NA
**Fuglkjaer**	No	PRISMA	Yes	Two independent reviewers	QUIPS tool (modified)	No	No	NA
**Gane**	PROSPERO<br>CRD42014012982	PRISMA	Yes	Two independent reviewers	Critical Review Form – Quantitative Studies (Law)	No	No	NA
**Gerridzen**	No	PRISMA	Yes	Two reviewers	Boyle, 1998	No	No	NA
**Ghaemmohamadi**	No	PRISMA	Yes	Two independent reviewers	STROBE	No	No	NA
**Gheshlagh**	No	PRISMA	No	NA	NA	NA	No	NA
**Ghorbani**	No	No	Yes	NR	CASP	No	No	NA
**Gilheaney**	PROSPERO<br>CRD42016033528	PRISMA<br>MOOSE	Yes	Two independent reviewers	Dows and Black (modified) and new tool based on Boyle, 1998 and JBI Prevalence Critical Appraisal Tool	No	No	NA
**Gilheaney**	PROSPERO<br>CRD42016050846	PRISMA<br>MOOSE	Yes	Two independent reviewers	Tool from a previous systematic review of prevalence (Gilheaney, 2017)	No	No	NA
**Hackett**	PROSPERO<br>CRD42015024977	PRISMA	Yes	Two independent reviewers	JBI Prevalence Critical Appraisal Tool	No	No	NA
**Harirchian**	PROSPERO<br>CRD42016033016	No	No	NA	NA	NA	No	NA
**Harris**	No	PRISMA	No	NA	NA	NA	No	NA
**Harrisson**	PROSPERO<br>CRD42015023388	PRISMA	Yes	Two independent reviewers	Hoy, 2012	No	No	NA
**Hart**	PROSPERO<br>CRD42016035649	PRISMA	Yes	Two independent reviewers	Loney, 1998	No	No	NA
**Harvey**	PROSPERO<br>CRD42015027202	PRISMA	Yes	Three reviewers	CASP (qualitative study)	No	No	NA
**He**	No	No	Yes	NR	New tool	No	No	NA
**Heidari**	No	No	Yes	NR	STROBE	Yes	No	NA
**Heriseanu**	No	PRISMA	Yes	NR	Tool from a previous systematic review of prevalence (Ferro, 2009)	No	No	NA
**Hofstraat**	No	No	Yes	NR	Two new tools	No	No	NA

**Fang**	Yes	Random-effects	NR	Log	Yes	Yes	No	Cochran's Q<br>I^2	Egger's test<br>Funnel plot	R
**Farahani**	Yes	Random-effects	DerSimonian and Laird	Freeman–Tukey double arcsine	Yes	No	No	I^2	No	STATA
**Farrag**	No	NA	NA	NA	NA	NA	NA	NA	NA	NA
**Fishbain**	No	NA	NA	NA	NA	NA	NA	NA	NA	NA
**Fleming**	No	NA	NA	NA	NA	NA	NA	NA	NA	NA
**Foroutan**	Yes	Random-effects	DerSimonian and Laird	NR	Yes	Yes	No	Cochran's Q<br>I^2	Egger's test<br>Funnel plot	STATA
**Frayman**	No	NA	NA	NA	NA	NA	NA	NA	NA	NA
**French**	Yes	Random-effects	NR	Freeman–Tukey double arcsine	Yes	No	No	I^2	Egger's test	STATA
**Fu**	Yes	Random-effects	NR	NR	Yes	No	No	I^2<br>Sensitivity analysis	Funnel plot	Meta-Analyst
**Fuglkjaer**	No	NA	NA	NA	NA	NA	NA	NA	NA	NA
**Gane**	No	NA	NA	NA	NA	NA	NA	NA	NA	NA
**Gerridzen**	No	NA	NA	NA	NA	NA	NA	NA	NA	NA
**Ghaemmohamadi**	Yes	Random-effects	DerSimonian and Laird	NR	Yes	Yes	No	I^2<br>Sensitivity analysis	Egger's test	STATA
**Gheshlagh**	Yes	Random-effects	NR	NR	Yes	Yes	No	I^2	No	STATA
**Ghorbani**	Yes	Random-effects	DerSimonian and Laird	NR	Yes	No	No	Cochran's Q<br>I^2	No	STATA
**Gilheaney**	Yes	Random-effects	NR	NR	No	No	No	I^2	No	R
**Gilheaney**	Yes	Random-effects<br>Fixed effect	NR	NR	No	No	No	No	No	MedCalc
**Hackett**	No	NA	NA	NA	NA	NA	NA	NA	NA	NA
**Harirchian**	Yes	Random-effects	NR	NR	No	No	No	Cochran's Q<br>I^2	No	MedCalc
**Harris**	No	NA	NA	NA	NA	NA	NA	NA	NA	NA
**Harrisson**	No	NA	NA	NA	NA	NA	NA	NA	NA	NA
**Hart**	Yes	Random-effects	NR	NR	Yes	No	No	I^2<br>Sensitivity analysis	No	MedCalc
**Harvey**	No	NA	NA	NA	NA	NA	NA	NA	NA	NA
**He**	Yes	Random-effects	NR	NR	No	Yes	No	I^2	No	Open MetaAnalyst<br>STATA
**Heidari**	Yes	Random-effects	NR	NR	Yes	Yes	No	Cochran's Q<br>I^2	No	STATA
**Heriseanu**	Yes	Random-effects	NR	Freeman–Tukey double arcsine	Yes	No	No	Cochran's Q<br>I^2<br>Sensitivity analysis	No	MetaXL
**Hofstraat**	No	NA	NA	NA	NA	NA	NA	NA	NA	NA

**Hooi**	Global prevalence of Helicobacter pylori Infection: systematic review and meta-analysis	14	No	184	Yes	2	Embase<br>Medline	Titles and abstract screening: two independent reviewers (three reviewers, each one screened 2/3 of references)	No	PRISMA	Yes	NR	Newcastle-Ottawa Scale (modified)	Yes	No	NA	Yes	Random-effects	NR	NR	Yes	No	No	Cochran's Q<br>I^2	No	R
**Hu**	The prevalence and progression of mild cognitive impairment among clinic and community populations: a systematic review and meta-analysis	6	Yes	25	Yes	2	Embase<br>PubMed	Titles and abstracts screening: two independent reviewers<br>Data extraction: two independent reviewers extracted and a third reviewer checked extraction	No	No	No	NA	NA	NA	No	NA	Yes	Random-effects	NR	NR	Yes	No	No	I^2<br>Sensitivity analysis	Yes (did not report the methods)	STATA
**Ilhan**	The prevalence of neuropathic pain is high after treatment for breast cancer: a systematic review	4	Yes	15	Yes	5	CINAHL<br>Cochrane<br>Embase<br>Medline<br>Web of Science	Titles and abstract screening: two independent reviewers<br>Full text elegibility: two independent reviewers	PROSPERO<br>CRD42015029987	PRISMA	Yes	NR	Hoy, 2012	No	No	NA	Yes	Quality effects	NR	Double arcsine	Yes	No	No	Cochran's Q<br>I^2<br>Sensitivity analysis	Doi plot<br>Funnel plot<br>LFK index	MetaXL
**Islam**	Prevalence of sexual symptoms in relation to menopause in women in Asia: a systematic review	3	Yes	34	Incomplete	6	CINAHL<br>Embase<br>Google scholar<br>Medline Ovid<br>PsycINFO<br>SCOPUS	Data extraction: two independent reviewers	No	PRISMA	Yes	NR	Hoy, 2012 (modified)	No	No	NA	No	NA	NA	NA	NA	NA	NA	NA	NA	NA
**Ismail**	Prevalence, management, and prognosis of bladder cancer in patients with neurogenic bladder: a systematic review	7	NR	15	Incomplete	1	Embase	Titles and abstract screening: Two independent reviewers<br>Data extraction: two independent reviewers	PROSPERO<br>CRD42017055802	PRISMA	Yes	NR	No tool<br>Oxford Centre for Evidence-Based Medicine	No	No	NA	No	NA	NA	NA	NA	NA	NA	NA	NA	NA
**Jawad**	The prevalence and trends of waterpipe tobacco smoking: a systematic review	6	No	129	Yes	3	Embase<br>Medline<br>Web of Science	Titles and abstracts screening: two independent reviewers (four reviewers, each one screened 1/4 of references)<br>Full text elegibility: two independent reviewers (three teams)<br>Data extraction: two independent reviewers	No	PRISMA	Yes	NR	Tool from a previous systematic review of prevalence of prevalence (Herzog, 2013)	No	No	NA	No	NA	NA	NA	NA	NA	NA	NA	NA	NA
**Jimma**	Prevalence of and risk factors for multidrug-resistant tuberculosis in Iran and its neighboring countries: systematic review and meta-analysis	8	Yes	19	Yes	4	BioMed Central<br>PubMed<br>Embase<br>Web of Science	Study selection: two independent reviewers	PROSPERO<br>CRD42016038392	PRISMA	Yes	Two independent reviewers	Newcastle-Ottawa Scale	No	No	NA	Yes	Random-effects	DerSimonian and Laird	NR	No	No	No	I^2<br>Sensitivity analysis	Egger's test<br>Funnel plot<br>Trim and fill	STATA
**Jones**	A systematic review of the worldwide prevalence of survivors of poliomyelitis reported in 31 studies	5	Yes	31	Yes	5	CINAHL<br>MEDLINE<br>Psychology and Behavioral Sciences Collection<br>Scopus<br>Web of Science	Titles and abstracts screening: two independent reviewers<br>Full text elegibility: two independent reviewers	No	PRISMA	Yes	NR	Hoy, 2012	Yes	No	NA	No	NA	NA	NA	NA	NA	NA	NA	NA	NA
**Jordan (A)**	Prevalence of hepatitis C virus infection among HIV+ men who have sex with men: a systematic review and meta-analysis	6	Yes	42	Incomplete	3	BIOSIS<br>EMBASE<br>PubMed	Study screening and revisions: two reviewers	PROSPERO<br>CRD42013006462	PRISMA	Yes	NR	Newcastle-Ottawa Scale (modified)	No	No	NA	Yes	Random-effects	NR	NR	No	No	No	Cochran's Q<br>I^2	No	R<br>SPSS
**Jordan (B)**	Past-year prevalence of prescription opioid misuse among those 11 to 30 years of age in the United States: a systematic review and meta-analysis	4	Yes	19	No	4	Ovid MEDLINE<br>PsychINFO<br>PubMed<br>Web of Knowledge	Abstract screening: one reviewer (2 reviewers, each one screened half the titles after 100% agreement on pilot screenings)<br>Data extraction: one reviewer extracted (2 reviewers, each one extracted half the artiles after 100% agreement on pilot screenings), a second reviewer verfied extraction	PROSPERO<br>CDR42014008870	PRISMA	Yes	NR	Newcastle-Ottawa Scale (modified)	No	No	NA	No	NA	NA	NA	NA	NA	NA	NA	NA	NA
**Kadir**	Systematic review and meta-analysis of the birth prevalence of orofacial clefts in low- and middle-income countries	7	Yes	32	Yes	7	Africa-Wide Information<br>Embase<br>IndMED<br>Latin American and Caribbean Health Science<br>New York Academy of Medicine Grey Literature<br>Open Grey<br>PubMed/Medline	Data extraction: one reviewer extracted, a second reviewer verified extraction	No	No	Yes	NR	Unclear	Yes	No	NA	Yes	Random-effects	NR	NR	No	No	No	Cochran's Q<br>I2<br>Sensitivity analysis	No	STATA
**Kamalakannan**	Incidence and prevalence of stroke in India: a systematic review	5	Yes	10	Incomplete	5	Embase<br>IndMED<br>Medline<br>Ovid<br>PubMed	Title and abstract screening: two independent reviewers<br>Full text elegibility: two independent reviewers	No	PRISMA	Yes	Two independent reviewers	STROBE	No	No	NA	No	NA	NA	NA	NA	NA	NA	NA	NA	NA
**Karreman**	The prevalence and incidence of axial and peripheral spondyloarthritis in inflammatory bowel disease: a systematic review and meta-analysis	4	No	71	Yes	5	Embase<br>GoogleScholar<br>Medline<br>Pubmed<br>Web of Science	Title and abstract screening: one reviewer<br>Data extraction: one reviewers	No	PRISMA	Yes	Two reviewers	Hoy, 2012 (modified)	No	No	NA	Yes	Random-effects	NR	NR	Yes	No	No	I^2<br>Sensitivity analysis	No	STATA
**Kassa**	Prevalence and determinants of anemia among pregnant women in Ethiopia; a systematic review and meta-analysis	4	No	20	Yes	5	African Journals Online<br>CINAHL<br>Cochrane Library<br>Google Scholar<br>PubMed	Study assessment: two independent reviewers	No	PRISMA	Yes	Two independent reviewers	JBI Prevalence Critical Appraisal Tool	No	No	NA	Yes	Random-effects	NR	NR	Yes	No	No	I^2	No	STATA
**Kaze**	Prevalence of hypertension in older people in Africa: a systematic review and meta-analysis	5	NR	91	Yes	4	African Index Medicus<br>Embase<br>Medline<br>STEPS WHO	Titles and abstract screening: two independent reviewers<br>Full text eligibility: two independent reviewers<br>Data extraction: two independent reviewers	PROSPERO<br>CRD42016034003	PRISMA	Yes	Two independent reviewers	Hoy, 2012	No	No	NA	Yes	Random-effects	NR	Freeman–Tukey double arcsine	Yes	No	No	Cochran's Q<br>I^2<br>Sensitivity analysis	Egger's test<br>Funnel plot	STATA
**Keshavarz**	The prevalence of visual disorders in Iranian students: a meta-analysis study and systematic review	5	NR	28	Incomplete	8	Google Scholar<br>ISI<br>IranDoc<br>IranMedex<br>Magiran<br>MedLib<br>PubMed<br>SID	NR	No	No	Yes	NR	NR	Yes	No	NA	Yes	Random-effects	NR	NR	No	No	No	I^2	No	R<br>STATA
**Khalkhali**	Prevalence of cystic echinococcosis in Iran: a systematic review and meta-analysis	7	Yes	33	Incomplete	9	Google Scholar<br>Iran Doc<br>Iran Medex<br>Magiran<br>PubMed<br>Science Direct<br>Scopus<br>Scientific Information Database<br>Web of Science	Study selection: two independent reviewers	No	PRISMA	No	NA	NA	NA	No	NA	Yes	Random-effects	DerSimonian and Laird	NR	No	Yes	No	Cochran's Q<br>I^2	Egger's test<br>Funnel plot	NR
**Kincaid**	What is the prevalence of autism spectrum disorder and ASD traits in psychosis? A systematic review	4	Yes	7	Yes	3	PsychINFO<br>PubMed<br>Web of Science	Search: two independent reviewers<br>Study selection: two independent reviewers	No	PRISMA	Yes	Two reviewers	Kmet, 2004 (modified)	No	No	NA	No	NA	NA	NA	NA	NA	NA	NA	NA	NA
**King**	Prevalence rates of childhood trauma in medical students: a systematic review	4	No	11	Incomplete	4	Embase<br>Medline<br>PsychINFO<br>Web of science	Search: two independent reviewers<br>Study selection: two independent reviewers	No	No	Yes	Two independent reviewers	Kmet, 2004 (modified)	No	No	NA	No	NA	NA	NA	NA	NA	NA	NA	NA	NA
**Kingston**	A systematic review of the prevalence of comorbid mental health disorders in people presenting for substance use treatment in Australia	3	Yes	18	Unclear	5	CENTRAL<br>CINAHL<br>Embase<br>Medline<br>PsycINFO	Titles and abstract screening: one reviewer (a second reviewer screened 10% to check agreement)<br>Full text elegibility: two independent reviewers<br>Data extraction: one reviewer extracted, another reviewer verified extraction	No	No	No	NA	NA	NA	No	NA	No	NA	NA	NA	NA	NA	NA	NA	NA	NA
**Klem**	Prevalence, risk factors, and outcomes of irritable bowel syndrome after infectious enteritis: a systematic review and meta-analysis	8	Yes	45	Yes	4	Cochrane<br>Embase<br>Medline Ovid<br>Web of Science	Title and abstract screening: two independent reviewers<br>Full text elegibility: two independent reviewers<br>Data extraction: two independent reviewers	PROSPERO<br>CRD42016035317	PRISMA	Yes	Two independent reviewers	Newcastle-Ottawa Scale (modified)	No	No	NA	Yes	Random-effects	DerSimonian and Laird	NR	Yes	No	No	I^2<br>Sensitivity analysis	Egger's test<br>Funnel plot	Comprehensive Meta-Analysis
**Knapik**	Prevalence and management of coracoid fracture sustained during sporting activities and time to return to sport: a systematic review	4	NR	19	Yes	5	EMBASE, Web of Science, and Cochrane Database of Systematic Reviews	Search: two independent reviewers	No	PRISMA	No	NA	NA	NA	No	NA	No	NA	NA	NA	NA	NA	NA	NA	NA	NA
**Kojima (A)**	Prevalence of frailty in end-stage renal disease: a systematic review and meta-analysis	1	NR	7	Yes	3	CINAHL<br>Embase<br>Medline	Title and abstract screening: one reviewer<br>Full text elegibility: one reviewer<br>Data extraction: one reviewer	No	PRISMA	No	NA	NA	NA	No	NA	Yes	Random-effects	NR	NR	No	No	No	Chi-square<br>I^2	Begg's test<br>Egger's test	StatsDirect
**Kojima (B)**	Prevalence of frailty in Japan: a systematic review and meta-analysis	6	NR	5	Yes	10	AMED<br>CINAHL<br>Cochrane<br>Embase<br>ICHUSHI Web<br>LILACS<br>Medline<br>PsycINFO<br>Scopus<br>Web of Science	Title and abstract screening: one reviewer<br>Full text elegibility: one reviewer	PROSPERO<br>CRD42016036112	PRISMA	Yes	NR	Loney, 1998 (modified)	Yes	No	NA	Yes	Random-effects	NR	NR	Yes	No	No	Cochran's Q<br>I^2	Begg's test<br>Egger's test<br>Funnel plot	StatsDirect
**Kolahdooz**	Prevalence of overweight and obesity among indigenous populations in Canada: a systematic review and meta-analysis	4	Yes	57	Incomplete	6	CINAHL<br>Cochrane<br>Embase<br>ISI Web of Knowledge<br>PubMed<br>Scopus	Title and abstract screening: two independent reviewers<br>Full text elegibility: two independent reviewers<br>Data extraction: two independent reviewers	No	No	Yes	NR	New tool based on Boyle, 1998	No	No	NA	Yes	Random-effects	NR	NR	Yes	Yes	No	Cochran's Q<br>I^2	No	STATA
**Kong**	The prevalence of heterotopic ossification among patients after cervical artificial disc replacement: a systematic review and meta-analysis	6	NR	38	Yes	4	Cochrane<br>Embase<br>PubMed<br>Web of Science	Data extraction: two independent reviewers	No	PRISMA	Yes	Two independent reviewers	Loney, 1998	No	No	NA	Yes	Random-effects	NR	NR	Yes	Yes	No	Cochran's Q<br>I^2<br>Sensitivity analysis<br>Meta-regression	Begg's test<br>Egger's test<br>Funnel plot	R
**Kouidrat**	High prevalence of erectile dysfunction in diabetes: a systematic review and meta-analysis of 145 studies	9	No	145	Yes	3	Embase<br>PubMed<br>Scopus	Search: two independent reviewers<br>Data extraction: two independent reviewers extracted, a third reviewer verified extraction	No	PRISMA<br>MOOSE	Yes	Two reviewers	New tool	No	No	NA	Yes	Random-effects	NR	NR	Yes	Yes	No	Cochran's Q<br>I^2<br>Sensitivity analysis<br>Meta-regression	Egger's test<br>Funnel plot<br>Trim and fill	Comprehensive Meta-Analysis
**Kouis**	Prevalence of primary ciliary dyskinesia in consecutive referrals of suspect cases and the transmission electron microscopy detection rate: a systematic review and meta-analysis	6	Yes	36	Incomplete	3	Google Scholar<br>PubMed<br>Scopus	Title and abstract screening: two independent reviewers<br>Data extraction: two independent reviewers	No	PRISMA	Yes	NR	MOOSE (modified)	No	No	NA	Yes	Random-effects	Hartung-Knapp-Sidik-Jonkman	NR	Yes	No	No	I^2<br>Sensitivity analysis	No	STATA
**Kunzmann**	The prevalence of viral agents in esophageal adenocarcinoma and Barrett's esophagus: a systematic review	10	Yes	30	Yes	3	Embase<br>Medline<br>PubMed	Title and abstract screening: two independent reviewers<br>Full text elegibility: two reviewers<br>Data extraction: one reviewer extracted, a second reviewer verified extraction	No	No	Yes	Two reviewers	New tool based on JBI Prevalence Critical Appraisal Tool and STROME-ID for epidemiological infection studies	No	No	NA	Yes	Random-effects	Hartung-Knapp-Sidik-Jonkman	Freeman–Tukey double arcsine	No	No	No	I^2<br>Sensitivity analysis	No	STATA
**Laatikainen**	The prevalence of medication-related adverse events in inpatients-a systematic review and meta-analysis	6	No	14	Yes	4	Cochrane<br>MEDLINE<br>Scopus<br>Web of Science	Study selection: two independent reviewers<br>Data extraction: one reviewer extracted, a second reviewer verified extraction	No	PRISMA	Yes	Two reviewers	New tool	No	No	NA	Yes	Random-effects	NR	NR	No	No	No	Cochran's Q<br>I^2	No	STATA
**Lameijer**	Prevalence of posttraumatic arthritis and the association with outcome measures following distal radius fractures in non-osteoporotic patients: a systematic review	5	No	19	Yes	4	Cochrane<br>Embase<br>PsycINFO<br>PubMEd	NR	No	No	Yes	Two reviewers	Newcastle-Ottawa Scale (Cohort studies)	No	Yes	Oxford Centre for Evidence-Based Medicine	No	NA	NA	NA	NA	NA	NA	NA	NA	NA
**Lan**	Prevalence of alcohol use, sexual risk behavior, and HIV among Russians in high-risk settings: a systematic review and meta-analysis	5	Yes	18	Yes	11	CINAHL<br>Cochrane<br>CORK<br>Dissertation Abstracts<br>EMBASE<br>ERIC<br>Global Health<br>PsycINFO<br>PubMed<br>SocINDEX<br>Web of Science\	Data extraction: two independent reviewers	No	PRISMA	Yes	Two reviewers	New tool based on Jadad, 1996; Downs, 1998; and Fowkes, 1991.	No	No	NA	Yes	Random-effects	NR	Logit	No	Yes	No	Cochran's Q<br>I^2	No	STATA

**Lange (A)**	Global prevalence of fetal alcohol spectrum disorder among children and youth: a systematic review and meta-analysis	6	NR	24	Yes	7	CINHAHL<br>Embase<br>Education Resource Information Center<br>MEDLINE<br>PsychINFO<br>Scopus<br>Web of Science	Data extraction: one reviewer extracted, a second reviewer verified extraction	PROSPERO<br>CRD42016033837	PRISMA<br>MOOSE	Yes	NR	JBI Prevalence Critical Appraisal Tool (modified)	No	No	NA	Yes	Random-effects	NR	Freeman–Tukey double arcsine	No	No	No	Cochran's Q<br>I^2	Begg's test<br>Egger's test<br>Funnel plot	R
**Lange (B)**	Prevalence of binge drinking during pregnancy by country and World Health Organization region: systematic review and meta-analysis	4	No	58	Yes	14	Canadian Centre on Substance Abuse Library Collection Database<br>Campbell Collaboration<br>CINAHL<br>Cochrane<br>CSA Sociological Abstracts<br>Embase<br>ERIC<br>Google Scholar<br>Medline<br>National Institute on Alcohol Abuse and Alcoholism's Alcohol and Alcohol Problems Science Database<br>PsycINFO<br>Scopus<br>Social Work Abstracts<br>Web of Science	Title and abstract screening: two independent reviewers<br>Full text elegibility: two independent reviewers<br>Data extraction: one reviewer extracted, a second reviewer independently verified extraction	No	PRISMA	Yes	Two independent reviewers	QATSO (modified)	No	No	NA	Yes	Random-effects	NR	Freeman–Tukey double arcsine	No	No	No	Cochran's Q<br>I^2	Begg's test<br>Egger's test<br>Funnel plot	R
**Lange (C)**	Prevalence of externalizing disorders and autism spectrum disorders among children with fetal alcohol spectrum disorder: systematic review and meta-analysis	4	NR	20	Yes	7	CINAHL<br>Embase<br>ERIC<br>Medline<br>PsycINFO<br>Scopus<br>Web of Science	Title and abstract screening: two independent reviewers<br>Full text elegibility: two independent reviewers<br>Data extraction: one reviewer extracted, a second reviewer independently verified extraction	PROSPERO<br>CRD42016052041	No	Yes	Two independent reviewers	JBI Prevalence Critical Appraisal Tool	No	No	NA	Yes	Random-effects	DerSimonian and Laird	Freeman–Tukey double arcsine	No	No	No	I^2<br>Sensitivity analysis	Egger's test<br>Funnel plot	STATA
**Laszewska**	Prevalence of mental diseases in Austria: systematic review of the published evidence	4	Yes	19	Yes	7	CINAHL<br>Embase<br>MEDLINE<br>PsycINFO<br>PSYNDEXplus<br>Scopus<br>Social Science Citation Index (SSCI)	NR	No	PRISMA	Yes	NR	Loney, 1998	No	No	NA	No	NA	NA	NA	NA	NA	NA	NA	NA	NA
**Lee**	HPV prevalence in the foreskins of asymptomatic healthy infants and children: systematic review and meta-analysis	4	Yes	8	Incomplete	3	Cochrane<br>Embase<br>Medline	Title and abstract screening: two independent reviewers<br>Full text elegibility: two independent reviewers<br>Data extraction: two independent reviewers	No	PRISMA	Yes	Two independent reviewers	QUIPS tool	No	No	NA	Yes	Random-effects	DerSimonian and Laird	Freeman–Tukey double arcsine	No	Yes	No	Cochran's Q<br>I^2<br>Sensitivity analysis<br>Galbraith plot	Begg's test<br>Egger's test<br>Funnel plot	R
**Leverment**	Prevalence and factors associated with disturbed sleep in patients with ankylosing spondylitis and non-radiographic axial spondyloarthritis: a systematic review	4	NR	15	Incomplete	8	AMED<br>CINAHL<br>Embase<br>Medline<br>PsycINFO<br>PubMed<br>Scopus<br>Web of Science	Study selection: two independent reviewers<br>Data extraction: two independent reviewers	No	PRISMA	Yes	Two independent reviewers	New tool	Yes	No	NA	No	NA	NA	NA	NA	NA	NA	NA	NA	NA
**Li**	The prevalence of acne in Mainland China: a systematic review and meta-analysis	6	NR	25	Yes	6	China National Knowledge Infrastructure<br>EMBASE<br>PubMed<br>VIP Database for Chinese Technical Periodicals<br>Wan Fang Database for Chinese Periodicals<br>Web of Science	Title and abstract screening: two independent reviewers<br>Data extraction: two independent reviewers	No	No	Yes	Two independent reviewers	Tool from a previous systematic review of prevalence (Say, 2006)	No	No	NA	Yes	Random-effects	NR	NR	Yes	Yes	No	Cochran's Q<br>I^2	Egger's test<br>Funnel plot	Comprehensive Meta-Analysis
**Lim**	Prevalence and determinants of overweight and obesity in children and adolescents from migrant and seasonal farmworker families in the United States - a systematic review and qualitative assessment	3	NR	10	Incomplete	Unclear	*Michigan State University Libraries' Search Plus search engine*	Title and abstract screening: two independent reviewers<br>Full text elegibility: two independent reviewers<br>Data extraction: one reviewer	No	PRISMA	Yes	Two independent reviewers	Academy of Nutrition and Dietetics's Quality Criteria Checklist for Primary Research from the Evidence Analysis Manual: Steps in the Academy Evidence Analysis Proces (10 questions + overall)	No	No	NA	No	NA	NA	NA	NA	NA	NA	NA	NA	NA
**Lin**	The prevalence, metabolic risk and effects of lifestyle intervention for metabolically healthy obesity: a systematic review and meta-analysis: a PRISMA-compliant article	4	Yes	40	Incomplete	4	Cochrane<br>Embase<br>Medline<br>Web of Science	Title and abstract screening: two reviewers<br>Full text elegibility: two reviewers<br>Data extraction: one reviewer extracted, a second reviewer verified extraction	No	PRISMA	No	NA	NA	NA	No	NA	Yes	Random-effects	NR	Arcsine square roots	Yes	Yes	No	I^2<br>Sensitivity analysis	Egger's test	STATA
**Lorentzen**	Prevalence of polypoidal choroidal vasculopathy in white patients with exudative age-related macular degeneration: systematic review and meta-analysis	3	NR	10	Yes	4	Cochrane<br>Embase<br>PubMed<br>Web of Science	Full text elegibility: two independent reviewers<br>Data extraction: two reviewers	No	PRISMA<br>MOOSE	Yes	NR	Agency for Healthcare Research and Quality checklist for cross-sectional studies (modified)	No	No	NA	Yes	Random-effects	NR	Double arcsine	Yes	No	No	Cochran's Q<br>I^2<br>Sensitivity analysis	Funnel plot	MetaXL
**Lundorff**	Prevalence of prolonged grief disorder in adult bereavement: a systematic review and meta-analysis	5	NR	14	Yes	5	CINAHL<br>Embase<br>PsycINFO<br>PubMed<br>Web of Science	Search: two independent reviewers<br>Title and abstract screening: two independent reviewers<br>Full text elegibility: two independent reviewers	PROSPERO<br>CRD42016038416	PRISMA<br>MOOSE	Yes	NR	Hoy, 2012	No	No	NA	Yes	Random-effects	NR	NR	Yes	Yes	Yes	Cochran's Q<br>I^2<br>Sensitivity analysis	Egger's test<br>Funnel plot<br>Trim and fill	Comprehensive Meta-Analysis
**Mairghani**	The prevalence and incidence of diabetic foot ulcers among five countries in the Arab world: a systematic review	7	NR	9	Yes	7	CINAHL<br>EBSCO<br>Embase<br>Global Health<br>PubMed<br>Web of Science<br>Scopus	Data extraction: two independent reviewers	No	PRISMA	Yes	Two reviewers	Glynn, 2006	No	No	NA	No	NA	NA	NA	NA	NA	NA	NA	NA	NA
**Maroufizadeh (A)**	Prevalence of diabetic retinopathy in Iran: a systematic review and meta-analysis	5	NR	31	Incomplete	7	Google Scholar<br>Iranmedex<br>Magiran<br>PubMed<br>Scopus<br>SID<br>Web of Science	Data extraction: two independent reviewers	No	PRISMA	Yes	Two independent reviewers	STROBE	No	No	NA	Yes	Random-effects	NR	NR	Yes	Yes	No	Cochran's Q<br>I^2<br>Sensitivity analysis<br>Galbraith plot	Begg's test<br>Egger's test<br>Funnel plot	STATA
**Maroufizadeh (B)**	Prevalence of retinopathy of prematurity in Iran: a systematic review and meta-analysis	4	NR	26	Incomplete	7	Google Scholar<br>Iranmedex<br>Magiran<br>PubMed<br>Scopus<br>SID<br>Web of Science	Study selection: two independent reviewers	No	No	Yes	NR	STROBE (modified)	No	No	NA	Yes	Random-effects	NR	NR	Yes	Yes	No	Cochran's Q<br>I^2<br>Sensitivity analysis<br>Galbraith plot	Begg's test<br>Egger's test<br>Funnel plot<br>Trim and fill	STATA
**McWilliams**	A systematic review of the prevalence of comorbid cancer and dementia and its implications for cancer-related care	6	Yes	47	Yes	5	BNI<br>CINAHL<br>Embase<br>MEDLINE<br>PsycINFO	Study selection: two independent reviewers	No	PRISMA	Yes	NR	Mixed Methods Appraisal Tool (MMAT)	No	No	NA	No	NA	NA	NA	NA	NA	NA	NA	NA	NA
**Medisauskaite**	Prevalence of oncologists in distress: systematic review and meta-analysis	2	NR	43	Yes	1	Web of Science	NR	PROSPERO<br>CRD42015016325	PRISMA	Yes	NR	New tool	No	No	NA	Yes	Random-effects	NR	Double arcsine	No	No	No	Cochran's Q<br>I^2<br>Sensitivity analysis	Funnel plot<br>LFK index	MetaXL
**Melese**	The prevalence of tuberculosis among prisoners in Ethiopia: a systematic review and meta-analysis of published studies	2	No	10	Yes	3	Cochrane<br>Google Scholar<br>Medline	Title and abstract screening: two independent reviewers<br>Full text elegibility: two independent reviewers	No	PRISMA	Yes	Two independent reviewers	New tool	Yes	No	NA	Yes	Random-effects	DerSimonian and Laird	Double arcsine	Yes	No	No	Cochran's Q<br>I^2<br>Sensitivity analysis	Doi plot<br>LFK index	MetaXL
**Minervini**	Positive surgical margins and local recurrence after simple enucleation and standard partial nephrectomy for malignant renal tumors: systematic review of the literature and meta-analysis of prevalence	11	NR	33	Yes	3	Embase<br>MedLine<br>Web of Science	Search: two independent reviewers<br>Data extraction: two independent reviewers	No	PRISMA	No	NA	NA	NA	No	NA	Yes	Random-effects<br>Fixed effect	DerSimonian and Laird	NR	No	No	No	I^2<br>Sensitivity analysis	No	R
**Moghoofei**	Prevalence of Influenza A infection in the Middle-East: a systematic review and meta-analysis	11	No	71	Incomplete	5	Barakat Knowledge Network System<br>PubMed<br>Scopus<br>SID<br>Web of Science	Data extraction: three independent reviewers	No	PRISMA	Yes	NR	STROBE	Yes	No	NA	Yes	Random-effects	NR	Freeman–Tukey double arcsine	Yes	Yes	No	Cochran's Q<br>I^2<br>Sensitivity analysis	Begg's test<br>Egger's test	STATA
**Mohammadi**	Prevalence and major causes of visual impairment in Iranian adults: a systematic review	7	Yes	8	Yes	7	Barakat<br>Iran Eye Doc<br>MagIran<br>PubMed<br>SID<br>Scopus<br>Web of Science	Title and abstract screening: two independent reviewers<br>Data extraction: two independent reviewers	No	No	Yes	Two independent reviewers	No tool	Yes	No	NA	Yes	Random-effects	NR	NR	No	Yes	No	Cochran's Q<br>I^2<br>Meta-regression	Funnel plot	STATA
**Mohseni**	A systematic review on the prevalence of acute myocardial infarction in Iran	4	Yes	18	Incomplete	16	PubMed<br>Scopus<br>ProQuest<br>Institute for Scientific Information<br>Web of Science<br>ScienceDirect<br>Wiley online library<br>Springer<br>Ovid<br>Cochrane Library<br>Medlib<br>Barekat<br>Magiran<br>Scientific Information Database<br>Ganj<br>Google scholar	NR	No	No	Yes	NR	STROBE	No	No	NA	No	NA	NA	NA	NA	NA	NA	NA	NA	NA
**Mokhayeri**	Metabolic syndrome prevalence in the Iranian adult's general population and its trend: a systematic review and meta-analysis of observational studies	5	No	26	Yes	11	Cochrane<br>Embase<br>Google Scholar<br>Irandoc<br>IranMedex<br>Magiran<br>PubMed<br>Science Direct<br>Scopus<br>SID<br>Web of Science	Title and abstract screening: two independent reviewers<br>Data extraction: two independent reviewers	No	PRISMA<br>MOOSE	Yes	Two reviewers	JBI Prevalence Critical Appraisal Tool	Yes	No	NA	Yes	Random-effects	NR	Freeman–Tukey double arcsine	Yes	Yes	No	Cochran's Q<br>I^2<br>Galbraith plot	No	STATA
**Molendijk**	Prevalence rates of the incubus phenomenon: a systematic review and meta-analysis	6	No	13	Yes	3	Embase<br>PsycINFO<br>PubMed	Search: at least two independent reviewers<br>Study selection: at least two independent reviewers<br>Data extraction: at least two independent reviewers	No	PRISMA	Yes	Two independent reviewers	Newcastle-Ottawa Scale (modified)	No	No	NA	Yes	Random-effects	NR	Double arcsine	Yes	No	No	I^2<br>Sensitivity analysis	No	STATA

**Moosazadeh**	Prevalence of familial multiple sclerosis in Iran: a systematic review and meta-analysis	7	NR	15	Incomplete	8	Google Scholar<br>Irandoc<br>Iranmedex<br>Magiran<br>PubMed<br>Scopus<br>SID<br>Web of Science	Search: two independent reviewers	No	No	Yes	NR	STROBE	Yes	No	NA	Yes	Random-effects	NR	NR	No	Yes	No	Cochran's Q<br>I^2<br>Sensitivity analysis	No	STATA
**Moosazadeh (B)**	Prevalence of Enterobius vermicularis among children in Iran: a systematic review and meta-analysis	6	NR	19	Incomplete	7	Google Scholar<br>Irandoc<br>Magiran<br>PubMed<br>Scopus<br>Science Direct<br>SID (Scientific Information Database)	Search: two independent reviewers<br>Data extraction: two independent reviewers	No	No	Yes	NR	Tool from a previous systematic review of prevalence (Moosazadeh, 2013)	Yes	No	NA	Yes	Random-effects	NR	NR	Yes	Yes	No	Cochran's Q<br>I^2<br>Sensitivity analysis	No	STATA
**Morais**	Contemporary migration patterns in the prevalence of Helicobacter pylori infection: a systematic review	5	Yes	28	Yes	1	PubMed	Titles and abstracts screening: two independent reviewers (three reviewers)<br>Data extraction: two independent reviewers	No	No	No	NA	NA	NA	No	NA	No	NA	NA	NA	NA	NA	NA	NA	NA	NA
**Moynan**	Prevalence of head injury and associated disability in prison populations: a systematic review	2	Yes	10	Incomplete	5	Cochrane<br>Embase Ovid<br>MEDLINE Ovid<br>PsycINFO<br>PubMed	Study selection: one reviewer	No	No	Yes	Two independent reviewers	New tool based on criteria developed for epidemiological studies (according to Sanderson, 2007)	No	No	NA	No	NA	NA	NA	NA	NA	NA	NA	NA	NA
**Mozhgani**	Prevalence of human influenza virus in Iran: evidence from a systematic review and meta-analysis	6	NR	32	Incomplete	4	PubMed<br>ScienceDirect<br>Scopus<br>Web of Science	Title and abstract screening: two independent reviewers<br>Full text elegibility: two independent reviewers	No	No	Yes	NR	Hoy, 2012 (modified)	No	No	NA	Yes	Random-effects	NR	NR	Yes	No	No	Cochran's Q<br>I^2	Egger's test<br>Funnel plot	Review Manager
**Munabi**	The prevalence of congenital heart disease in nonsyndromic cleft lip and/or palate: a systematic review of the literature	6	No	12	Incomplete	1	PubMed	NR	No	PRISMA	No	NA	NA	NA	No	NA	No	NA	NA	NA	NA	NA	NA	NA	NA	NA
**Musa**	Trends in prevalence of multi drug resistant tuberculosis in sub-Saharan Africa: a systematic review and meta-analysis	6	No	51	Yes	8	African Journals Online (AJOL)<br>BIOLINE Cochrane<br>Embase<br>Google Scholar<br>HINARI<br>Institute of Science Information (ISI)<br>PubMed/Medline<br>Scopus	Search: three independent reviewers	No	PRISMA<br>MOOSE	Yes	Two independent reviewers	NIH Quality Assessment Tool for Observational Cohort and Cross-Sectional Studies	Yes	No	NA	Yes	Random-effects	DerSimonian and Laird	NR	Yes	Yes	No	Cochran's Q<br>I^2<br>Sensitivity analysis	Begg's test<br>Egger's test<br>Funnel plot<br>Trim and fill	STATA
**Nascimento**	Prevalence of transactive response DNA-binding protein 43 (TDP-43) proteinopathy in cognitively normal older adults: systematic review and meta-analysis	13	Yes	7	Yes	3	Embase<br>PubMed<br>Web of Science	Search: three independent reviewers<br>Titles and abstract screening: two independent reviewers<br>Full text elegibility: two reviewers	No	PRISMA<br>MOOSE	Yes	Two independent reviewers	JBI Prevalence Critical Appraisal Tool (modified)	No	No	NA	Yes	Random-effects	NR	Log	Yes	No	No	Cochran's Q<br>I^2	Begg's test<br>Egger's test<br>Funnel plot	STATA
**Ng**	Worldwide incidence and prevalence of inflammatory bowel disease in the 21st century: a systematic review of population-based studies	12	No	147	Yes	2	Embase<br>Medline	Study selection: two independent reviewers<br>Data extraction: two independent reviewers	No	MOOSE	Yes	NR	Tool from a previous systematic review of prevalence (Kaplan, 2016)	No	No	NA	No	NA	NA	NA	NA	NA	NA	NA	NA	NA
**Nguyen**	A systematic review and meta-analysis of the prevalence of norovirus in cases of gastroenteritis in developing countries	5	NR	178	Yes	2	PubMed<br>Web of Science	NR	No	No	No	NA	NA	NA	No	NA	Yes	NR	NR	NR	Yes	No	No	I^2	No	STATA
**Nilsson**	Definitions, measurements and prevalence of fear of childbirth: a systematic review	10	No	24	Yes	5	CINAHL<br>Cochrane<br>PsycINFO<br>PubMed<br>Scopus	Study selection: two reviewers<br>Data extraction: one reviewer extracted, a second reviewer verified extraction	No	PRISMA	Yes	Two reviewers	Unclear	No	No	NA	No	NA	NA	NA	NA	NA	NA	NA	NA	NA
**Noubiap**	Prevalence of elevated blood pressure in children and adolescents in Africa: a systematic review and meta-analysis	6	No	47	Yes	4	African Index Medicus<br>African Journals Online<br>Embase<br>PubMed	Title and abstract screening: two independent reviewers<br>Data extraction: two independent reviewers	PROSPERO<br>CRD42015019029<br>Published protocol	No	Yes	Two independent reviewers	Hoy, 2012	No	No	NA	Yes	Random-effects	NR	Freeman–Tukey double arcsine	Yes	Yes	No	Cochran's Q<br>I^2	Egger's test<br>Funnel plot	STATA
**O'Connell**	Worldwide prevalence of tocophobia in pregnant women: systematic review and meta-analysis	5	Yes	33	Yes	6	CINAHL<br>Maternity & Infant Care<br>MEDLINE<br>PsycINFO<br>PubMed<br>Scopus	Title and abstract screening: two independent reviewers<br>Full text elegibility: two independent reviewers<br>Data extraction: two reviewers	PROSPERO<br>CRD42015017443	PRISMA	Yes	Two independent reviewers	Tool from a previous systematic review of prevalence (Knight, 2012)	No	No	NA	Yes	Random-effects	NR	NR	Yes	No	No	I^2	No	STATA
**Onyedum**	Prevalence of drug-resistant tuberculosis in Nigeria: a systematic review and meta-analysis	3	No	34	Yes	8	AJOL<br>Cochrane<br>Embase<br>Google Scholar<br>HINARI<br>PubMed<br>Scopus<br>Web of Science	Title and abstract screening: two independent reviewers<br>Data extraction: two independent reviewers	No	PRISMA	Yes	Two independent reviewers	JBI Prevalence Critical Appraisal Tool	No	No	NA	Yes	Random-effects	NR	NR	Yes	No	No	Cochran's Q<br>I^2	Begg's test<br>Funnel plot	Comprehensive Meta-Analysis<br>STATA
**Oosterwijk**	Prevalence of scar contractures after burn: a systematic review	6	Yes	10	No	4	CINAHL<br>Cochrane<br>PEDro<br>Pubmed	Title and abstract screening: two independent reviewers<br>Full text elegibility: at least two reviewers	No	PRISMA	Yes	NR	JBI Prevalence Critical Appraisal Tool (modified)	No	No	NA	No	NA	NA	NA	NA	NA	NA	NA	NA	NA
**Oqab**	Prevalence of pulmonary embolism in patients presenting with syncope. A systematic review and meta-analysis	3	No	12	Yes	5	CINAHL<br>Embase<br>LILACS<br>Medline<br>Web of Science	Title and abstract screening: two independent reviewers<br>Data extraction: two independent reviewers	PROSPERO<br>CRD42017056798	PRISMA	Yes	Two independent reviewers	Tool from a previous systematic review of prevalence (Pournazari, 2017)	No	No	NA	Yes	Random-effects	NR	NR	No	No	No	I^2	No	Comprehensive Meta-Analysis
**Osinubi**	The prevalence of psychological comorbidity in people with vitiligo: a systematic review and meta-analysis	8	No	29	Yes	4	CINAHL<br>Embase (Ovid)<br>Medline (OVID)<br>PsychINFO	Title and abstract screening: two independent reviewers<br>Full text elegibility: two independent reviewers<br>Data extraction: two independent reviewers	PROSPERO<br>CRD42016036193	PRISMA	Yes	Three independent reviewers	New tool based on Newcastle-Ottawa and the National Heart Lung and Blood Institute scales	No	No	NA	Yes	Random-effects	DerSimonian and Laird	NR	No	No	No	I^2	No	STATA
**Ouattara**	Prevalence, genetic variants and clinical implications of G-6-PD deficiency in Burkina Faso: a systematic review	10	No	8	Yes	3	Google Scholar<br>PubMed<br>Science Direct	Title and abstract screening: two independent reviewers<br>Full text elegibility: two independent reviewers	No	No	No	NA	NA	NA	No	NA	No	NA	NA	NA	NA	NA	NA	NA	NA	NA
**Oudshoorn**	Prevalence of laceration injuries in professional and amateur rugby union: a systematic review and meta-analysis	5	Yes	12	Yes	4	Ovid<br>PubMed<br>Scopus<br>Web of Science	Title and abstract screening: one reviewer	PROSPERO<br>CRD42015024027	PRISMA	Yes	NR	Tool from a previous systematic review of prevalence (Walden, 2015)	Yes	No	NA	Yes	NR	NR	NR	No	No	No	NR	No	NR
**Ozcan**	Postpartum depression prevalence and risk factors in Turkey: a systematic review and meta-analysis	3	No	52	Incomplete	7	CINAHL<br>Cochrane<br>Medline<br>OVID<br>PsycINFO<br>Pubmed<br>Science Direct	Study selection: two independen reviewers	No	PRISMA	Yes	Two independent reviewers	New tool based on Polit and Beck, 2009	Yes	No	NA	Yes	Random-effects	NR	NR	No	No	No	Cochran's Q<br>I^2	No	Comprehensive Meta-Analysis
**Pamoukdjian**	Prevalence and predictive value of pre-therapeutic sarcopenia in cancer patients: a systematic review	6	NR	35	Yes	1	PubMed	Title and abstract screening: two independent reviewers<br>Full text elegibility: two independent reviewers	No	PRISMA	Yes	NR	ACROBAT-NRSI<br>NIH Quality Assessment Tool for Observational Cohort and Cross-Sectional Studies	No	No	NA	No	NA	NA	NA	NA	NA	NA	NA	NA	NA
**Parola**	Prevalence of burnout in health professionals working in palliative care: a systematic review	5	Yes	8	Yes	4	CINAHL<br>PubMed<br>Scielo<br>Scopus	Data extraction: two independent reviewers	Published protocol	PRISMA	Yes	Two independent reviewers	JBI Prevalence Critical Appraisal Tool	Yes	Yes	JBI Grades of Recommendation	No	NA	NA	NA	NA	NA	NA	NA	NA	NA
**Pasha**	Prevalence of birth defects in Iran: a systematic review and meta-analysis	5	NR	42	Yes	5	Google Scholar<br>Magiran<br>PubMed<br>Scopus<br>Scientific Information Database (SID)	Title and abstract screening: two independent reviewers	No	PRISMA	No	NA	NA	NA	No	NA	Yes	Random-effects	NR	NR	No	No	No	I^2	No	Comprehensive Meta-Analysis
**Paz**	Systematic review of published data on the worldwide prevalence of John Cunningham virus (JCV) in patients with multiple sclerosis	5	No	18	Yes	6	Embase<br>Google Scholar<br>LILACS<br>Medline<br>PubMed<br>SciELO	NR	No	PRISMA	No	NA	NA	NA	No	NA	No	NA	NA	NA	NA	NA	NA	NA	NA	NA
**Pereira**	Prevalence of adenoid hypertrophy: a systematic review and meta-analysis	7	No	22	No	7	EMB reviews<br>Embase<br>Google scholar<br>LILACS<br>MEDLINE<br>PubMed<br>Web of Science	Title and abstract screening: two independent reviewers<br>Full text elegibility: two reviewers<br>Data extraction: one reviewer extracted, a second reviewer verified extraction	PROSPERO<br>CRD42016039375	PRISMA	Yes	Two independent reviewers	JBI Prevalence Critical Appraisal Tool (Matsari)	No	Yes	GRADE	Yes	Random-effects<br>Fixed effect	NR	NR	No	No	No	I^2	No	MedScale
**Perrin**	The prevalence of diabetes-specific emotional distress in people with type 2 diabetes: a systematic review and meta-analysis	5	Yes	55	Yes	7	ASSIA<br>CINAHL<br>Cochrane Library<br>Embase<br>MEDLINE<br>PsychINFO<br>Scopus	Title and abstract screening: two independent reviewers<br>Data extraction: one reviewer	No	No	Yes	NR	Loney, 1998	No	No	NA	Yes	Random-effects<br>Fixed effect	NR	Freeman–Tukey double arcsine	Yes	Yes	No	I^2	Egger's test<br>Funnel plot	STATA
**Pestana**	Prevalence of voice disorders in singers: systematic review and meta-analysis	3	No	11	Yes	4	CINAHL<br>PubMed<br>Medline<br>Web of Science	NR	No	No	No	NA	NA	NA	No	NA	Yes	Random-effects<br>Fixed effect	NR	NR	No	No	No	I^2	No	MedCalc
**Popova**	Estimation of national, regional, and global prevalence of alcohol use during pregnancy and fetal alcohol syndrome: a systematic review and meta-analysis	5	Yes	392	Yes	14	Canadian Centre on Substance Abuse Library Collection Database<br>Campbell Collaboration<br>CINAHL<br>Cochrane<br>CSA Sociological Abstracts<br>Embase<br>ERIC<br>Google Scholar<br>MEDLINE<br>National Institute on Alcohol Abuse and Alcoholism's Alcohol and Alcohol Problems Science Database<br>PsycINFO<br>Scopus<br>Social Work Abstracts<br>Web of Science	Title and abstract screening: two independent reviewers<br>Full text elegibility: two independent reviewers<br>Data extraction: one reviewer extracted, a second reviewer independently verified extraction	PROSPERO<br>CRD42016033835 and CRD42016033837	No	Yes	Two independent reviewers	JBI Prevalence Critical Appraisal Tool<br>QATSO (modified)	No	No	NA	Yes	Random-effects	DerSimonian and Laird	Double arcsine	No	No	No	Cochran's Q<br>I^2	Begg's test<br>Egger's test<br>Funnel plot	R
**Porter**	Systematic review links the prevalence of intraductal carcinoma of the prostate to prostate cancer risk categories	10	Yes	24	Incomplete	3	Medline Ovid<br>PubMed<br>Scopus	Full text elegibility: two independent reviewers	No	PRISMA	No	NA	NA	NA	No	NA	No	NA	NA	NA	NA	NA	NA	NA	NA	NA
**Porto**	Prevalence of otologic signs and symptoms in adult patients with temporomandibular disorders: a systematic review and meta-analysis	7	No	8	Yes	8	Google Scholar<br>LILACS<br>LIVIVO<br>OpenGrey<br>ProQuest<br>PubMed<br>Scopus<br>Web of Science	Title and abstract screening: two independent reviewers<br>Full text elegibility: two reviewers<br>Data extraction: one reviewer extracted, a second reviewer verified extraction	PROSPERO<br>CRD42015020826	PRISMA	Yes	Two independent reviewers	JBI Prevalence Critical Appraisal Tool	No	No	NA	Yes	Random-effects	NR	NR	No	No	No	I^2	No	MedCalc

**Qin (A)**	Worldwide prevalence of adverse pregnancy outcomes among singleton pregnancies after in vitro fertilization/intracytoplasmic sperm injection: a systematic review and meta-analysis	7	Yes	52	Incomplete	7	China Biology Medicine disc (CBMdisc)<br>China National Knowledge Infrastructure (CNKI)Chinese Scientific Journals Full-text Database (CQVIP)<br>Cochrane<br>Google Scholar<br>PubMed<br>Wanfang Data	NR	No	PRISMA<br>MOOSE	Yes	NR	Newcastle-Ottawa Scale (modified)	No	No	NA	Yes	Random-effects	NR	NR	Yes	No	No	Cochran's Q<br>I^2<br>Sensitivity analysis	Begg's test	R
**Qin (B)**	Worldwide prevalence of adverse pregnancy outcomes associated with in vitro fertilization/intracytoplasmic sperm injection among multiple births: a systematic review and meta-analysis based on cohort studies	7	Yes	64	No	7	Cochrane Libraries<br>Google Scholar<br>China Biology Medicine disc (CBMdisc)<br>China National Knowledge Infrastructure (CNKI)<br>Chinese Scientific Journals Full text Database (CQVIP)<br>PubMed<br>Wanfang Data	Abstract screening: two independent reviewers<br>Full text elegibility: two independent reviewers<br>Data extraction: two independent reviewers	No	PRISMA<br>MOOSE	No	NA	NA	NA	No	NA	Yes	Random-effects	NR	NR	Yes	No	No	Cochran's Q<br>I^2<br>Sensitivity analysis	Begg's test	R
**Rabbitts**	Prevalence and predictors of chronic postsurgical pain in children: a systematic review and meta-analysis	4	Yes	13	Yes	4	Cochrane<br>Embase<br>MedLine<br>PsycINFO	Title and abstract screening: one reviewer screened, another reviewer screened a sample<br>Full text elegibility: two reviewers<br>Data extraction: one reviewer extracted, a second reviewer verified extraction	No	PRISMA	Yes	Two reviewers	QUIPS tool	No	No	NA	No	NA	NA	NA	NA	NA	NA	NA	NA	NA
**Rakesh**	Prevalence of anaemia in Kerala State, Southern India - a systematic review	1	No	10	Incomplete	2	Google Scholar<br>PubMed	NR	No	PRISMA	Yes	NR	Centre for reviews and dissemination, University of York	Yes	No	NA	No	NA	NA	NA	NA	NA	NA	NA	NA	NA
**Ramirez**	Prevalence and risk factors for osteoporosis and fractures in axial spondyloarthritis: a systematic review and meta-analysis	5	Yes	46	Yes	3	Cochrane<br>Embase<br>Medline	Title and abstract screening: two independent reviewers	No	No	Yes	NR	JBI Prevalence Critical Appraisal Tool	No	No	NA	Yes	Random-effects	Restricted maximum-likelihood	Double arcsine	No	No	No	I^2	NA	R
**Ranjbaran**	Prevalence of premenstrual syndrome in Iran: a systematic review and meta-analysis	5	Yes	24	Yes	7	Google Scholar<br>Iranmedex<br>Magiran<br>PubMed<br>Scientific Information database (SID)<br>Scopus<br>Web of Knowledge	NR	No	PRISMA	Yes	Two independent reviewers	STROBE	No	No	NA	Yes	Random-effects	NR	NR	Yes	Yes	No	Cochran's Q<br>I^2	No	STATA
**Ravaghi**	Prevalence of depression in hemodialysis patients in Iran: a systematic review and meta-analysis	7	NR	27	Incomplete	9	Embase<br>IranDoc<br>IranMedex<br>Magiran<br>Ovid<br>PubMed<br>Scopus<br>SID<br>Web of Science	Title and abstract screening: two independent reviewers	No	PRISMA	Yes	Two reviewers	STROBE (modified)	No	No	NA	Yes	Random-effects	DerSimonian and Laird	NR	Yes	Yes	No	Cochran's Q<br>I^2<br>Sensitivity analysis	Egger's test	STATA
**Rees**	The worldwide incidence and prevalence of systemic lupus erythematosus: a systematic review of epidemiological studies	5	Yes	76	Yes	2	Embase<br>Medline Ovid	Data extraction: one reviewer	No	PRISMA	No	NA	NA	NA	No	NA	No	NA	NA	NA	NA	NA	NA	NA	NA	NA
**Rezaei**	Period prevalence and reporting rate of needlestick injuries to nurses in Iran: a systematic review and meta-analysis	5	NR	21	Incomplete	6	Iranmedex<br>Institute for Science Information<br>Magiran<br>PubMed<br>Scientific Information Database (SID)<br>Scopus	Study selection: two independen reviewers	No	No	Yes	NR	Tool from a previous systematic review of prevalence of prevalence (Moosazadeh, 2013)	Yes	No	NA	Yes	Random-effects	NR	NR	No	Yes	No	Cochran's Q<br>I^2	Egger's test<br>Funnel plot	STATA
**Rezaei**	Prevalence of burnout among nurses in Iran: a systematic review and meta-analysis	5	Yes	21	Incomplete	6	Institute for Scientific Information Database (ISI)<br>IranMedex<br>Magiran<br>PubMed<br>SID<br>Scopus	Study selection: two independent reviewers	No	PRISMA	Yes	NR	Tool from a previous systematic review of prevalence of prevalence (Moosazadeh, 2013)	Yes	No	NA	Yes	Random-effects	NR	NR	No	Yes	No	Cochran's Q<br>I^2	Egger's test<br>Funnel plot	STATA
**Ribeiro**	Prevalence of body dysmorphic disorder in plastic surgery and dermatology patients: a systematic review with meta-analysis	1	NR	33	Incomplete	3	CENTRAL<br>PubMed<br>Scielo	NR	No	No	No	NA	NA	NA	Yes	NR	No	NA	NA	NA	NA	NA	NA	NA	NA	NA
**Rich**	Racial and ethnic disparities in nonalcoholic fatty liver disease prevalence, severity, and outcomes in the United States: a systematic review and meta-analysis	8	Yes	34	Yes	3	Cochrane<br>EMBASE Ovid<br>Medline Ovid	Title and abstract screening: two independent reviewers<br>Full text elegibility: two independent reviewers<br>Data extraction: two independent reviewers	No	PRISMA	Yes	NR	NIH Quality Assessment Tool for Observational Cohort and Cross-Sectional Studies	No	No	NA	No	NA	NA	NA	NA	NA	NA	NA	NA	NA
**Robertson**	Prevalence of dysphagia in people with intellectual disability: a systematic review	5	Yes	20	Yes	4	CINAHL<br>Medline<br>PsycINFO<br>Web of Science	Full text elegibility: two reviewers<br>Data extraction: one reviewer	No	No	Yes	NR	New tool based on JBI Prevalence Critical Appraisal Tool	No	No	NA	No	NA	NA	NA	NA	NA	NA	NA	NA	NA
**Rouhi**	Prevalence and risk factors for liver fibrosis detected by transient elastography or shear wave elastography in inflammatory arthritis: a systematic review	5	Yes	10	Unclear	3	Embase<br>MedLine<br>Web of Science	Title and abstract screening: two independent reviewers<br>Full text elegibility: two independent reviewers<br>Data extraction: two independent reviewers	PROSPERO<br>CRD42016041914	PRISMA	Yes	Two independent reviewers	Tool from a previous systematic review of prevalence (Marlais, 2016) (modified)	No	No	NA	No	NA	NA	NA	NA	NA	NA	NA	NA	NA
**Salmanian**	A systematic review on the prevalence of conduct disorder in the Middle East	3	Yes	24	Incomplete	5	Google Scholar<br>Index Medicus for the Eastern Mediterranean Region (IMEMR)<br>Islamic World Science Citation Center (ISC)<br>PubMed<br>Scopus	Full text elegibility: two independent reviewers<br>Data extraction: two independent reviewers	Published protocol	No	Yes	Two independent reviewers	STROBE	No	No	NA	No	NA	NA	NA	NA	NA	NA	NA	NA	NA
**Schnippel**	Adverse drug reactions during drug-resistant TB treatment in high HIV prevalence settings: a systematic review and meta-analysis	5	Yes	24	Yes	2	PubMed<br>Scopus	NR	No	No	No	NA	NA	NA	No	NA	Yes	NR	NR	NR	Yes	No	No	I^2	No	STATA
**Scholz-Kreisel**	Prevalence of cardiovascular late sequelae in long-term survivors of childhood cancer: a systematic review and meta-analysis	8	NR	63	Yes	2	PubMed<br>Web of Science	Title and abstract screening: one reviewer<br>Full text elegibility: two independent reviewers	No	PRISMA	No	NA	NA	NA	No	NA	Yes	Random-effects	NR	NR	No	No	No	Cochran's Q<br>I^2	No	NR
**Scotti**	Prevalence and incidence of psoriatic arthritis: a systematic review and meta-analysis	4	Yes	28	Yes	1	Medline	Study selection: two independent reviewers	No	No	No	NA	NA	NA	No	NA	Yes	Random-effects	DerSimonian and Laird	Logit	Yes	No	No	Cochran's Q<br>I^2<br>Sensitivity analysis	Egger's test	R
**Senaratna**	Prevalence of obstructive sleep apnea in the general population: a systematic review	8	No	24	Yes	2	Embase Ovid<br>PubMed Ovid	Title and abstract screening: one reviewer<br>Full text elegibility: one reviewer<br>Data extraction: two independent reviewers	No	No	Yes	Two independent reviewers	Loney, 1998	No	No	NA	No	NA	NA	NA	NA	NA	NA	NA	NA	NA
**Shafiee**	Prevalence of sarcopenia in the world: a systematic review and meta- analysis of general population studies	6	No	35	Incomplete	3	PubMed<br>Scopus<br>Web of Science	Study selection: two reviewers	No	No	Yes	Two reviewers	STROBE	No	No	NA	Yes	Random-effects	NR	Logit	Yes	No	No	I^2	Begg's test	STATA
**Shah**	Systematic review and meta-analysis: prevalence of small intestinal bacterial overgrowth in chronic liver disease	8	No	19	Yes	3	Embase<br>Medline Ovid<br>PubMed	Title and abstract screening: two independent reviewers<br>Data extraction: two independent reviewers	No	PRISMA<br>MOOSE	Yes	NR	Newcastle-Ottawa Scale	No	No	NA	No	NA	NA	NA	NA	NA	NA	NA	NA	NA
**Shams-Hosseini**	Prevalence of musculoskeletal disorders among dentists in Iran: a systematic review	5	NR	17	Incomplete	8	CINAHL<br>Google scholar<br>Irandoc<br>Iranmedex<br>Magiran<br>MEDLINE<br>Scopus<br>SID	Study selection: two independent reviewers	No	No	Yes	NR	STROBE (modified)	Yes	No	NA	Yes	Random-effects	NR	NR	No	Yes	No	I^2	No	STATA
**Sharifi**	Investigating the prevalence of preterm birth in Iranian population: a systematic review and meta-analysis	5	NR	19	Yes	9	Google Scholar<br>Irandoc<br>Iranmedex<br>Magiran<br>MEDLIB<br>PubMed<br>Scopus<br>SID<br>Web of science	Titles and abstracts screening: two independent reviewers<br>Full text elegibility: two independent reviewers<br>Data extraction: two independent reviewers	No	No	Yes	Two independent reviewers	Hoy, 2012	No	No	NA	Yes	Random-effects	NR	NR	No	Yes	No	Cochran's Q<br>I^2	No	STATA
**Shaw**	Chronic post-traumatic headache in children and adolescents: systematic review of prevalence and headache features	3	No	10	Incomplete	4	Cochrane<br>Embase<br>Google Scholar<br>PubMed	NR	No	PRISMA	No	NA	NA	NA	No	NA	No	NA	NA	NA	NA	NA	NA	NA	NA	NA
**Shawon**	Current evidence on prevalence and clinical outcomes of co-morbid obstructive sleep apnea and chronic obstructive pulmonary disease: a systematic review	6	No	27	Yes	1	PubMed	Title and abstract screening: two reviewers<br>Full text selection: two reviewers<br>Data extraction: two reviewers extracted and two reviewers verified extraction	No	MOOSE	Yes	Two reviewers	Newcastle-Ottawa Scale	No	No	NA	No	NA	NA	NA	NA	NA	NA	NA	NA	NA
**Silva**	Estimated prevalence of halitosis: a systematic review and meta-regression analysis	7	No	13	Yes	4	PubMed<br>Scielo<br>Scopus<br>Web of Science	Title and abstract screening: two independent reviewers<br>Full text elegibility: two independent reviewers<br>Data extraction: two independent reviewers	No	PRISMA	Yes	Two reviewers	JBI Prevalence Critical Appraisal Tool	No	No	NA	Yes	Random-effects	NR	NR	Yes	Yes	No	I^2<br>Sensitivity analysis	Egger's test<br>Funnel plot	STATA
**Smith**	Incidence and prevalence of patellofemoral pain: a systematic review and meta-analysis	9	Yes	23	Yes	4	CINAHL<br>Embase<br>PubMed<br>Web of Science	Title and abstract screening: one reviewer<br>Full text elegibility: two independent reviewers<br>Data extraction: one reviewer extracted, a second reviewer verified extraction	PROSPERO<br>CRD42016038870	PRISMA<br>MOOSE	Yes	Two independent reviewers	Tool from a previous systematic review of prevalence (Luime, 2004) (modified)	No	No	NA	Yes	Random-effects<br>Fixed effect	NR	NR	No	No	No	I^2	No	STATA
**Smythe**	Birth prevalence of congenital talipes equinovarus in low- and middle-income countries: a systematic review and meta-analysis	5	Yes	48	Yes	6	Africa Wide Information<br>CINAHL<br>EMBASE<br>Global Health<br>LILACS<br>Medline	Title and abstract screening: one reviewer<br>Full text elegibility: two independent reviewers<br>Data extraction: one reviewer extracted, a second reviewer verified extraction	No	MOOSE	Yes	NR	NR	Yes	No	NA	Yes	Fixed effect	NR	NR	No	No	No	I^2	No	STATA
**So**	Lateral lymph node metastasis in papillary thyroid carcinoma: a systematic review and meta-analysis for prevalence, risk factors, and location	4	No	23	Yes	2	Embase<br>PubMed	Study selection: two independent reviewers<br>Data extraction: two reviewers	No	PRISMA	Yes	NR	Newcastle-Ottawa Scale	No	No	NA	No	NA	NA	NA	NA	NA	NA	NA	NA	NA
**Song (A)**	Variations of pterygium prevalence by age, gender and geographic characteristics in China: a systematic review and meta-analysis	4	No	47	Incomplete	6	China National Knowledge Infrastructure (CNKI)<br>Chinese Biomedicine Literature Database (CBM-SinoMed)<br>Embase<br>Medline<br>PubMed<br>Wanfang	Study selection: two independent reviewers<br>Data extraction: two independent reviewers	No	PRISMA	No	NA	NA	NA	No	NA	Yes	Random-effects	DerSimonian and Laird	Logit	No	Yes	No	Cochran's Q<br>I^2	No	R

**Song (B)**	Prevalence of epilepsy in China between 1990 and 2015: a systematic review and meta-analysis	9	No	39	Yes	4	China National Knowledge Infrastructure (CNKI)<br>PubMed<br>VIP<br>Wanfang Data	Data extraction: at least two independent reviewers	No	PRISMA<br>GATHER	No	NA	NA	NA	No	NA	Yes	Random-effects	DerSimonian and Laird	NR	No	Yes	No	I^2	No	R
**Song (C)**	The prevalence of vitamin A deficiency in chinese children: a systematic review and bayesian meta-analysis	6	NR	54	Yes	6	China National Knowledge Infrastructure (CNKI),Wanfang data and Chinese Biomedicine Literature Database (CBM-SinoMed)) and three English databases (PubMed, Medline and Embase).	Title and abstract screening: two independent reviewers<br>Full text elegibility: two independent reviewers<br>Data extraction: two independent reviewers	No	PRISMA	No	NA	NA	NA	No	NA	Yes	Bayesian approach	NA	Logit	No	Yes	No	No	No	JAGS<br>R
**Spottswood**	The prevalence of posttraumatic stress disorder in primary care: a systematic review	3	Yes	41	Yes	6	CINAHL<br>Cochrane<br>Embase<br>Google Scholar<br>MEDLINE<br>PsycINFO	Title screening: one reviewer<br>Abstract screening: two independent reviewers<br>Full text selection: two reviewers<br>Data extraction: two reviewers	No	PRISMA	Yes	Two independent reviewers	Hoy, 2012 (modified)	No	No	NA	No	NA	NA	NA	NA	NA	NA	NA	NA	NA
**Stockdale**	Prevalence of hepatitis D virus infection in sub-Saharan Africa: a systematic review and meta-analysis	9	Yes	30	Yes	3	Embase<br>PubMed<br>Scopus	Study selection: two independent reviewers<br>Data extraction: one reviewer	No	PRISMA	Yes	Two independent reviewers	JBI Prevalence Critical Appraisal Tool (modified)	No	No	NA	Yes	Random-effects	DerSimonian and Laird	Freeman–Tukey double arcsine	Yes	Yes	No	I^2<br>Sensitivity analysis	Egger's test<br>Funnel plot	STATA
**Strasser**	Prevalence and risk factors for autism spectrum disorder in epilepsy: a systematic review and meta-analysis	5	NR	20	Incomplete	2	PsychINFO<br>PubMed	NR	No	No	Yes	NR	QUIPS tool (modified)	No	No	NA	Yes	Random-effects	DerSimonian and Laird	Raw	No	No	Yes	Cochran's Q<br>I^2	No	STATA
**Surda**	Prevalence of rhinitis in athletes: systematic review	4	NR	13	No	3	Embase (Ovid)<br>Medline (Ovid)<br>PubMed	Search: two reviewers<br>Abstract screening: two reviewers<br>Full text elegibility: two reviewers	No	PRISMA	Yes	NR	PRISMA<br>AMSTAR	No	No	NA	No	NA	NA	NA	NA	NA	NA	NA	NA	NA
**Sy**	Anticoagulation practices and the prevalence of major bleeding, thromboembolic events, and mortality in venoarterial extracorporeal membrane oxygenation: a systematic review and meta-analysis	5	No	26	Yes	6	CINHAHL<br>Cochrane<br>Embase<br>Medline<br>PubMed<br>PubMed Central	Title and abstract screening: two independent reviewers<br>Full text elegibility: two independent reviewers<br>Data extraction: at least two independent reviewers	No	PRISMA	Yes	NR	Newcastle-Ottawa Scale	No	No	NA	Yes	Random-effects	DerSimonian and Laird	Freeman–Tukey double arcsine	Yes	No	No	I^2<br>Sensitivity analysis	Egger's test<br>Funnel plot	STATA
**Tabrizi**	Prevalence of sleep quality disorder among Iranian drivers: a systematic review and meta-analysis	9	Yes	10	Incomplete	8	Google Scholar<br>Irandoc<br>Iranmedex<br>Magiran<br>PubMed<br>Scopus<br>SID<br>Web of Science	Search: two independent reviewers	No	No	Yes	NR	Tool from a previous systematic review of prevalence of prevalence (Moosazadeh, 2013)	Yes	No	NA	Yes	Random-effects	NR	NR	No	Yes	No	Cochran's Q<br>I^2<br>Sensitivity analysis	No	STATA
**Tavares**	HIV and tuberculosis co-infection among migrants in Europe: a systematic review on the prevalence, incidence and mortality	7	Yes	27	Yes	3	Medline<br>Scopus<br>Web of Science	Title and abstract screening: one reviewer screened, another reviewer screened a sample<br>Data extraction: one reviewer	No	PRISMA	No	NA	NA	NA	No	NA	No	NA	NA	NA	NA	NA	NA	NA	NA	NA
**Tengan**	Prevalence of hepatitis B in people living with HIV/AIDS in Latin America and the Caribbean: a systematic review and meta-analysis	5	No	28	Yes	3	Embase<br>LILACS<br>PubMed	Title and abstract screening: two independent reviewers<br>Full text elegibility: two reviewers<br>Data extraction: two independent reviewers	No	PRISMA	Yes	Two independent reviewers	New tool based on Boyle, 1998; Fowked and Fulton, 1991; Loney, 1998; and Prins, 2002	No	No	NA	yes	Random-effects	DerSimonian and Laird	NR	No	Yes	No	Cochran's Q<br>I^2	No	STATA
**Theroux**	Prevalence of low back pain in adolescents with idiopathic scoliosis: a systematic review	7	No	2	Yes	3	CINHAHL<br>Cochrane<br>PubMed	Title and abstract screening: two independent reviewers<br>Full text elegibility: two independent reviewers<br>Data extraction: one reviewer	No	PRISMA	Yes	Two independent reviewers	Hoy, 2012 (modified)	No	Yes	GRADE	No	NA	NA	NA	NA	NA	NA	NA	NA	NA
**Trompeter**	Prevalence of back pain in sports: a systematic review of the literature	3	No	43	Yes	6	Cochrane<br>Embase<br>Medline<br>PSYNDEX<br>PsycINFO<br>PubMed	Search: two independent reviewers<br>Data extraction: one reviewer extracted, a second reviewer verified extraction	No	PRISMA	Yes	Two independent reviewers	Tool from a previous systematic review of prevalence (Walker, 2000)	Yes	No	NA	No	NA	NA	NA	NA	NA	NA	NA	NA	NA
**Tubaishat**	Pressure ulcers prevalence in the acute care setting: a systematic review, 2000-2015	4	No	19	Incomplete	4	BNI<br>CINAHL<br>Cochrane<br>MEDLINE	Search: one reviewer	No	No	Yes	Two independent reviewers	Hawker, 2002	No	No	NA	No	NA	NA	NA	NA	NA	NA	NA	NA	NA
**Umer**	The prevalence of musculoskeletal symptoms in the construction industry: a systematic review and meta-analysis	5	NR	35	Unclear	9	Academic Premier<br>CINAHL<br>Health and safety science abstract<br>Medline<br>PsycINFO<br>Science Direct<br>Scopus<br>SportDiscus<br>Web of Science	Title and abstract screening: two independent reviewers<br>Full text elegibility: two independent reviewers<br>Data extraction: two independent reviewers	PROSPERO<br>CRD42016036051	PRISMA	Yes	Two independent reviewers	Loney, 1998 (modified)	No	No	NA	Yes	Random-effects	NR	NR	No	No	No	I^2	No	Review Manager
**VanderKruik**	The global prevalence of postpartum psychosis: a systematic review	6	Yes	6	Yes	6	CINAHL<br>Embase<br>Global Index Medicus<br>PsycINFO<br>PubMed<br>Sociological Collections	Title and abstract screening: two independent reviewers<br>Full text elegibility: two independent reviewers<br>Data extraction: two independent reviewers	No	PRISMA	Yes	Two independent reviewers	New tool	No	No	NA	No	NA	NA	NA	NA	NA	NA	NA	NA	NA
**Vikse**	The prevalence and morphometry of an accessory spleen: a meta-analysis and systematic review of 22,487 patients	8	No	81	Yes	6	BIOSIS<br>Embase<br>PubMed<br>Scielo<br>ScienceDirect<br>Web of Science	Study selection: three independent reviewers<br>Data extraction: three independent reviewers	No	PRISMA	No	NA	NA	NA	No	NA	Yes	Random-effects	NR	NR	Yes	No	No	Cochran's Q<br>I^2	No	MetaXL
**Vitalis**	Ethnic differences in the prevalence of peripheral arterial disease: a systematic review and meta-analysis	5	No	76	Incomplete	2	Embase<br>Medline	NR	PROSPERO<br>CRD42016041390	PRISMA	No	NA	NA	NA	No	NA	No	NA	NA	NA	NA	NA	NA	NA	NA	NA
**Wang**	Prevalence of Cryptosporidium, microsporidia and Isospora infection in HIV-infected people: a global systematic review and meta-analysis	7	Yes	131	Incomplete	7	Chinese Web of Knowledge<br>Chongqing VIP<br>Google Scholar<br>Embase<br>PubMed<br>ScienceDirect<br>Wanfang	Title and abstract screening: two independent reviewers<br>Data extraction: two independent reviewers	No	PRISMA	Yes	NR	GRADE (modified)	No	No	NA	Yes	Random-effects	NR	NR	Yes	Yes	No	Cochran's Q<br>I^2<br>Sensitivity analysis	No	STATA
**Wang (A)**	Changing trends in the prevalence of H. pylori infection in Japan (1908-2003): a systematic review and meta-regression analysis of 170,752 individuals	7	Yes	46	Yes	2	Embase<br>PubMed	Title and abstract screening: two independent reviewers<br>Full text elegibility: two independent reviewers	No	PRISMA	Yes	Two independent reviewers	JBI Prevalence Critical Appraisal Tool	No	No	NA	No	NA	NA	NA	NA	NA	NA	NA	NA	NA
**Wang (B)**	Prevalence of orofacial clefts among live births in China: a systematic review and meta-analysis	11	Yes	41	Yes	4	China National Knowledge Infrastructure (CNKI)<br>Embase<br>PubMed<br>Wanfang Database	Title and abstract screening: two independent reviewers<br>Full text elegibility: two reviewers<br>Data extraction: two independent reviewers	PROSPERO<br>CRD42015030198	No	Yes	NR	Hoy, 2012 (modified)	No	No	NA	Yes	Random-effects	NR	NR	Yes	No	No	Cochran's Q<br>I^2	Egger's test<br>Funnel plot	R
**Wang (C)**	Prevalence of depression and depressive symptoms among outpatients: a systematic review and meta-analysis	12	Yes	83	Yes	4	Cochrane<br>EMBASE<br>PsycINFO<br>PubMed	Study selection: two independent reviewers<br>Data extraction: two independent reviewers	PROSPERO<br>CRD42017054738	PRISMA	Yes	NR	Newcastle-Ottawa Scale (modified)	No	No	NA	Yes	Random-effects	NR	NR	Yes	Yes	No	Cochran's Q<br>I^2<br>Sensitivity analysis	Egger's test<br>Funnel plot	R
**Wang (D)**	Prevalence and burden of Toxoplasma gondii infection in HIV-infected people: a systematic review and meta-analysis	8	Yes	74	Incomplete	7	Chinese Web of Knowledge<br>Chongqing VIP<br>Embase<br>Google Scholar<br>PubMed<br>ScienceDirect<br>Wanfang	Title and abstract screening: two independent reviewers<br>Full text elegibility: two independent reviewers<br>Data extraction: two independent reviewers	No	PRISMA	Yes	NR	GRADE (modified)	No	No	NA	Yes	Random-effects	NR	NR	Yes	Yes	No	Cochran's Q<br>I^2	No	STATA
**Williamson**	Prevalence of mental health disorders in elderly U.S. military veterans: a meta-analysis and systematic review	4	Yes	11	Yes	6	EMBASE<br>Google Scholar<br>PILOTS<br>PsycINFO<br>PubMed<br>Web of Science	Study selection: two independent reviewers<br>Data extraction: two independent reviewers	No	No	Yes	Two independent reviewers	NIH Quality Assessment Tool for Observational Cohort and Cross-Sectional Studies	No	No	NA	Yes	Random-effects	NR	Double arcsine	No	No	No	Cochran's Q<br>I^2	Begg's test<br>Egger's test<br>Funnel plot	MetaXL
**Wojtera**	The prevalence of human papillomavirus in pediatric tonsils: a systematic review of the literature	7	No	11	Incomplete	5	CINAHL<br>Cochrane<br>Embase<br>PubMed<br>Scopus	Search: two independent reviewers<br>Data extraction: two independent reviewers	No	PRISMA	Yes	Two reviewers	Tool from a previous systematic review of prevalence (Modesti, 2016)<br>Oxford Centre for Evidence-Based Medicine levels of evidence	No	No	NA	No	NA	NA	NA	NA	NA	NA	NA	NA	NA
**Wolke**	Systematic review and meta-analysis: fussing and crying durations and prevalence of colic in infants	5	NR	28	Yes	3	Embase<br>MEDLINE<br>PsycINFO	Abstract screening: one reviewer (2 reviewers, each one screened half the abstracts)<br>Full text elegibility: two independent reviewers<br>Data extraction: two independent reviewers	No	PRISMA	Yes	NR	New tool	No	No	NA	No	NA	NA	NA	NA	NA	NA	NA	NA	NA
**Wolpe**	Prevalence of female sexual dysfunction in Brazil: a systematic review	5	No	20	Yes	4	CINAHL<br>LILACS<br>PubMed<br>Scopus	Title and abstract screening: two independent reviewers<br>Data extraction: two reviewers	No	PRISMA	Yes	Two independent reviewers	Kmet, 2004	No	No	NA	No	NA	NA	NA	NA	NA	NA	NA	NA	NA
**Woody**	A systematic review and meta-regression of the prevalence and incidence of perinatal depression	5	NR	96	Yes	2	PsycINFO<br>PubMed	Study selection: one reviewer selected, a second reviewer verified a sample (10%)<br>Data extraction: one reviewer extracted, a second reviewer verified a sample (10%)	No	PRISMA	No	NA	NA	NA	No	NA	Yes	Random-effects	NR	Log	Yes	Yes	No	I^2	Funnel plot	STATA
**Workneh**	Prevalence and associated factors of tuberculosis and diabetes mellitus comorbidity: a systematic review	3	No	94	Yes	6	Cochrane<br>Embase<br>Global Health<br>Medline (Ovid)<br>Scopus<br>Web of Science	Data extraction: one reviewer	No	PRISMA	Yes	One reviewer	New tool	No	No	NA	No	NA	NA	NA	NA	NA	NA	NA	NA	NA
**Wu**	Socioeconomic status and prevalence of type 2 diabetes in mainland China, Hong Kong and Taiwan: a systematic review	5	Yes	33	Yes	3	Embase<br>Global Health<br>Medline	Title and abstract screening: two reviewers<br>Full text elegibility: two reviewers<br>Data extraction: two independent reviewers	PROSPERO<br>CRD42016047913	PRISMA	Yes	Two independent reviewers	Newcastle-Ottawa Scale (modified)	No	No	NA	No	NA	NA	NA	NA	NA	NA	NA	NA	NA
**Xiao**	Prevalence and risk factors of epiretinal membranes: a systematic review and meta-analysis of population-based studies	5	Yes	13	Yes	3	Embase<br>PubMed<br>Web of Science	Study selection: two independent reviewers<br>Data extraction: two independent reviewers	No	PRISMA	Yes	Two independent reviewers	Tool from previous systematic reviews of prevalence (de Weer, 2009; Rogers, 2010)	No	No	NA	Yes	Random-effects	NR	NR	Yes	No	No	I^2	No	STATA
**Xie**	Prevalence and risk factors associated with musculoskeletal complaints among users of mobile handheld devices: a systematic review	3	NR	15	Yes	5	CINAHL<br>Embase<br>Medline (Ovid)<br>PubMed<br>Web of Science	Study assessment: two independent reviewers	No	No	Yes	Two independent reviewers	Hoy, 2012<br>SIGN checklists for case-control and cohort studies	No	No	NA	No	NA	NA	NA	NA	NA	NA	NA	NA	NA
**Xu**	Global prevalence of hypertension among people living with HIV: a systematic review and meta-analysis	3	NR	49	Yes	2	PubMed<br>Web of Science	Data extraction: two independent reviewers	No	PRISMA	Yes	NR	JBI Prevalence Critical Appraisal Tool	No	No	NA	Yes	Random-effects	NR	NR	Yes	No	No	Cochran's Q<br>I^2	No	R
**Yang**	Prevalence, risk factors and characteristics of the 'adding-on' phenomenon in idiopathic scoliosis after correction surgery: a systematic review and meta-analysis	6	No	35	Yes	5	China WeiPu Library<br>Cochrane<br>Embase<br>PubMed<br>Web of Science	Study screeening: two reviewers<br>Data extraction: two reviewers	No	No	Yes	NR	Newcastle-Ottawa Scale	No	No	NA	Yes	NR	NR	NR	Yes	No	No	Cochran's Q<br>I2<br>Sensitivity analysis	No	STATA

**Yao**	Prevalence of abdominal artery calcification in dialysis patients with end-stage renal disease: a systematic review and meta-analysis	6	Yes	44	Yes	3	Embase<br>PubMed<br>Web of Science	Study selection: two independent reviewers<br>Data extraction: two independent reviewers	PROSPERO<br>CRD42017055492	PRISMA<br>MOOSE	Yes	NR	NIH Quality Assessment Tool for Observational Cohort and Cross-Sectional Studies (modified)	No	No	NA	Yes	Random-effects	NR	NR	Yes	Yes	No	I^2	Begg's test<br>Egger's test	STATA
**Zhang**	Prevalence of depression and anxiety in systemic lupus erythematosus: a systematic review and meta-analysis	5	Yes	59	Yes	4	Cochrane<br>Embase<br>PsycINFO<br>PubMed	Title and abstract screening: two independent reviewers<br>Full text elegibility: two reviewers<br>Data extraction: two independent reviewers	PROSPERO<br>CRD42016044125	PRISMA	Yes	Two reviewers	Newcastle-Ottawa Scale (modified)	No	No	NA	Yes	Random-effects	NR	NR	Yes	No	No	I^2<br>Sensitivity analysis	Egger's test<br>Funnel plot	STATA
**Zheng**	The prevalence of depression and depressive symptoms among eye disease patients: a systematic review and meta-analysis	4	Yes	28	Yes	4	Cochrane Library<br>Embase<br>Medline<br>PubMed	Search: two independent reviewers	No	PRISMA	Yes	NR	Newcastle-Ottawa Scale	No	No	NA	Yes	Random-effects	NR	NR	Yes	No	No	Cochran's Q<br>I^2	No	R<br>STATA
**Zhou**	Prevalence, risk factors, and prognosis of orthostatic hypotension in diabetic patients: a systematic review and meta-analysis	4	Yes	21	Incomplete	6	China National Knowledge Infrastructure<br>Embase<br>PubMed<br>SINOMED<br>VIP Chinese Journal<br>Wanfang	Search: two independent reviewers<br>Data extraction: two independent reviewers	No	No	Yes	NR	STROBE	No	No	NA	Yes	Random-effects	DerSimonian and Laird	NR	Yes	No	No	Cochran's Q<br>I^2<br>Sensitivity analysis	Begg's test<br>Egger's test	STATA
