# Supplementary material for: How are systematic reviews of prevalence conducted? A methodological study
Source: BMC Med Res Methodol. 2020 Apr 26;20:96. doi: 10.1186/s12874-020-00975-3 (PMC7184711; doi:10.1186/s12874-020-00975-3)
Supplement: Supplementary file 4 — Additional file 4. Journals of publication of included systematic reviews. [file 12874_2020_975_MOESM4_ESM.pdf]

Additional file 4: Journals of publication of included systematic reviews

Journal	Number of reviews
PLoS One	8
BMJ Open	7
Lancet Global Health	5
Medicine	5
Sleep Medicine Reviews	4
International Journal of STDs and AIDS	3
Journal of affective disorders	3
Oncotarget	3
Scientific Reports - Nature	3
Seminars in Arthritis and Rheumatism	3
Archives of Gynecology and Obstetrics	2
BMC Musculoskeletal Disorders	2
BMC Psychiatry	2
Clinical Oral Investigations	2
Critical Reviews in Food Science and Nutrition	2
Developmental Medicine & Child Neurology	2
Diabetic Medicine	2
Gastroenterology	2
International Journal of Ophthalmology	2
International Journal of Surgery	2
International Urology and Nephrology	2
Journal of Global Health	2
Journal of Hypertension	2
Journal of Pain	2
Nutrients	2
Rheumatology (Oxford)	2
Rheumatology International	2
Acta Neurochirurgic	1
Acta Obstetricia et Gynecologica Scandinavica	1
Acta Odontologica Scandinavica	1
Advances in Pediatric Research	1
Aesthetic Plastic Surgery	1
Aging & Mental Health	1
Air Medical Journal	1
Allergy	1
Am J Geriatr Psychiatry	1
American Journal of Emergency Medicine	1

American Journal of Infection Control	1
Annals of Plastic Surgery	1
Annals of Surgery	1
Applied Ergonomics	1
Archives of Iranian medicine	1
Archives of Orthopaedic and Trauma Surgery	1
Archives of Psychiatric Nursing	1
Archives of Public Health	1
Archives of Women's Mental Health	1
Asia Pacific Journal of Clinical Nutrition	1
Basic and Clinical Neuroscience	1
Biochemistry and Cell Biology	1
Birth Defects Research	1
BMC Health Services Research	1
BMC Hematology	1
BMC Infectious Diseases	1
BMC Medical Education	1
BMC Medical Genetics	1
BMC Nursing	1
BMC Pregnancy Childbirth	1
BMC Pulmonary Medicine	1
BMJ Open Sport & Exercise Medicine	1
British Journal of Dermatology	1
British Journal of Sports Medicine	1
Burns	1
Chest	1
Chiropractic & Manual Therapies	1
Cleft Palate–Craniofacial Journal	1
Clinical and Experimental Rheumatology	1
Clinical Gastroenterology and Hepatology	1
Clinical Nursing Research	1
Clinical Nutrition	1
Clinical Psychology Review	1
Clinical Respiratory Journal	1
Current HIV Research	1
Diabetes & Metabolic Syndrome: Clinical Research & Reviews	1
Diabetes Research and Clinical Practice	1
Drug and Alcohol Dependence	1
Drug and Alcohol Review	1
Dysphagia	1
Electronic Physician	1
Epidemiology & Infection	1

Epidemiology and Health	1
European Journal of Clinical Microbiology & Infectious Diseases	1
European Journal of Clinical Pharmacology	1
European Journal of Gastroenterology & Hepatology	1
European Journal of Obstetrics & Gynecology and Reproductive Biology	1
European Journal of Pain	1
European Journal of Surgical Oncology	1
European Urology	1
Expert Review Cardiovascular Therapy	1
Frontiers in Psychiatry	1
Frontiers in psychology	1
Harvard Review of Psychiatry	1
Heart Views	1
Helicobacter	1
Human Reproduction Update	1
Indian Journal of Medical Microbiology	1
Indian Journal of Medical Research	1
Intellectual and Developmental Disabilities	1
International Archives of Occupational and Environmental Health	1
International Journal of Cardiology	1
International Journal of Geriatric Psychiatry	1
International Journal of Infectious Diseases	1
International Journal of Molecular Sciences	1
International Journal of Otolaryngology	1
International Journal of Reproductive Biomedicine	1
International Nursing Review	1
International Psychogeriatric Association	1
International Society of Behavioral Medicine	1
Iranian Journal of Child Neurology	1
Iranian Journal of Kidney Diseases	1
Iranian Journal of Neurology	1
Iranian Journal of Public Health	1
JAMA Pediatrics	1
JAMA Surgery	1
JBI Database of Systematic Reviews and Implementation Reports	1
Journal of Antimicrobial Chemotherapy	1
Journal of Caring Sciences	1
Journal of Clinical and Diagnostic Research for doctors	1
Journal of Cranio-Maxillo-Facial Surgery	1
Journal of Critical Care	1
Journal of Crohn's and Colitis	1
Journal of Diabetes & Metabolic Disorders	1

Journal of Diabetes Investigation	1
Journal of Eating Disorders	1
Journal of Epidemiology	1
Journal of Evidence Based Medicine	1
Journal of Gambling Studies	1
Journal of Global Antimicrobial Resistance	1
Journal of Head Trauma Rehabilitation	1
Journal of Helminthology	1
Journal of Injury and Violence Research	1
Journal of Neurosurgery	1
Journal of Otolaryngology- Head & Neck Surgery	1
Journal of Pediatrics	1
Journal of Research in Medical Scien	1
Journal of Substance Abuse Treatment	1
Journal of the American Society of Hypertension	1
Journal of the European Academy of Dermatology and Venereology	1
Journal of Tropical Pediatrics	1
Journal of Voice	1
Journal Wound Care	1
Lancet	1
Lancet Gastroenterology & Hepatology	1
Lancet HIV	1
Lancet Public Health	1
Materia Socio Medica	1
Maturitas	1
Menopause	1
Microbial Pathogenesis	1
Middle East African Journal of Ophthalmology	1
Minerva Urologica e Nefrologica	1
Multiple Sclerosis and Related Disorders	1
Neuropathology and Applied Neurobiology	1
Neuropsychiatric Disease and Treatment	1
Neurourology and Urodynamics	1
Osong Public Health and Research Perspectives	1
Pain	1
Pain Management	1
Pain Medicine	1
Pain Physician	1
Parasites & Vectors	1
Parasitology	1
Pediatric Blood Cancer	1
Pediatric Infectious Disease Journal	1

Pediatric Research	1
Psychiatry Research	1
Psychooncology	1
Reproductive Toxicology	1
Research in Nursing & Health	1
Respirology	1
Retina	1
Revista da Sociedade Brasileira de Medicina Tropical	1
Seminars in Liver Disease	1
Social Psychiatry and Psychiatric Epidemiology	1
Spine	1
Sports Medicine	1
Support Care Cancer	1
Systematic Reviews	1
The American Journal of Sports Medicine	1
The British Journal of Psychiatry	1
Transfusion and Apheresis Science	1
Tropical Medicine & International Health	1
Virologia sinica	1
Wellcome Open Research	1
Wiener klinische Wochenschrift	1
